# Supplementary material for: Genetic diversity and population structure analysis of soybean [Glycine max (L.) Merrill] genotypes based on agro-morphological traits and SNP markers
Source: PLoS One. 2025 Oct 10;20(10):e0332895. doi: 10.1371/journal.pone.0332895 (PMC12513657; doi:10.1371/journal.pone.0332895)
Supplement: S3 Table — RtNd = root nodule score, Lodg = lodging score, D50F = days to 50% flowering, PH = plant height in centimeter, D95M = days to 95% maturity, SHS = shattering score, GY = grain yield in kilogram per hectare, SE = standard error, LSD = least significant difference. (DOCX) [file pone.0332895.s003.docx]

| **Genotype** | **RtNod** | **Lodg** | **D50F** | **PH** | **D95M** | **SHS** | **GY** |
| --- | --- | --- | --- | --- | --- | --- | --- |
| G01 | 3.50 | 2.40 | 52 | 82.5 | 119 | 1.16 | 3050 |
| G02 | 2.50 | 2.04 | 48 | 63.6 | 117 | 1.84 | 3310 |
| G03 | 3.17 | 1.80 | 52 | 64.8 | 118 | 1.02 | 2610 |
| G04 | 3.17 | 1.85 | 55 | 81.1 | 118 | 1.34 | 2150 |
| G05 | 3.61 | 1.95 | 55 | 82.5 | 114 | 1.83 | 2860 |
| G06 | 2.89 | 2.11 | 54 | 86.3 | 115 | 1.01 | 2570 |
| G07 | 3.50 | 1.66 | 52 | 70.3 | 114 | 0.97 | 2410 |
| G08 | 3.22 | 2.30 | 55 | 72.5 | 118 | 1.13 | 2740 |
| G09 | 3.33 | 2.24 | 50 | 75.4 | 119 | 1.19 | 2790 |
| G10 | 3.00 | 1.84 | 53 | 76.7 | 118 | 1.50 | 3210 |
| G11 | 3.39 | 2.07 | 52 | 91.9 | 116 | 1.00 | 3060 |
| G12 | 3.39 | 1.69 | 54 | 68.3 | 119 | 1.15 | 2380 |
| G13 | 2.67 | 2.16 | 55 | 71.3 | 113 | 1.52 | 2300 |
| G14 | 3.22 | 1.55 | 48 | 74.8 | 114 | 1.85 | 2800 |
| G15 | 3.50 | 1.38 | 48 | 90.5 | 119 | 1.03 | 2020 |
| G16 | 3.61 | 1.29 | 49 | 96.4 | 119 | 1.24 | 2420 |
| G17 | 3.00 | 1.06 | 48 | 102 | 118 | 1.26 | 2550 |
| G18 | 3.22 | 1.18 | 50 | 83.0 | 120 | 1.03 | 2040 |
| G19 | 3.28 | 1.44 | 48 | 95.2 | 118 | 1.15 | 2460 |
| G20 | 2.89 | 1.02 | 49 | 98.6 | 118 | 1.15 | 2600 |
| G21 | 2.28 | 2.00 | 50 | 71.9 | 120 | 1.33 | 2240 |
| G22 | 2.89 | 1.00 | 50 | 90.6 | 119 | 1.16 | 2360 |
| G23 | 2.17 | 1.99 | 44 | 77.5 | 114 | 1.16 | 2310 |
| G24 | 3.56 | 1.11 | 46 | 86.0 | 116 | 1.77 | 2990 |
| G25 | 3.11 | 1.51 | 51 | 86.0 | 121 | 1.01 | 2250 |
| G26 | 3.28 | 1.11 | 48 | 94.3 | 117 | 1.00 | 2590 |
| G27 | 3.33 | 1.76 | 53 | 89.0 | 118 | 0.97 | 2530 |
| G28 | 3.11 | 1.29 | 52 | 72.2 | 120 | 1.16 | 2140 |
| G29 | 2.78 | 1.77 | 47 | 71.1 | 115 | 1.00 | 2540 |
| G30 | 3.17 | 1.82 | 48 | 70.9 | 114 | 1.04 | 2530 |
| G31 | 3.06 | 1.56 | 49 | 67.4 | 114 | 1.01 | 2580 |
| G32 | 2.39 | 1.99 | 47 | 69.7 | 112 | 1.33 | 2690 |
| G33 | 2.61 | 2.04 | 45 | 71.5 | 112 | 1.15 | 2960 |
| G34 | 3.28 | 1.24 | 48 | 87.3 | 119 | 1.32 | 2440 |
| G35 | 3.22 | 1.95 | 50 | 69.2 | 120 | 1.00 | 1700 |
| G36 | 3.00 | 1.55 | 49 | 72.6 | 117 | 2.33 | 2600 |
| G37 | 2.72 | 1.91 | 49 | 74.9 | 114 | 1.25 | 2590 |

Table: cont’d

| **Genotype** | **RtNod** | **Lodg** | **D50F** | **PH** | **D95M** | **SHS** | **GY** |
| --- | --- | --- | --- | --- | --- | --- | --- |
| G38 | 3.28 | 1.68 | 50 | 86.6 | 115 | 1.20 | 2600 |
| G39 | 3.56 | 1.27 | 46 | 89.8 | 117 | 1.50 | 2210 |
| G40 | 3.28 | 1.49 | 52 | 73.4 | 117 | 0.97 | 2680 |
| G41 | 2.94 | 1.64 | 54 | 69.8 | 119 | 1.17 | 2460 |
| G42 | 3.44 | 2.25 | 52 | 80.8 | 116 | 1.51 | 2410 |
| G43 | 3.33 | 1.73 | 50 | 79.9 | 115 | 0.96 | 2440 |
| G44 | 3.44 | 2.43 | 48 | 91.1 | 116 | 1.50 | 2200 |
| G45 | 3.56 | 1.76 | 49 | 73.3 | 117 | 3.50 | 2770 |
| **Mean** | 3.13 | 1.71 | 50.1 | 79.9 | 117 | 1.30 | 2540 |
| **SE**  **LSD** | 0.01  0.71 | 0.01  0.54 | 0.06  2.24 | 0.22  9.50 | 0.05  2.38 | 0.01  0.67 | 7.30  559 |
