# Supplementary figures and images for: Genetic diversity and population structure analysis of soybean [Glycine max (L.) Merrill] genotypes based on agro-morphological traits and SNP markers
Source: PLoS One. 2025 Oct 10;20(10):e0332895. doi: 10.1371/journal.pone.0332895 (PMC12513657; doi:10.1371/journal.pone.0332895)

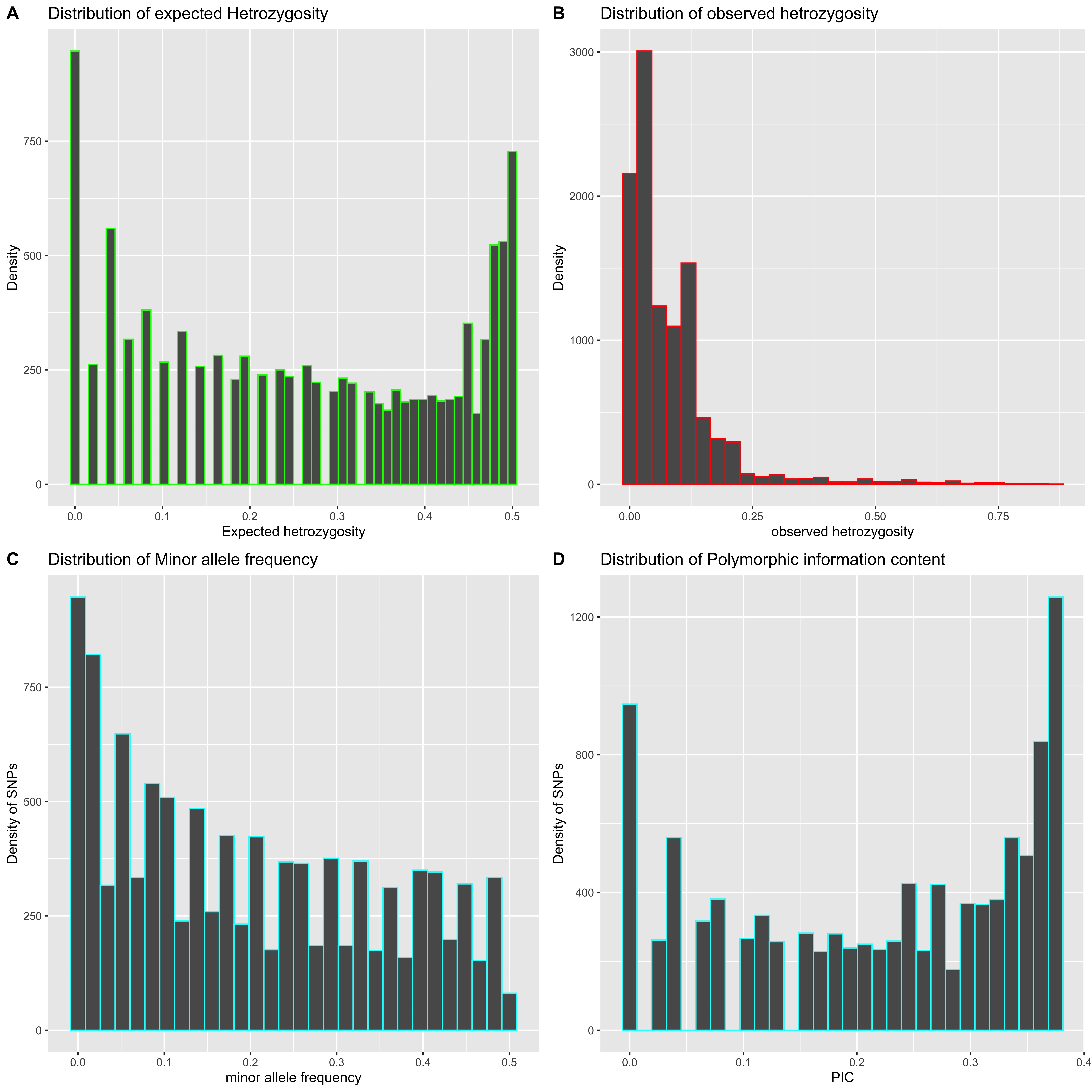

Supplement: S1 Fig — (PNG) [file pone.0332895.s006.png]
