# Supplementary material for: Genetic diversity and population structure analysis of soybean [Glycine max (L.) Merrill] genotypes based on agro-morphological traits and SNP markers
Source: PLoS One. 2025 Oct 10;20(10):e0332895. doi: 10.1371/journal.pone.0332895 (PMC12513657; doi:10.1371/journal.pone.0332895)
Supplement: Attachment — Submitted filename: PONE-D-25-08250 revised2.pdf [file pone.0332895.s009.pdf]

PLOS ONE

Genetic diversity and population structure analysis of soybean [Glycine max (L.) Merrill] genotypes based on agro-morphological traits and SNP markers

--Manuscript Draft--

**Manuscript Number:**	PONE-D-25-08250R1
**Article Type:**	Research Article
**Full Title:**	Genetic diversity and population structure analysis of soybean [Glycine max (L.) Merrill] genotypes based on agro-morphological traits and SNP markers
**Short Title:**	Genetic diversity and population structure analysis of soybean genotypes in Nigeria.
**Corresponding Author:**	Abebawork Tilahun Assfaw, MSc<br>Hawassa University College of Agriculture<br>Hawassa, Sidama National Regional State ETHIOPIA
**Keywords:**	principal component analysis; cluster analysis; SNP marker; Minor allele frequency; Admixture
**Abstract:**	Soybean (Glycine max) is one of the world's most important oil seed crops and has adapted to various environmental conditions. Yields of soybeans in Nigeria are notably low due to different production constraints, including the limited availability of improved varieties and the slow replacement rate of old varieties with new and high-yielding ones. Ensuring high genetic diversity in the working germplasm is among the primary factors for the success of the breeding programs in identifying high-yielding and well-adapted improved varieties. This study aimed to assess the genetic diversity and population structure of 45 soybean breeding lines of the International Institute of Tropical Agriculture soybean breeding program at the advanced evaluation stage based on phenotypic traits and SNP markers to support breeding strategies. A total of 45 soybean genotypes were laid out in a 5 x 9 alpha-lattice design, with three replications in 2022 across three of the IITA stations in Nigeria. The collected yield and yield component data were subjected to multi-variate analyses i.e., principal component and cluster analyses using R software. The genotypes were further assessed for SNPs-based genotypic diversity analyses. Principal component analysis revealed that the first three principal components accounted for 64.8% of the total variation. Hierarchical cluster analysis based on phenotypic data identified five cluster groups; while three clusters were identified with the corresponding SNP markers. The genotypes G02, G23, G29, G30, G31, G32, G33, G37 of cluster II and G45, G24, G14, G36 of cluster IV showed the maximum desirable traits which could be considered in the selection of parents for future IITA soybean breeding program. The SNP markers showed average observed heterozygosity, expected heterozygosity, minor allele frequency, and polymorphic information content of 0.08, 0.27, 0.20, and 0.22, respectively, which showed the existence of considerable genetic variation among the studied genotypes. The current study suggested that the genotypes under investigation exhibited a high genetic variation in the studied genotypes.
**Order of Authors:**	Abebawork Tilahun Assfaw, MSc
	Olasanmi Bunmi
	Agre Paterne
	Chigeza Godfree
	Mushoriwa Hapson
	Kayode Fowobaje
	Abush Tesfaye Abebe
**Response to Reviewers:**	Response: Thank you for the valuable comment. Accordingly, we have added the ANOVA table and mean comparison results for the phenotypic traits (see new Table 4 and S3 Table in the Results section). Although we did not conduct testing on the data for homogeneity and normality before the ANOVA analysis because the fitted linear mixed-effect model (using the lmerTest package in R) is robust to violations of both homogeneity of variance and normality. This model can explicitly handle

Powered by Editorial Manager® and ProduXion Manager® from Aries Systems Corporation

	heteroscedasticity through the inclusion of random effects and effectively handles deviations from normality, thus ensuring the validity and reliability of our results without requiring separate assumption testing.<br>Response: The sentence has been revised accordingly in the introduction.
Additional Information:	
Question	Response
**Financial Disclosure**<br><br>Enter a financial disclosure statement that describes the sources of funding for the work included in this submission. Review the submission guidelines for detailed requirements. View published research articles from *PLOS ONE* for specific examples.<br><br>This statement is required for submission and **will appear in the published article** if the submission is accepted. Please make sure it is accurate.<br><br>**Funded studies**<br>Enter a statement with the following details:<br>• Initials of the authors who received each award<br>• Grant numbers awarded to each author<br>• The full name of each funder<br>• URL of each funder website<br>• Did the sponsors or funders play any role in the study design, data collection and analysis, decision to publish, or preparation of the manuscript?<br><br>Did you receive funding for this work?	Yes
Please add funding details.<br>as follow-up to "**Financial Disclosure**<br><br>Enter a financial disclosure statement that describes the sources of funding for the work included in this submission. Review the submission guidelines for detailed requirements. View published research articles from *PLOS ONE* for specific examples.<br><br>This statement is required for submission and **will appear in the published article** if	This research was funded by the Pan African University Life and Earth Science Institute (including Health and Agriculture) (PAULESI) as part of the MSc funding awarded to ATA with grant number PAU020110MB (https://www.pau-au.africa/institues/paulesi). The study was also supported by the International Institute of Tropical Agriculture (IITA) in connection with the United States Agency for International Development (IITA/USAID) Genetic Improvement in Soy Project with number PJ-2315 (https://www.iita.org/iita-countries/nigeria/). The funders had no role in study design, data collection and analysis, decision to publish, or preparation of the manuscript.

Powered by Editorial Manager® and ProduXion Manager® from Aries Systems Corporation

the submission is accepted. Please make sure it is accurate.<br><br>**Funded studies**<br>Enter a statement with the following details:<br>• Initials of the authors who received each award<br>• Grant numbers awarded to each author<br>• The full name of each funder<br>• URL of each funder website<br>• Did the sponsors or funders play any role in the study design, data collection and analysis, decision to publish, or preparation of the manuscript?<br><br>Did you receive funding for this work?"	
Please select the country of your main research funder (please select carefully as in some cases this is used in fee calculation).<br>as follow-up to "**Financial Disclosure**<br><br>Enter a financial disclosure statement that describes the sources of funding for the work included in this submission. Review the submission guidelines for detailed requirements. View published research articles from *PLOS ONE* for specific examples.<br><br>This statement is required for submission and **will appear in the published article** if the submission is accepted. Please make sure it is accurate.<br><br>**Funded studies**<br>Enter a statement with the following details:<br>• Initials of the authors who received each award<br>• Grant numbers awarded to each author<br>• The full name of each funder<br>• URL of each funder website<br>• Did the sponsors or funders play any role in the study design, data collection and analysis, decision to publish, or preparation of the manuscript?	NIGERIA - NG

Powered by Editorial Manager® and ProduXion Manager® from Aries Systems Corporation

Did you receive funding for this work?"	
**Competing Interests**<br><br>Use the instructions below to enter a competing interest statement for this submission. On behalf of all authors, disclose any competing interests that could be perceived to bias this work—acknowledging all financial support and any other relevant financial or non-financial competing interests.<br><br>This statement is required for submission and **will appear in the published article** if the submission is accepted. Please make sure it is accurate and that any funding sources listed in your Funding Information later in the submission form are also declared in your Financial Disclosure statement.<br><br>View published research articles from *PLOS ONE* for specific examples.<br><br>**NO authors have competing interests**<br><br>Enter: *The authors have declared that no competing interests exist.*<br><br>**Authors with competing interests**<br><br>Enter competing interest details beginning with this statement:<br><br>*I have read the journal's policy and the authors of this manuscript have the following competing interests: [insert competing interests here]*<br><br>* typeset	The authors have declared that no competing interests exist.
**Ethics Statement**<br><br>Enter an ethics statement for this submission. This statement is required if the study involved:<br><br>• Human participants<br>• Human specimens or tissue	N/A

- Vertebrate animals or cephalopods
- Vertebrate embryos or tissues
- Field research

Write "N/A" if the submission does not require an ethics statement.

General guidance is provided below. Consult the submission guidelines for detailed instructions. **Make sure that all information entered here is included in the Methods section of the manuscript.**

Powered by Editorial Manager® and ProduXion Manager® from Aries Systems Corporation

**Format for specific study types**<br><br>**Human Subject Research (involving human participants and/or tissue)**<br>• Give the name of the institutional review board or ethics committee that approved the study<br>• Include the approval number and/or a statement indicating approval of this research<br>• Indicate the form of consent obtained (written/oral) or the reason that consent was not obtained (e.g. the data were analyzed anonymously)<br><br>**Animal Research (involving vertebrate animals, embryos or tissues)**<br>• Provide the name of the Institutional Animal Care and Use Committee (IACUC) or other relevant ethics board that reviewed the study protocol, and indicate whether they approved this research or granted a formal waiver of ethical approval<br>• Include an approval number if one was obtained<br>• If the study involved *non-human primates*, add *additional details* about animal welfare and steps taken to ameliorate suffering<br>• If anesthesia, euthanasia, or any kind of animal sacrifice is part of the study, include briefly which substances and/or methods were applied<br><br>**Field Research**<br><br>Include the following details if this study involves the collection of plant, animal, or other materials from a natural setting:<br>• Field permit number<br>• Name of the institution or relevant body that granted permission	
**Data Availability**<br><br>Authors are required to make all data underlying the findings described fully available, without restriction, and from the time of publication. PLOS allows rare exceptions to address legal and ethical concerns. See the PLOS Data Policy and FAQ for detailed information.	Yes - all data are fully available without restriction

A Data Availability Statement describing where the data can be found is required at submission. Your answers to this question constitute the Data Availability Statement and **will be published in the article**, if accepted.<br><br>**Important:** Stating 'data available on request from the author' is not sufficient. If your data are only available upon request, select 'No' for the first question and explain your exceptional situation in the text box.<br><br>Do the authors confirm that all data underlying the findings described in their manuscript are fully available without restriction?	
**Describe where the data may be found in full sentences. If you are copying our sample text, replace any instances of XXX with the appropriate details.**<br><br>• If the data are **held or will be held in a public repository**, include URLs, accession numbers or DOIs. If this information will only be available after acceptance, indicate this by ticking the box below. For example: *All XXX files are available from the XXX database (accession number(s) XXX, XXX.*).<br>• If the data are all contained **within the manuscript and/or Supporting Information files**, enter the following: *All relevant data are within the manuscript and its Supporting Information files.*<br>• If neither of these applies but you are able to provide **details of access elsewhere**, with or without limitations, please do so. For example:<br><br>*Data cannot be shared publicly because of [XXX]. Data are available from the XXX Institutional Data Access / Ethics Committee (contact via XXX) for researchers who meet the criteria for access to confidential data.*<br><br>*The data underlying the results presented in the study are available from (include the name of the third party*	The data underlying the results presented in the study are available from IITA Data Bank, https://data.iita.org/ with the associated DOI, https://doi.org/10.25502/mja8-g609/d

and contact information or URL).<br>• This text is appropriate if the data are owned by a third party and authors do not have permission to share the data.<br><br>* typeset	
Additional data availability information:	Tick here if the URLs/accession numbers/DOIs will be available only after acceptance of the manuscript for publication so that we can ensure their inclusion before publication.

Powered by Editorial Manager® and ProduXion Manager® from Aries Systems Corporation

Manuscript Click here to access/download;Manuscript;Manuscript.docx

Genetic diversity and population structure analysis of soybean [*Glycine max* (L.) Merrill] genotypes based on agro-morphological traits and SNP markers

Abebawork Tilahun Assfaw[1,2*], Bunmi Olasanmi[1,3], PaterneAgre[4], Godfree Chigeza[4], Hapson Mushoriwa[4], Fowobaje Kayode[4], Abush Tesfaye Abebe[4]

[1]Pan African University Life and Earth Science Institute (including Health and Agriculture), Ibadan, Nigeria

[2]Hawassa University College of Agriculture, Hawassa, Ethiopia

[3]Department of Crop and Horticultural Sciences, University of Ibadan, Ibadan, Nigeria

[4]International Institute of Tropical Agriculture, Ibadan, Nigeria

Corresponding author

E-mail: abebaworktilahun@gmail.com (ATA)

Abstract

Soybean (*Glycine max*) is one of the world's most important oilseed crops and has adapted to various environmental conditions. Yields of soybeans in Nigeria are notably low due to different production constraints, including the limited availability of improved varieties and the slow replacement rate of old varieties with new and high-yielding ones. Ensuring high genetic diversity in the working germplasm is among the primary factors for the success of breeding programs in identifying high-yielding and well-adapted improved varieties. This study aimed to assess the genetic diversity and population structure of 45 soybean breeding lines of the International Institute of Tropical Agriculture soybean breeding program at the advanced evaluation stage based on

phenotypic traits and SNP markers to support breeding strategies. Field trials were conducted in 2022 across three International Institute of Tropical Agriculture stations in Nigeria using a 5 × 9 alpha-lattice design with three replications. The collected yield and yield component data were subjected to analysis of variance, mean comparison, principal component analyses, and cluster analyses using R software. The genotypes were further assessed for SNP-based genotypic diversity analyses. The combined analysis of variance revealed a significant genotype x location interaction for grain yield and a highly significant difference in days to 50% flowering and days to 95% maturity. The genotypes G02, G10, G11, G01, and G24 were significantly superior in grain yield. Principal component analysis showed that the first three components explained 64.8% of total variation, while hierarchical clustering grouped genotypes into five clusters based on phenotype and three clusters using SNP data. The SNP markers showed average observed heterozygosity, expected heterozygosity, minor allele frequency, and polymorphic information content of 0.08, 0.27, 0.20, and 0.22, respectively, which showed the existence of considerable genetic variation among the studied genotypes. The current study suggested that the genotypes under investigation exhibited a high genetic variation in the studied genotypes.

Keywords: Principal component analysis, Cluster analysis, SNP marker, Minor allele frequency, Admixture

Introduction

Soybean [*Glycine max* (L.) Merrill] is one of the leading oilseed crops globally, accounting for approximately 57% of global vegetable oil production [1, 2]. It is cultivated in numerous countries and serves as a major source of vegetable oil and protein, essential in food, feed, and various industrial applications [3]. With a protein level of 40–42% and an oil content of 18–22%, soybeans

have twice as much protein as meat or chicken and all eight essential amino acids required for a child's healthy development [4]. Soybeans were domesticated around the 11th century BC in Northeast China and subsequently spread across Asia, the USA, Brazil, and Argentina [5]. Nigeria ranks as the second-largest producer of soybeans in Africa, following South Africa [6]. Soybeans were first introduced to Nigeria in 1908, with successful commercial cultivation beginning in 1937, using the Malayan variety in Benue State [7]. The crop is adapted to diverse environmental conditions and is predominantly grown under rain-fed conditions [8]. Despite Nigeria being the second highest producer of soybeans in Africa, the national average yield is <1 ton/ha, which is far below the potential yield of the crop (over 3 tons/ha) [6]. However, the limited availability of high-yielding and disease-resistant varieties and the slow rate of replacement of old varieties with high-yielding and climate and stress-resilient varieties are among the major factors contributing to the low yields of soybeans. Hence, enhancing the genetic improvement of the crop to make high-yielding climate-resilient varieties available for production can have paramount importance in increasing soybean production, which will greatly benefit humanity, primarily by reducing malnutrition. Genetic improvement of soybeans plays a pivotal role in addressing malnutrition in Nigeria through enhancing the crop's nutritional composition [9]. Currently, efforts are underway by the IITA soybean breeding program to improve protein content, fatty acid profiles, and reduce anti-nutritional factors such as phytic acid and protease inhibitors, thereby increasing the crop's nutritional value and its role in combating malnutrition. Recent studies have shown that local soybean varieties differ significantly in their proximate composition, including protein and fat levels, highlighting the potential for selecting superior nutritional lines [10, 11]. Additionally, integrating biofortification strategies into soybean breeding can help enhance the supply of essential micronutrients like iron and zinc, which are commonly deficient in low-income

populations [12, 13]. Thus, soybean improvement strengthens food security and serves as a tool for mitigating micronutrient deficiencies and protein-energy malnutrition [14, 15].

A fundamental step for success in any breeding program is evaluating and understanding the extent of genetic variability in the crop of interest [16]. The genetic diversity of a crop species can be assessed using phenotypic traits and molecular markers [17]. A phenotypic diversity study is the standard method of evaluating the extent of genetic diversity and determining the agronomic value and grouping of crop germplasm [18]. Understanding the phenotypic variation and trait relationships helps crop breeders to develop more adaptable and productive varieties [17]. Key traits, like number of seeds per plant/pod, number of pods per plant, 100 seeds weight, leaflet shape, flower color, stem architecture, number of days till flowering or maturity, plant height, pubescence type, and density, grain yield and other related factors are among the phenotypic qualities that are typically evaluated for genetic diversity of soybean [19-25]. Several studies on phenotypic traits have found a high genetic diversity in soybean germplasms. Liu et al. [26] reported high phenotypic variation in characterizing 138 soybean accessions based on yield and yield-related agro-morphological traits. Similarly, Bairagi et al. [27] reported high genetic variation among 32 soybean genotypes based on ten morphological traits, while Kuswantoro et al. [28] studied the phenotypic diversity of 100 soybean genotypes and reported significant variations for all the agronomic traits. Based on nine agro-morphological traits, Marconato et al. [29] also reported the existence of high genetic diversity among the 93 soybean accessions maintained by the Brazilian Agricultural Research Corporation (EMBRAPA) gene bank. All the aforementioned findings indicate that the agro-morphological traits were helpful in assessing genetic diversity that facilitates the utilization of the genetic resources for the genetic improvement of the crop. In crop

species and their relatives, selection based on phenotypic features is still frequently used and will likely continue to be an important approach in determining the extent of diversity [25].

Molecular markers are the preferred approach for assessing genetic diversity due to their excellent repeatability, superior genome coverage, automation potential, great variability, neutrality, and lack of sensitivity to environmental variations [30]. There are reports on using different types of molecular markers for diversity and population structure studies in soybeans. However, SNP markers are the most commonly used molecular markers in genetic diversity in most of the recent studies, given that they are extensively spread across the plant's genome [31]. This is due to its affordability, target accuracy, and codominant character [32, 33]. Genetic diversity studies based on SNP markers were conducted on various crops including soybeans [34-37] cassava [33]; maize [38-40]; and yam [41-43]. Despite, the great significance of assessing the phenotypic and genotypic diversity, the study materials that were developed by the IITA soybean breeding program and were at an advanced stage of evaluation have not been assessed for their genetic diversity based on both the phenotypic traits and molecular markers. Therefore, the objectives of this study were to evaluate the genetic diversity and population structure of the advanced soybean breeding lines of the IITA soybean breeding program for yield and yield-related traits using agro-morphological traits and SNP markers to recommend the best-performing varieties for direct production or use as parental lines for future genetic improvement of the crop.

Materials and Methods

Description of the study area

The field experiments were conducted across three stations i.e., Ibadan, Zaria, and Ikenne of the International Institute of Tropical Agriculture (IITA), Nigeria in the 2022 cropping season. The study locations represent Nigeria's different soybean production environments and are characterized by different agroclimatic conditions presented in Table 1.

Table 1. Trial locations and their respective agro-climatic descriptions.

Location	Ecology	State	Long.	Lat.	Elevation (masl)	Rainfall (mm)	Temp.(°C) Min	Max
IITA, Ibadan	Derived Savannah	Oyo	7°30′N	3°54′E	243	1300-1500	22	32
Ikenne	Lowland humid forest	Ogun	6°52′N	3°43′E	235.2	1200	24	30
Zaria	Northern Guinea. Savannah	Kaduna	11°11′N	7°38′ E	600	1045	17.1	35.4

masl = meter above sea level, mm = millimeter, °C = degree Celsius Source: [44, 45].

Experimental design and management

The field trial was laid out in a 5 x 9 alpha lattice design with three replications. Each entry was planted in a plot of 4 rows of 4 m length. The spacings between rows and plants were 50 cm and 5 cm, respectively. The two middle rows were harvested to measure plot yield and other related traits, and two border rows were left to exclude the border effect. A mixture of NPK and TSP fertilizers was applied at 25g/row at a 1:2 ratio at planting. The seeds were inoculated with *Bradrhizobium japonicum* inoculant called Nodumax, manufactured by the IITA Business

Incubation Platform (BIP). All the rest of the management practices were applied as per the recommendation for the crop [46].

Experimental materials

A total of 45 soybean breeding lines, which are part of the working germplasm of the IITA soybean breeding program, along with the IITA check (TGX-1951-3F a variety developed by IITA and released in Nigeria) and a commercial check (SC-Signa, a variety released by a private company called SeedCo in Nigeria) were used in the study. Among the entries, 38 genotypes were developed by and sourced from the soybean breeding program of IITA that were at an advanced stage of yield trials in Nigeria; four from the USDA soybean genetic resource center, one from Ghana, and one from Uganda. The study genotypes with their corresponding sources are presented in Table 2.

Table 2. The pedigrees, code, and source of 45 soybean genotypes were used in the study.

No	Pedigree	Genotype Code	SNP Designation	Source
1	TGx1951-4F	G01	SY001	IITA
2	TGx1993-18FN	G02	SY002	IITA
3	TGx2015-2E	G03	SY003	IITA
4	TGx1989-11FxTGx1987-10F-1-1-3-1-3-I	G04	SY004	IITA
5	TGx1961-1FxH10-2-3-7-2-1	G05	SY005	IITA
6	TGx1961-1FxTGx1835-10E-1-1-4-2-1-E	G06	SY006	IITA
7	TGx1987-9FxTGx1835-10E-1-2-2-1-1	G07	SY007	IITA
8	TGx1987-11FxH7-3-1-1-1-2-3-I	G08	SY008	IITA
9	TGx1987-11FxH7-3-1-1-1-2-6-I	G09	SY009	IITA
10	TGx1989-19FxTGx1987-10F-5-3-1-2-2-I	G10	SY010	IITA
11	TGx1989-45FxTGx1835-10E-3-2-1-3-3-E	G11	SY011	IITA
12	TGx2022-3E	G12	SY012	IITA
13	TGx1987-62FxH7-1-1-4-3-2-E	G13	SY013	IITA
14	TGx1990-38FxTGx1835-10E-1-4-3-1-1-E	G14	SY014	IITA
15	TGx2029-21F	G15	SY015	IITA
16	Panaroma-3	G16	SY016	USDA
17	TGx2029-7F	G17	SY017	IITA
18	Panaroma-1	G18	SY018	USDA
19	TGx2029-31F	G19	SY019	IITA
20	TGx2029-49F	G20	SY020	IITA
21	Panorama-27D	G21	SY021	USDA
22	TGx2029-53F	G22	SY022	IITA
23	TGx2014-16FM	G23	SY023	IITA
24	Sc-Signa	G24	SY024	Seedco
25	Songda	G25	SY025	SARI, Ghana
26	TGx2029-39F	G26	SY026	USDA
27	TGx1951-3F	G27	SY027	IITA-Check
28	(TGx1987-9F/TGx1740-2F)-#F5-1025-1	G28	SY028	IITA
29	(TGx1740-2F/MW1)-#F6-2002-10-15	G29	SY029	IITA
30	(TGx1740-2F/MW1)-#F6-2002-10-19	G30	SY030	IITA
31	(TGx1740-2F/MW1)-#F6-2002-10-28	G31	SY031	IITA
32	(TGx1830-20E/UG5)-#F6-2014-27-5	G32	SY032	IITA
33	(TGx1830-20E/UG5)-#F6-2014-27-6	G33	SY033	IITA
34	TGx2029-22F	G34	SY034	IITA
35	TGx1448-2E	G35	SY035	IITA-check
36	TGx1989-19F	G36	SY036	IITA-check
37	(TGx1740-2F/MW1)-#F6-2002-10-11	G37	SY037	IITA
38	TGx2029-20F	G38	SY038	IITA
39	TGx2029-27F	G39	SY039	IITA
40	(TGx1987-10F/TGx1740-2F)-#F5-1011-6	G40	SY040	IITA
41	(TGx1987-10F/TGx1740-2F)-#F5-1011-8	G41	SY041	IITA
42	(TGx1987-62F/MW1)-#F5-1006-15	G42	SY042	IITA
43	(TGx1987-62F/MW1)-#F5-1006-22	G43	SY043	IITA
44	Maksoy-4N	G44	SY044	Uganda
45	(TGx1740-2F/MW1)-#F6-2002-10-21	G45	SY045	IITA

Phenotypic data collection

The agro-morphological traits of plant height and root nodule score were determined from the average values of five randomly selected plants of each genotype, whereas days to 50% flowering, days to 95% maturity, lodging score, shattering score, hundred seed weight and grain yield were collected on a plot basis from the trials following the soybean descriptor of IBPGR [47] as shown in Table 3.

Table 3. List of traits and their measurements.

SN	Traits	Code	Measurement
1	Plant height (cm)	PH	measured in cm using a centimeter ruler as the height of the plant from the base to the tip of the plant at maturity
2	Days to 50% flowering (days)	D50F	The number of days taken from sowing to 50% of the plant bears at least one flower
3	Days to 95% maturity (days)	D95M	Recorded when at least 95% of the pods attained a mature brown color.
4	Lodging (score)	Lodg	All plants in the middle two rows were scored using a scale of 1 to 5; where 1=all plants erect, 2=slight lodging, 3=plants lodged at a 45-degree angle, 4= severe lodging, and 5=all plants flat.
5	Root Nodule (score)	RtNd	Plants from the border rows were uprooted and visually scored using a scale of 1 to 5; where 1=no nodules, 2= with few nodules, 3 = half of the roots have nodules, 4 = more than half of the roots have nodules, and 5=all roots have nodules
	Shattering (score)	SHS	was scored from the border rows, one week after harvesting the middle rows, using a scale of 1 to 5; where 1=very tolerant 2=tolerant, 3=moderately tolerant, 4=highly susceptible, and 5=very highly susceptible
7	Hundred seed weight (g)	HSW	The weight of a hundred seeds selected at random from each of the genotypes was recorded and expressed in grams

8	Grain yield (kg)	GY	Seeds from plants harvested from the middle two rows and contributing to the yield sample in the net plot were subjected to uniform drying conditions, weighed in grams, and converted to kilograms per hectare.

Phenotypic data analysis

Analysis of variance and mean comparisons

The quantitative data were subjected to a combined analysis of variance to test for significant differences among genotypes using the linear mixed model (LMER) procedure of the R software package (version 4.3.1, 2023). The mean comparisons were done using the least significant differences (LSD) at a 5% level of significance.

Cluster analysis

Cluster analyses were used to group the genotypes into homogeneous forms based on quantitative characters. Hierarchical clustering was conducted using the Euclidean distance and Ward's D^2 method, based on similarity coefficients among the 45 soybean genotypes. The analysis was performed in R software (version 4.3.1, R Core Team, 2023) utilizing the 'dendextend' package.

Principal components analysis (PCA)

Principal component analysis (PCA) was computed to determine the traits that accounted for much of the total variation and to assess the extent of genetic diversity in the studied genotypes. The analysis was performed using ‘FactoMiner’ and ‘factoextra’ libraries of R software [48]. In this analysis, only principal components (PCs) with eigenvalues greater than one were considered important for the total variations.

Genotyping

Leaf sampling, DNA extraction, and genotyping data processing

Seeds of the 45 genotypes were sown in the screen house at IITA, Ibadan, for sampling. For the analysis, immature, healthy trifoliate leaves from three-week-old seedlings were collected from four to five plants of each of the 45 genotypes (Table 2) using a leaf puncher and kept in a zip-lock bag on ice and later stored at −80 ◦C in a deep freezer dryer. Before genomic deoxyribonucleic acid (DNA) extraction, each sample leaf was bulked and lyophilized for 72 hours in a Labconco Freezone 2.5 L System lyophilizer (Marshall Scientific, LABCONCO, Kansas, MO, USA) and reduced to a fine powder in the SpexTM Sample Prep 2010 Geno/Grinder (Thomas Scientific, Metuchen, NJ, USA). The deoxyribonucleic acid (DNA) was extracted using a technique developed by Intertek-AgriTech (http://www.intertech.com/agriculture/agritech/), accessed 16 January 2024, and based on the LGC oKtopure^{TM} automated high-throughput 'sbeadex^{TM}' DNA extraction and purification system (https://www.biosearchtech.com/), accessed 16 January 2024.

Magnetic separation was used in the 'sbeadex^{TM}' technique to prepare nucleic acids. The first stage in this process was to homogenize leaf tissue samples in 96 deep-well plates using steel bead grinding. The ground tissue was treated with a DNA extraction buffer using LGC's 'sbeadex^{TM}' kit for plant DNA preparation (https://www.biosearchtech.com/, accessed 16 January 2024). Finally, super-paramagnetic particles coated with 'sbeadexTM' surface chemistry that catches nucleic acids from a sample were used to purify extracted DNA. Purified DNA was eluted and used in downstream procedures. Medium-throughput genotyping was conducted in a 96 plex DArTseq protocol, and SNPs were called using the DArT's proprietary software, DArTSoft, as described by

Kilian et al. [49]. Each sequencing result's reads and tags were mapped to the G. max reference genome, which was used to convert the raw HapMap file to a Variant Call Format (VCF).

Genotypic data analysis

Using the software PLINK 1.9 and VCF tools, DArTseq SNP-derived markers were filtered to remove low-quality SNP markers. A total of 59,126 SNP markers were identified from the raw data before quality assessment. The SNP markers with minor allele frequencies <0.01, markers with >20% missing data, unmapped markers to any chromosome, and duplicated markers were removed. In the end, 10,630 informative SNP markers were retained and used for the subsequent analysis. Diversity indices statistics, such as observed heterozygosity (Ho), expected heterozygosity (He), minor allele frequency (MAF),) and the polymorphic information content (PIC) was estimated using VCFtools [50] and PLINK 1.9 [51]. Ho was calculated with the method suggested by Chesnokov and Artemyeva [52]

$$\mathrm{Ho} = \frac{\text{Total number of heterozygote individuals}}{\text{Sample size}} \qquad \text{Equation 1}$$

He was determined following the equations given by Liu [53]

$$\mathrm{He} = 1 - \sum \mathrm{pi}^2 \qquad \text{Equation 2}$$

Where the summing is over all possible alleles, and **pi** is the frequency of the ith allele

The MAF values were calculated using Xue et al. [54] equation as follows

$$\mathrm{MAF} = \frac{x_i}{x} \qquad \text{Equation 3}$$

where Xi is the number of minor alleles detected at a point, and X is the total number of genotypes detected at a point

In the same way, the PIC values were calculated by the following formula: Amiryousefi et al. [55]

$$PIC = 1 - \sum pi^2 - \sum\sum pi^2 pj^2 \quad \text{Equation 4}$$

Where the first summation reflects the entire number of alleles (i^{th} and j^{th} alleles have population frequencies of pi and pj, respectively), whereas the two subsequent summations represent all i and j, where $i \neq j$.

Bayesian information criterion (BIC) was used to estimate the optimum sub-populations (K) using discriminant analysis of principal components (DAPC) and which was implemented in R using the 'adegenet' package [56]. Using the ancestry probability, the level of admixture was estimated, and individuals were assigned to a specific population when their membership coefficient in that group was ≥0.70. Genotypes with membership coefficients less than 0.70 at each assigned K were considered admixed. Coefficients of similarity showing genetic distances among the soybean lines were calculated in R software following Gower's Distance model [57]

Results

Combined analysis of variance for agronomic traits over locations

The results from the combined analysis of variance (ANOVA) across three locations are presented in Table 4. The combined ANOVA revealed a significant ($p \leq 0.05$) genotype x location interaction for grain yield and a highly significant ($P<0.001$) difference for days to 95% maturity and days to 50% flowering. However, a non-significant genotype x location interaction was observed for shattering, root nodules, lodging, plant height, and hundred seed weight. The genotype effect was highly significant ($p \leq 0.001$) for all the traits except for hundred seed weight. The location effect

also showed a significant effect (P <0.05) for most traits, except for lodging, days to 50% flowering, and hundred seed weight**.**

Table 4. Mean squares for eight agronomic and yield traits of 45 soybean genotypes evaluated in three agroecologies of Nigeria

SOV	Genotype (G)	Location(L)	GxL
DF	44	2	88
SHS	1.13***	4.25*	0.39ns
RtNd	1.14***	71.3***	0.68ns
Lodg	1.2***	0.22ns	0.41ns
PH	839***	1010***	138ns
D50F	42.7***	27.7ns	34.9***
D95M	37.3***	376***	88***
HSW	93ns	126ns	76.2ns
GY	846000***	7900000***	501000*

*, *** Significant difference at p<0.05 and p<0.001, ns = non-significant, SOV = source of variation, DF = degree of freedom, GxL = genotype by location interaction, SHS = shattering score, RtNd = root nodule score, Lodg = lodging score, PH = plant height in centimetre, D50F=days to 50% flowering, D95M = days to 95% maturity, HSW = hundred seed weight in gram, GY = grain yield in kilogram per hectare.

Mean performances of the soybean genotypes for yield and yield-related traits

The mean comparisons of agronomic and yield-related traits that were determined during the study are presented in **S3** Table. High mean yields of 3310, 3210, 3060, 3050, and 2990 kg/ha were recorded for genotypes G02, G10, G11, G01, and G24, respectively. The lowest-yielding genotypes were G35 (1700 kg/ha), G15 (2020 kg/ha), G18 (2040 kg/ha), and G28 (2140 kg/ha). Days to 95% maturity varied from 112 to 121 days with G32 and G33 maturing earlier (112 days)

than the rest, while G25 was relatively late maturing (121 days). Mean days to maturity value of 117 days were recorded for genotypes G02, G26, G36, G39, G40 and G45. Days to 50% flowering varied from 44 to 55 days with the earliest genotype being G23 (44 days) followed by G33 with 45 days and G24 and G39 with 46 days. The genotypes that took longer to flower were G04, G05, G08, and G13 all with 55 days. Shattering ranged from 0.96 to 3.5 with an average of 1.3. Genotype G45 had a higher shattering score of 3.5. Lower shattering scores were recorded by G43 (0.96), G27, G40, and G07 (0.97). The maximum lodging score was recorded by G44 (2.43), G01 (2.4), and G08 (2.3), while the minimum was observed in G22 (1), G20 (1.02), and G17 (1.06). The root nodule score ranged from 2.17 to 3.61 with an average of 3.13. Among the 45 soybean genotypes, 27 genotypes nodulated more efficiently (above average) and the best were genotypes G02, G16 (3.61), G24, G29, G45 (3.56), and G01, G07, G15 (3.50). Genotypes G17 (102 cm), G20 (98.6 cm), G16 (96.4 cm), G19 (95.2 cm) and G26 (94.3cm) were found to be the tallest. Genotypes G02 (63.6 cm), G03 (64.8 cm), G31 (67.4 cm), and G12 (68.3 cm) were the shortest genotypes in this study.

Principal component analysis (PCA)

The first three principal components with eigenvalues greater than one contributed to 64.8% of the total variation among the genotypes (Table 5, **S1** Table). The first principal component (PC1) accounted for 27.2% of the total variation. The lodging score showed a high and positive association, while days to 95% maturity had a negative association with PC1. The considerable variation observed in the second principal component (21.3%) was positively and highly correlated with hundred seed weight and plant height, and negatively correlated with days to flowering. The third principal component accounted for 16.3% of the total variation and had a high positive correlation with nodulation and days to 50% flowering. The biplot based on PC1 and PC2 that

captured 27.2 and 21.3% of the total variation, respectively, with a cumulative contribution of 48.7% (Fig 1) displayed that genotypes G15, G24, G45, G13, and G02 were far from the origin, whereas G23, G43, G11, G07, G01, and G09 were located close to the origin.

Fig 1. Distribution of genotypes and traits in PCA-Biplot of the soybean genotypes evaluated for eight agronomic and yield-related traits. Where SHS = shattering score, RtNd = root nodule score, Lodg = lodging score, PH = plant height (cm), D50F = days to 50% flowering, D95M = days to 95% maturity, HSW = hundred seed weight (gm), GY = grain yield (kg/ha).

Table 5. Eigenvalues, percent of the variance, and cumulative variance of the first three PCs of the studied soybean genotypes for eight agronomic and yield traits.

Traits	PCs		
	I	II	III
	Eigenvectors		
D50F	-0.13	-0.53	0.51
RtNd	-0.25	0.27	0.65
Lodg	0.42	-0.38	0.21
SHS	0.26	0.23	0.38
PH	-0.39	0.42	0.04
D95M	-0.53	-0.11	0.17
HSW	0.33	0.51	0.12
GY	0.38	0.10	0.29
Eigenvalues	2.17	1.71	1.30
Partial variation (%)	27.2	21.3	16.3
Cumul. variation (%)	27.2	48.5	64.8

PCs = Principal components, SHS = shattering score, RtNd = root nodule score, Lodg = lodging score, PH = plant height (cm), D50F = days to 50% flowering, D95M = days to 95% maturity, HSW = hundred seed weight (gm), GY = grain yield (kg/ha).

Cluster analysis

The cluster analysis resulting from the hierarchical ascending classification (HAC) grouped the soybean genotypes into five cluster groups, as shown in Table 6 and Fig 2.

Fig 2. Dendrogram of the studied soybean genotypes evaluated across three locations in Nigeria.

Cluster I had the highest number of genotypes (12); while cluster IV had the lowest number of four genotypes. Genotypes in cluster I were high-yielding, relatively the highest lodged, had the highest number of nodules, and the latest flowering. The genotypes in cluster II were also high yielding, earliest flowered and matured, short height, and lowest nodulation. Cluster III consisted of late flowering and maturing, low yielding, low hundred seed weight, and shattering tolerant genotypes. In contrast, genotypes in cluster IV had the highest yield, heaviest hundred seed weight, and the highest number of nodulations, and were moderately tolerant to shattering. Cluster V is composed of tall and late-maturing genotypes with the lowest values for lodging.

Table 6. Cluster mean values of the studied agro-morphological traits of the soybean genotypes and their clustering.

	Clusters				
Traits	I (12)	II (8)	III (10)	IV (4)	V (11)
D50F	52	47	53	48	47
D95M	112	109	115	112	116
GY (kg/ha)	2695.49	2688.74	2319.34	2786.85	2357.05
HSW (g)	15.07	15.31	12.99	16.18	14.00

Lodg	2.05	1.89	1.76	1.49	1.23
PH (cm)	81.35	70.92	73.09	76.73	92.10
RtNd	3.33	2.67	3.06	3.33	3.24
SHS	1.25	1.22	1.16	2.36	1.17

NB: Numbers in parenthesis are the number of genotypes in that cluster, D50F = days to 50% flowering, D95M = days to 95% maturity, GY = grain yield in kilogram per hectare, HSW = hundred seed weight in gram, Lodg = lodging score, PH = plant height in centimeter, SHS = shattering score, RtNd = root nodule score.

Genetic diversity of soybean displayed by SNP markers

The results of the SNP markers' statistics are summarized in Table 7 and **S1** Fig. The observed heterozygosity ranged from 0.00 to 0.16, with a mean of 0.08, while the expected heterozygosity (He) ranged between 0.00 and 0.50, with a mean of 0.27. The minor allele frequency (MAF) ranged between 0.01 and 0.50 with a mean value of 0.20 and the polymorphic information content (PIC) ranged from 0.00 to 0.38 with an average value of 0.22.

Table 7. Diversity indices statistics of the 45 soybean genotypes based on 10,630 SNP markers.

	Ho	He	MAF	PIC
Minimum	0.00	0.00	0.01	0.00
Maximum	0.16	0.50	0.50	0.38
Mean	0.08	0.27	0.20	0.22

Ho = observed heterozygosity, He = expected heterozygosity, MAF=minor allele frequency, PIC=polymorphic information content

The population structure analysis in this study identified three sub-populations among the 45 soybean genotypes based on the optimal K=3 determined based on the BIC method (Fig 3)**.**

Fig 3. Graph showing the number of clusters vs the Bayesian Information Criterion (BIC).

Using the 70% cut-off criterion of the membership probability threshold, 27 genotypes were successfully assigned to the three different sub-populations. The remaining 18 genotypes with a probability of associations of less than 70% were considered as an admixed population (Fig 4, **S2** Table).

Fig 4. Population structure of the studied soybean genotypes based on the SNP markers. Red, blue, and green color denotes sub-populations I, II, and III, respectively.

Sub-population I (red) was comprised of 13 genotypes that were sourced from IITA and Uganda, while the seven genotypes were allocated in sub-population II (blue) that were sourced from Ghana and IITA. Sub-population III (green) consisted of seven genotypes obtained from USDA and IITA. The phylogenetic tree also showed three sub-populations with higher degrees of admixture similar to the BIC (Fig 5).

Fig 5. The phylogenetic trees of the studied soybean genotypes based on the SNP markers. Dots indicating individual genotypes.

Similarly, the DAPC assigned the genotypes to three cluster groups and clearly showed a higher degree of admixture among the genotypes. The first and second PCs accounted for 35.8 and 13.8% of the total variation, respectively. The members of Cluster III were the most compact in distribution, while those of Cluster I were the most widely distributed along with the axes of the first two PCs (Fig 6).

Fig 6. Scatter plot based on the first two PCs obtained from DAPC based on the SNP markers displaying the distribution of the studied genotypes. Note Each color corresponds to population structuring and grouping.

Genetic distance between genotypes based on 10,630 SNP markers

The genetic distances across the genotypes are summarized in **S4** Table. In cluster one, the highest genetic distance (0.335) was observed between genotypes SY007 and SY024, while the lowest genetic distance (0.14) was observed between genotypes SY013 and SY012. In cluster two, the maximum genetic distance (0.335) was observed between genotypes SY026 and SY030, while the minimum genetic distance (0.012) was observed between genotypes SY037 and SY029. In cluster three, the widest genetic distance (0.335) was observed between genotypes SY030 and SY026, while the shortest genetic distance (0.014) was observed between genotypes SY026 and SY020.

Discussion

Genetic diversity analysis in crops is required for the success of any plant breeding effort [58]. Hence, evaluating crops' population structure and genetic diversity is vital for adopting effective genetic resource management and conservation measures [59]. The genotype x location interactions across the three locations were significant for grain yield, and highly significant for days to 95% maturity, and days to 50% flowering, implying genotypes exhibited varying relative performances across locations for these traits. The results are in line with the findings of Tesfaye et al. [60], Krisnawati and Adie [61], and Pedro [62], who reported a highly significant genotype x environment interaction for days to 50% flowering, days to 95% maturity, and grain yield. Njoroge et al. [63], Nachilima [64], and Abebe et al. [65] also reported significant genotype x environment interaction for grain yield. Five genotypes i.e., G02, G10, G11, G01, and G24 showed significantly superior performance for grain yield (Table 5). These genotypes can be released as varieties for direct production by farmers after further evaluation or can be used as parents in the soybean hybridization programs. Getnet [66], Beyene et al. [67], and Thio et al. [68] also obtained different mean yield performances among soybean genotypes in their studies.

Days to 95% maturity varied from 112 to 121 days, with G32 and G33 maturing earlier than the rest, while G25 maturing relatively late. Beyene et al. [67], Thio et al. [68], Sileshi [69], Goonde and Ayana [70] and Yirga et al. [71] reported comparable trends in variability in days to maturity for different soybean genotypes. Days to 50% flowering varied from 44 to 55 days with the earliest genotype being G23, followed by G33. The genotypes that took longer to flower were G04, G05, G08, and G13 all with 55 days. The results for days to 50% flowering agree with the findings of Goonde and Ayana [70] and Jandong et al. [72], and Akter [73]. Shattering ranged from 0.96 to 3.5 with the highest tolerant genotypes being G43, G07, and G27, whereas G45 was highly susceptible to pod-shattering. This result aligns with Fatima et al. [45] and Aondover et al. [74], who reported different mean performances in pod shattering. G44 recorded the maximum lodging score, while the minimum was observed in G22. This aligns with the work of Antwi-Boasiako [75], who found significant variation in tolerance to lodging and shattering across the 34 soybean genotypes evaluated. The root nodule score ranged from 2.17 to 3.61 with an average of 3.13. Among the 45 soybean genotypes, 27 nodulated more efficiently, and the best genotypes were G02, G16, G24, G29, G45, G01, G07, and G15. These results agree with those of Thio et al. [68] and Bello et al. [76], who reported different mean performances in root nodule scores. The plant height among the genotypes varied from 63.6 to 102 cm. The tallest plant height was recorded for G17, whereas the shortest was recorded on G02, which was also the top-performing genotype for grain yield. The results of this study is in line with the findings of Thio et al. [68] and Njoroge and Njeru [77], who reported plant height ranged from 51.1 cm to 102.8 cm and 60 cm to 109cm, respectively. In contrast, Hizli et al. [78] reported up to 151.8cm plant height, which is taller than the heights found in this study.

Principal component analysis (PCA) aids in simplifying multidimensional data by breaking down its complexity into simpler principal components (PCs) by retaining important information [79, 80]. The first three PCs with eigenvalues greater than one, which accounted for about 64.8% of the total variation were considered important (Table 5). In a similar study on soybeans, Aondover et al. [74] reported that the first three principal components accounted for 76.4% of the total variation. Similarly, Verma et al. [81] reported that the first three PCs contributed 91.7% of the total variation. In their study, Akter [73] and Vijayakumar et al. [80] also reported that the first three PCs accounted for 77.7% and 73.7% of the total variation, respectively. Each PC is independently associated with the different yield and yield-related variables examined. The first PC that showed the highest contributions (27.2%) to the total variation was highly and positively associated with lodging and highly and negatively associated with days to 95% maturity, indicating the importance of these traits in the genetic improvement of the studied germplasm. The average contributions of RtNd, HSW, PH, D50F, and Lodg were high in the principal axes. This finding implies that these traits account for the majority of the variations in soybeans and further influence the yield of soybeans. This finding aligns with the results of Aondover et al. [74], Adetiloye et al. [82], Sivabharathi et al. [83], Vianna et al. [84], and Leite et al. [85], who reported the mean contributions of RtNd, HSW, PH, D50F, and Lodg were high in the principal axes. Thus, these traits must be considered when selecting grain yield. Soybean genotypes were spread over the biplot based on the first two PCs, also reflecting high variability in the studied germplasm. Genotypes G15, G24, G45, G13, and G02 were far from the origin and identified as highly interactive and diverse genotypes; whereas G23, G43, G11, G07, G01, and G09 were located close to the origin and considered stable genotypes or less diverse genotypes (Fig 1). Comparable results were reported by Sivabharathi et al. [83], who performed principal component analysis on 135

soybean genotypes and reported genotypes MACS 1460, EC 18736, and PK 1038 as highly divergent genotypes, while JS 89-24, NRC 25, NRC 2007-G-1-13, NRC 43 and PK 7247 genotypes were less diverse and stable.

The studied genotypes were grouped into five distinct clusters, which also indicates the divergent nature of these germplasm. This result was comparable to the findings of Getnet [66] and Ghiday and Sentayehu [86], who reported the clustering of 49 soybean genotypes into five and three distinct clusters, respectively. Similarly, Yirga et al.[71] performed cluster analysis of 100 soybean genotypes based on morphological traits that were grouped into five different clusters, which implied the presence of genetic variability among the tested genotypes. Adetiloye et al. [82] also reported the clustering of 43 accessions into nine distinct clusters. These findings indicate the studied soybean genotypes exhibited wide genetic diversity. Utilizing genetically diverse parents in hybridization programs is critical in crop improvement activities [73] in ensuring high genetic recombinations in the progeny populations. Considering the cluster mean (Table 6), the genotypes in cluster IV showed the highest performances for grain yield and 100 seed weight, while the genotypes included in cluster II might be the preferred cluster to improve the crop for early flowering and maturity, and cluster I for high nodulation. The present outcome supports prior findings [87-90].

In this study, the observed heterozygosity (Ho) of 0.08 was lower than the expected heterozygosity of the genotypes, possibly due to soybeans' high self-pollinating nature [91]. This implies high possibilities of inbreeding and fixation at most loci [92, 93]. The mean observed heterozygosity of 0.08 obtained in this study is higher compared to the 0.02 and 0.06 reported by Tsindi et al. [35] and Chiemeke et al. [94], respectively, in soybeans using SNP markers. However, it is lower compared to the 0.22 stated by Yohane et al. [95] when assessing 81 pigeon pea genotypes based

on 4122 SNP markers, but similar to the results of Gumede et al. [59], when assessing 90 cowpea accessions using 5864 SNP markers. The average expected heterozygosity (He = 0.27) found in the current study was lower than the He of 0.31 reported by Abebe et al. [34] and Tsindi et al. [35] evaluating 65 and 210 soybean genotypes using 1223 and 403 SNP markers, respectively. The minor allele frequency (MAF) values measure the ability of markers to discriminate among genotypes [35]. Minor allele frequency (MAF) varied from 0.01 to 0.50 with an average of 0.20. The average MAF value found in this study was greater than the 0.24 reported by Tsindi et al. [35] based on the 403 SNPs in 210 soybean genotypes, 0.25 reported by Naflath et al. [96] based on the 29,955 SNPs in 96 soybean genotypes and 0.32 reported by Chander et al. [97] based on 186 SNPs in 155 soybean lines. Dube et al. [40] found similar results in maize with the MAF ranging from 0.01 to 0.5. A mean PIC value of 0.22 indicates that the markers were informative. A mean PIC value of 0.22 in the current study was lower than the PIC values reported in various crops such as 0.25 in soybean [34], 0.31 in maize [40], 0.36 in wing yam [41], 0.29 in wheat [98], 0.27 in cowpea [59], 0.26 in pea [99]. However, it is comparable to the results in other legumes i.e., 0.22 in common bean [100] and 0.23 in cowpea [101].

The observed (Ho = 0.08) and expected (He = 0.27) heterozygosity values in this study reflect soybean's self-pollinating nature, which naturally results in lower heterozygosity [91, 102]. However, the level of expected heterozygosity still indicates the presence of useful genetic variation across the population [103]. This moderate diversity is essential for breeders because it enables effective selection of genotypes with favorable traits [59, 104]. The disparity between Ho and He also suggests some degree of inbreeding and allele fixation, highlighting the need for incorporating genetically diverse parents in the crossing program to maintain and further increase the genetic base of IITA soybean [92, 97, 104]. Besides, by comparing Ho and He to other soybean

germplasm such as Abebe et al. [34], Tsindi et al. [35], and Chiemeke et al. [94], breeders can pinpoint the most diverse parents for hybridization and thus maximize recombination of useful alleles [105, 106]. Additionally, moderate average values of MAF (0.20) and PIC (0.22) confirm that the SNP markers used are informative enough to distinguish genotypes and support population structure analysis [35, 54, 97]. These diversity indices together provide valuable guidance for parental selection, germplasm conservation, and marker deployment in soybean improvement programs [54, 105].

Population structure analysis is the process of determining a breeding line's ancestry using genotypic data [38, 107]. The SNP analysis identified three sub-populations (K = 3) among the 45 soybean genotypes based on the optimal K=3 determined according to the BIC method, which was not consistent with the results of phenotypic clustering. The inconsistency might be that most of the phenotypic traits are controlled by polygenes, and environmental conditions highly influence these traits [108, 109]. In the same way, the phylogenetic tree and DAPC also showed three sub-populations with higher degrees of admixture. The high degree of admixture found suggests that there was either gene flow or that these subpopulations had a common ancestor [91]. Chander et al. [97] found comparable amounts of admixture in their study of 155 soybean genotypes, predominantly IITA-bred soybean varieties. The overall results of the population structure analysis are in agreement with the results of Abebe et al. [34] and Fatokun et al. [101], who identified three sub-populations among the 65 soybean genotypes and 298 cowpea accessions, respectively based on BIC and DAPC methods.

The analysis of genetic distances within clusters revealed considerable variation, reflecting the underlying genetic diversity among the genotypes. In cluster I, the greatest genetic distance was recorded between genotypes SY007 and SY024, suggesting substantial genetic differentiation

between these two individuals. This could imply limited recent shared ancestry or contrasting selection pressures. On the other hand, the lowest genetic distance was observed between SY013 and SY012, indicating a closer genetic relationship, possibly due to recent common ancestry or shared breeding lineage [107, 110]. In cluster II, the widest genetic distance was found between genotypes SY026 and SY030, mirroring the divergence seen in Cluster 1. Such high differentiation might suggest that these genotypes belong to distinct subpopulations or have undergone different evolutionary pressures. Conversely, the minimal genetic distance in this cluster was surprisingly low between SY037 and SY029, indicating a near-identical genetic makeup. This could reflect either clonal propagation, recent hybridization, or strong genetic conservation between the two genotypes. Cluster III also exhibited a maximum genetic distance, again between SY030 and SY026, reinforcing the notion of pronounced genetic divergence between these two genotypes across clusters. The lowest genetic distance in this cluster was between SY026 and SY020, suggesting a close relationship and potential kinship or shared origin. This pattern of high and low pairwise distances within clusters indicates that while clustering helps group genetically similar individuals, considerable diversity still exists within each group, which is critical for maintaining adaptive potential and guiding breeding decisions. Various genetic distances within soybean germplasm have been observed in a similar study by [35, 37, 102, 111]

Conclusion

The combined analysis of variance revealed that there was a significant difference among genotypes, locations, and genotype ×location interaction for days to 95% maturity and grain yield, indicating genotypes exhibited different relative performance across locations for these traits. The genotypes G02, G10, G11, G01, and G24 were found to be the most high-yielding (3050-2990 kg/ha). Principal component analysis (PCA) showed that about 64.8% of the variation was

explained by the first three principal components with eigenvalues greater than one. The average contributions of HSW, RtNd, PH, Lodg, and D50F were high in the principal axes, implying that these characters are the major contributors to the total variation and influence the yielding ability of the studied soybean genotypes. The cluster analysis resulting from the hierarchical ascending classification grouped the 45 soybean genotypes into five clusters. This suggests that there was moderate variation among the studied soybean genotypes. Based on cluster mean values, genotypes selected from distant clusters such as clusters II and IV were desirable for use as parents in future hybridization programs to develop high-yielding soybean varieties.

Concerning the markers used in the study, a total of 10,630 SNP markers were deployed to study the population structure of 45 soybean genotypes. The SNP markers were moderately informative, with a mean PIC value of 0.22 across all the genotypes. The observed and expected heterozygosity values were 0.08 and 0.27, respectively while the minor allele frequency ranged from 0.01 to 0.50 with a mean of 0.20. The Discriminant Analysis of Principal Component (DAPC) and Bayesian Information Criterion (BIC) method grouped the 45 soybean genotypes into three subpopulations. The current study suggested that the genotypes under investigation exhibited considerable genetic diversity.

Acknowledgments

The first author is grateful for the scholarship granted by the African Union Commission for her MSc studies at the Pan African University Life and Earth Sciences Institute (including Health and Agriculture), University of Ibadan, Nigeria. We would like to acknowledge the staff and technicians of the soybean breeding and biometric unit, at the International Institute of Tropical Agriculture for all their invaluable guidance.

References

[1] Berk Z. Technology of production of edible flours and protein products from soybean. *FAO Agric Serv Bull* 1992; 1–10.

[2] Yadava DK, Vasudev S, Singh N, Mohapatra T, Prabhu KV. Breeding major oil crops: Present status and future research needs. *Technol Innov major world oil Crop* 2012; Volume 1: 17–51.

[3] Wilson RF. Soybean: Market Driven Research Needs. In Genetics and Genomics of Soybean 2008; 3–15. New York, NY: Springer New York. https://doi.org/10.1007/978-0-387-72299-3_1.

[4] Malik MFA, Qureshi AS, Ashraf M, Ghafoor A. Genetic variability of the main yield related characters in soybean. *Int J Agric Biol* 2006; 8: 815-819.

[5] Hymowitz T, Shurtleff WR. Debunking soybean myths and legends in the historical and popular literature. *Crop Sci* 2005; 45: 473–476.

[6] FAOSTAT. A list of countries by soybean production from 2016 to 2020. *In Wikipedia. https://en.wikipedia.org/wiki/List_of_countries_by_soybean_production*.

[7] Akande SR, Oyekan PO and ADESOYE AI. Soybean in Nigeria: Introduction, production and utilization. *LEGUME* 2013; 16: 38.

[8] Chandrawat KS, Baige KS, Hashmi S, Sarang DH, Kumar A, Dumani PK. Study on Genetic Variability, Heritability and Genetic Advance in Soybean. *Int J Pure Appl Biosci* 2017; 5(1): 57–63.

[9] Singer WM, Lee YC, Shea Z, Vieira CC, Lee D, Li X, et al. Soybean genetics, genomics, and breeding for improving nutritional value and reducing antinutritional traits in food and feed. Plant Genome 2023; 16(4): e20415.

[10] Zewudie KG, Gemede HF. Assessment of nutritional, antinutritional, antioxidant and functional properties of different soybean varieties: implications for soy milk development. Cogent Food Agric 2024; 10(1): 2380496.

[11] Joseph OO, Uzoma AC, Juliana AN, Precious S. Proximate and Anti-Nutritional Analyses of Three Soybeans (*Glycine max* (L.) Merrill). IMCC J Sci 2024; 4(1): 10–16.

[12] Kumar A, Ramesh KV, Singh C, Sripathy KV, Agarwal DK, Pal G, et al. Bioprospecting nutraceuticals from soybean (*Glycine max*) seed coats and cotyledons. Indian J Agric Sci 2019; 89(12): 2064–2068.

[13] Tamangwa MW, Djikeng FT, Feumba RD, Sylvia VT, Loungaing VD, Womeni HM. Nutritional composition, phytochemical, and functional properties of six soybean varieties cultivated in Cameroon. Legum Sci 2023; 5(4): e210.

[14] Nwosu DJ, Olubiyi MR, Aladele SE, Apuyor B, Okere AU, Lawal AI, et al. Proximate and mineral composition of selected soybean genotypes in Nigeria. J Plant Dev 2019; 26: 67–76.

[15] Fasusi SA, Kim JM, Kang S. Current status of soybean production in Nigeria: constraints and prospects. J Korean Soc Int Agric 2022; 34: 149–156.

[16] Meena BL, Das SP, Meena SK, Kumari R, Devi AG, Devi HL. Assessment of GCV, PCV, Heritability and Genetic Advance for Yield and its Components in Field Pea (*Pisum sativum* L.). *Int J Curr Microbiol Appl Sci* 2017; 6(5): 1025–1033.

[17] Nkhoma N, Shimelis H, Laing MD, Shayanowako A, Mathew I. Assessing the genetic diversity of cowpea [*Vigna unguiculata* (L.) Walp.] germplasm collections using phenotypic traits and SNP markers. BMC genetics. 2020; 21: 1–16.

[18] Singh S, Trivedi PKA, Koshal AK. Seed Protein Profiling through Electrophoresis in Mungbean [*Vigna Radiata* (L.) Wilczek]. *J Plant Dev Sci* 2015; 7(4): 337–340.

[19] Chen Y, Nelson RL. Genetic Variation and Relationships among Cultivated Wild, and Semiwild Soybean. *Crop Sci* 2004; 44(1): 316–325.

[20] Dayaman V, Senthil N, Raveendran M, Nadarajan N, Shanmugasundaram P, Balasubramanian P. Diversity analysis in selected Indian soybean [*Glycine max* (L.) Merrill] using morphological and SSR markers. *Int J Integr Biol* 2009; 5: 125–129.

[21] Liu M, Zhang M, Jiang W, Sun G, Zhao H, Hu S. Genetic diversity of Shaanxi soybean

landraces based on agronomic traits and SSR markers. *African J Biotechnol* 2011; 10(24): 4823–4837.

[22] Malik MFA, Ashraf M, Qureshi AS, Khan MR. Investigation and comparison of some morphological traits of the soybean populations using cluster analysis. *Pakistan J Bot* 2011; 43(2): 1249–1255.

[23] Matsuo É, Sediyama T, Cruz CD, Oliveira RDL, Oliveira RCT, Nogueira APO. Genetic diversity in soybean genotypes with resistance to Heterodera glycines. *Crop Breed Appl Biotechnol* 2011; 11: 304–312.

[24] Salimi S, Lahiji HS, Abadi GM, Salimi S, Moradi S. Genetic diversity in Soybean genotypes under drought stress condition using factor analysis and cluster analysis. *World Appl Sci J* 2012; 16(4): 474–478.

[25] Mushoriwa H. Breeding Gains, Diversity Analysis and Inheritance Studies on Soybean [*Glycine max* (L .) Merrill] Germplasm in Zimbabwe. 2013; 210.

[26] Liu Z, Li H, Fan X, Huang W, Yang J, Wen Z, et al. Phenotypic characterization and genetic dissection of nine agronomic traits in Tokachi nagara and its derived cultivars in soybean (*Glycine max* (L.) Merr.). *Plant Sci* 2017; 256: 72–86.

[27] Bairagi V, Mishra S, Sen R, Dixit S, Tyagi DB. Assessment of Genetic Variability in Soybean (*Glycine max* L., Merrill). *Biol Forum–An Int J* 2023; 15: 258–263.

[28] Kuswantoro H, Adie MM, Putri PH. Genetic Variability, Heritability, and Genotypic Correlation of Soybean Agronomic Characters. *Bul Palawija* 2021; 19(2): 117–125.

[29] Marconato MB, Pereira EM, Silva FM, Bizari EH, Pinheiro JB, Mauro AO, et al. Genetic divergence in a soybean (*Glycine max*) diversity panel based on agro-morphological traits. *Genet Mol Res*.2016; 15:1-10. DOI: 10.4238/gmr15048980.

[30] Bhandari HR, Bhanu AN, Srivastava K, Singh MN, Shreya HA. Assessment of Genetic Diversity in Crop Plants - An Overview. *Adv Plants Agric Res* 2017; 7(3): 279–286.

[31] Ganal MW, Altmann T, Röder MS. SNP identification in crop plants. *Curr Opin Plant Biol* 2009; 12(2): 211–217.

[32] Ren J, Sun D, Chen L, You FM, Wang J, Peng Y, et al. Genetic diversity revealed by single nucleotide polymorphism markers in a worldwide germplasm collection of durum wheat. *Int J Mol Sci* 2013; 14: 7061–7088.

[33] Prempeh RNA, Manu-aduening JA, Quain MD, Asante IK, Offei SK, Danquah EY. Assessment of genetic diversity among cassava landraces using single nucleotide polymorphic markers. *African J Biotechnol* 2020; 19(6): 383–391.

[34] Abebe AT, Kolawole AO, Unachukwu N, Chigeza G, Tefera H, Gedil M. Assessment of diversity in tropical soybean (*Glycine max* (L.) Merr.) varieties and elite breeding lines using single nucleotide polymorphism markers. *Plant Genet Resour* 2021; 19(1): 20–28.

[35] Tsindi A, Eleblu JSY, Gasura E, Mushoriwa H, Tongoona P, Danquah EY, et al. Analysis of population structure and genetic diversity in a Southern African soybean collection based on single nucleotide polymorphism markers. *CABI Agric Biosci* 2023; 4(1): 15.

[36] Potapova NA, Zlobin AS, Perfil RN, Vasiliev GV, Salina EA, Tsepilov YA. Population Structure and Genetic Diversity of the 175 SoybeanBreeding Lines and Varieties Cultivated in West Siberia and Other Regions of Russia. *Plants* 2023;12(19):3490.

[37] Andrijani Z, Nazzicari N, Šarcevic H, Sudarić A, Annicchiarico P, Pejić I. Genetic Diversity and Population Structure of European Soybean Germplasm Revealed by Single Nucleotide Polymorphism. *Plants* 2023; 12(9): 1837.

[38] Adu GB, Badu-Apraku B, Akromah R, Garcia-Oliveira AL, Awuku FJ, Gedil M. Genetic diversity and population structure of early-maturing tropical maize inbred lines using SNP markers. *PLoS One* 2019; 14(4): https://doi.org/10.1371/journal.pone.0214810.

[39] Badu-Apraku B, Garcia-Oliveira AL, Petroli CD, Hearne S, Adewale SA, Gedil M. Genetic diversity and population structure of early and extra-early maturing maize germplasm adapted to sub-Saharan Africa. *BMC Plant Biol* 2021; 21: 1–15.

[40] Dube SP, Sibiya J, Kutu F. Genetic diversity and population structure of maize inbred lines using phenotypic traits and single nucleotide polymorphism (SNP) markers. *Sci Rep* 2023; 13(1): 17851.

[41] Agre P, Asibe F, Darkwa K, Edemodu A, Bauchet G, Asiedu R, et al. Phenotypic and

molecular assessment of genetic structure and diversity in a panel of winged yam (*Dioscorea alata*) clones and cultivars. *Sci Rep* 2019; 9(1): 18221.

[42] Bhattacharjee R, Agre P, Bauchet G, De Koeyer D, Lopez-Montes A, Kumar PL, et al. Genotyping-by-sequencing to unlock genetic diversity and population structure in white yam (*Dioscorea rotundata* Poir.). *Agronomy* 2020; 10(9):1437. doi: 10.3390/agronomy10091437.

[43] Agre PA, Dassou AG, Loko LEY, Idossou R, Dadonougbo E, Gbaguidi A, et al. Diversity of white Guinea yam (*Dioscorea rotundata* Poir.) cultivars from Benin as revealed by agro-morphological traits and SNP markers. *Plant Genet Resour* 2021; 19(5): 437–446.

[44] Adewale BD, Ojo DK, Abberton M. GGE Biplot application for adaptability of African yam bean grain yield to four agroecologies in Nigeria. African Crop Sci J 2017; 25(3): 333–347.

[45] Fatima UA, Mohammed MS, Oyekunle M, Abdulmalik MM, Usman A. Screening soybean (*Glycine Max* (L.) Merrill) genotypes for resistance to pod shattering in Zaria, Nigeria. *FUDMA J Sci* 2020; 4(1): 727–731.

[46] Omoigui LO, Kamara AY, Kamai N, Dugje IY, Ekeleme F, Kumar P, et al. Guide to Soybean Production in Northern Nigeria. *International Institute of Tropical Agriculture, Nigeria.* 2020.http://www.ajol.info/index.php/naj%0Awww.iita.org%0A

[47] International Board for Plant Genetic Resources (IBPGR). Descriptors for Soybean. 1984.

[48] R Development Core Team. R: A Language and Environment for Statistical Computing. R Foundation for Statistical Computing, Vienna, Austria, 2007. ISBN 3-900051-07-0, URL http: //www.R-project.org/.

[49] Kilian A, Sanewski G, Ko L. The application of DArTseq technology to pineapple. InXXIX *International Horticultural Congress on Horticulture: Sustaining Lives, Livelihoods and Landscapes (IHC2014): IV 1111*. 2014, pp. 181-188.

[50] Danecek P, Auton A, Abecasis G, Albers CA, Banks E, DePristo MA, et al. The variant call format and VCFtools. *Bioinformatics* 2011; 27(15): 2156–1258.

[51] Purcell S, Neale B, Todd-Brown K, Thomas L, Ferreira MA, Bender D, et al. PLINK: a tool set for whole-genome association and population-based linkage analyses. *Am J Hum Genet* 2007; 81(3): 559–575.

[52] Chesnokov YV, Artemyeva AM. Evaluation of the measure of polymorphism information of genetic diversity. Agric Biol 2015; 50(5): 571–578.

[53] Liu BH. Statistical genomics: linkage, mapping and QTL analysis. CRC Press Boca Raton, Florida, USA; 1998.

[54] Xue H, Yu X, Fu P, Liu B, Zhang S, Li J, et al. Construction of the Core Collection of *Catalpa fargesii f. duclouxii* (Huangxinzimu) Based on Molecular Markers and Phenotypic Traits. Forests 2021; 12(11): 1518.

[55] Amiryousefi A, Hyvönen J, Poczai P. iMEC: Online Marker Efficiency Calculator. Appl Plant Sci 2018; 6(6): e1159.

[56] Jombart T. adegenet: a R package for the multivariate analysis of genetic markers. *Bioinformatics* 2008; 24(11): 1403–1405.

[57] Gower JC. A general coefficient of similarity and some of its properties. Biometrics 1971; 27(4): 857–871.

[58] Ebert AW, Engels JMM. Plant biodiversity and genetic resources matter! *Plants* 2020; 9: 1706.

[59] Gumede MT, Gerrano AS, Amelework AB, Modi AT. Analysis of Genetic Diversity and Population Structure of Cowpea (*Vigna unguiculata* (L.) Walp) Genotypes Using Single Nucleotide Polymorphism Markers. *Plants* 2022; 11(24): 3480. **https://doi.org/10.3390/plants11243480**.

[60] Tesfaye A, Githiri M, Derera J, Debele T. Genetic variability in soybean (*Glycine max* L.) for low soil phosphorus tolerance. Ethiop J Agric Sci 2017; 27(2): 1–15.

[61] Krisnawati A, Adie MM. Yield stability of soybean promising lines across environments. IOP Conf Ser Earth Environ Sci 2018; 102. DOI: 10.1088/1755-1315/102/1/012044.

[62] Pedro JA. Evaluation of soybean [*Glycine max* (L.) Merrill] Genotypes for grain yield and associated agronomic traits under low and high phosphorus environments. (Master thesis,

University of KwaZulu-Natal Pietermaritzburg, South Africa. 2018.

[63] Njoroge JN, Owouche JO, Oyoo ME. Evaluation of soybean [*Glycine max* (L.) Merr.] genotypes for agronomic and quality traits in Kenya. African J Agric Res 2015; 10(12): 1474–1479.

[64] Nachilima C. Genotype plus genotype-by-environment interaction analysis of soybean [*Glycine max* (L. Merrill] across production environments in Southern Africa. (Masters thesis, The University of Zambia). 2021.

[65] Abebe AT, Adewumi AS, Adebayo MA, Shaahu A, Mushoriwa H, Alabi T, et al. Genotype x environment interaction and yield stability of soybean (*Glycine max* L.) genotypes in multi-environment trials (METs) in Nigeria. Heliyon 2024; 10: e38097.

[66] Getnet BE. Genetic Variability, Heritability and Expected Genetic Advance as Indices for Selection in Soybean [*Glycine max* (L.) Merrill] Varieties. Am J Life Sci 2018; 6(4): 52–56.

[67] Beyene D, Jalata Z, Geleta N. Exploring the Genetic Variability, Heritability and Genetic Gain in Soybean [*Glycine max* (L.) Merrill] Genotypes. Int J Agric Res 2022; 17(2): 43–49

[68] Thio IG, Ouedraogo N, Drabo I, Essem F, Neya FB, Sombie PAED, et al. Evaluation of medium maturity group of soybean [*Glycine max* (L.) Merr] for agronomic performance and adaptation in Sudanian zone of Burkina Faso. African J Agric Res 2022; 18(4*)*: 264–275.

[69] Sileshi Y. Estimation of Variability, Correlation and Path Analysis in Soybean [*Glycine max* (L.) Merr.] Genotypes at Jimma, South Western Ethiopia. J Nat Sci Res 2019; 9(7): 22–29.

[70] Goonde DB, Ayana NG. Genetic Diversity and Character Association for Yield and Yield Related Traits in Soybean (*Glycine max* L.) Genotypes. J Agric Sci Food Res 2021; 2(1): 1-5.

[71] Yirga M, Sileshi Y, Tesfaye A, Hailemariam M. Genetic Variability and Association of Traits in Soybean (*Glycine max* (L.) Genotypes in Ethiopia. Ethiop J Crop Sci 2022; 9(2):

49-74.

[72] Jandong EA, Uguru MI, Okechukwu EC. Estimates of genetic variability, heritability and genetic advance for agronomic and yield traits in soybean (*Glycine max* L.). African J Biotechnol 2020; 19(4): 201–206.

[73] Akter S. Character association and diversity analysis of different genotypes of soybean [*Glycine max* (L.) Merr.]. (Master thesis, Sher-e-Bangla Agricultural University, Dhaka-1207, Bangladesh). 2021.

[74] Aondover S, Lekan BL, Terkimbi V. Correlation, path coefficient and principal component analysis of seed yield in soybean genotypes. Int J Adv Res 2013; 1(7): 1–5.

[75] Antwi-boasiako A. Screening of soybean [*Glycine max* (L.) Merrill] genotypes for resistance to lodging and pod shattering. Int J Agron Agric Res 2017; 10(5): 1–8.

[76] Bello LL, Shaahu A, Vange T. Studies on relationship between seed yield and yield components in Soybean [*Glycine ma*x (L.) Merrill]. *Electron J Plant Breed* 2012; 3(4): 1012–1017.

[77] Njoroge JN, Njeru PNM. Performance of soybean genotypes evaluated for yield and protein content in Nakuru County, Kenya. Joint Proceedings of the 27th Soil Science Society of East Africa and the 6th African Soil Science Society Conference. 2013: 255–261.

[78] Hizli H, Cubukcu P, Sahar AK. Path Analysis, Genetic Variability and Correlation Studies of Related Characters for Forage Soybean [*Glycine max* (L.) Merrill]. O*smaniye Korkut Ata Univ Inst J Sci Technol* 2023; 6(2): 1513–1528.

[79] Jollife IT, Cadima J. Principal component analysis: A review and recent developments. *Philos Trans R Soc A Math Phys Eng Sci* 2016; 374(2065). DOI: https://doi.org/10.1098/rsta.2015.0202.

[80] Vijayakumar E, Sudhagar R, Vanniarajan C, Ramalingam J, Allan V, Senthil N. Estimating the Breeding Potency of a Soybean Core Set. *Int J Agric Biol* 2022; 27(3): 184–196.

[81] Verma V, Shrivastava MK, Mehra S, Amrate PK, Yadav RB. Estimation of Genetic Parameters for Yield Associated Traits and Principal Component in Advance Breeding Lines of Soybean [*Glycine max*. (L.) Merrill]. *Int J Curr Microbiol Appl Sci* 2021; 10(1): 2704–2710.

[82] Adetiloye IS, Ariyo OJ, Alamu O, Osewa SO. Agronomic Potential and Genetic Diversity of 43 Accession of Tropical Soybean [*Glycine max* (L) Merrill]. *Int J Plant Res* 2020; 10: 33–39. doi**: 10.5923/j.plant.20201002.02** .

[83] Sivabharathi RC, Muthuswamy A, Anandhi K, Karthiba L. Genetic Diversity Studies of Soybean [*Glycine max* (L.) Merrill] Germplasm Accessions using Cluster and Principal Component Analysis. *Legum Res - an Int J* 2023; 1: 1–6.

[84] Vianna VF, Unêda-Trevisoli SH, Desidério JA, Santiago SD, Charnai K, Júnior JAF, et al. The multivariate approach and influence of characters in selecting superior soybean genotypes. *African J Agric Res* 2013; 8(30): 4162–69. doi: 10.5897/AJAR2013.7064.

[85] Leite WS, Unêda-Trevisoli SH, Silva FM, Silva AJ, Di Mauro AO. Identification of superior genotypes and soybean traits by multivariate analysis and selection index. Rev Cienc Agron 2018; 49(3): 491–500.

[86] Ghiday T, Sentayehu A. Genetic Divergence Analysis on Some Soybean [*Glycine max* (L.) Merrill] Genotypes Grown in Pawe, Ethiopia. Am J Agric Environ Sci 2015; 15(10): 1927–33, DOI: 10.5829/idosi.aejaes.2015.15.10.12777.

[87] Khan MSA, Karim MA, Haque MM, Karim AJM, Mian MAK. Variations in agronomic traits of Soybean genotypes. *SAARC J Agric* 2014; 12(2): 90–100.

[88] Alina MM, Kingstone M. Genetic Differentiation of ARC Soybean [*Glycine Max* (L.) Merrill] Accessions Based on Agronomic and Nutritional Quality Traits. *Agric Food Sci Res* 2018; 5: 6–22.

[89] El-Hashash EF. Genetic diversity of soybean yield based on cluster and principal component analyses. *J Adv Biol & Biotechnol* 2016; 10(3): 1–9.

[90] Mahbub MM, Rahman MM, Hossain MS, Nahar L, Shirazy BJ. Morphophysiological variation in soybean [*Glycine max* (L.) Merrill]. *Am J Agric Environ Sci* 2016; 16(2): 234–

38, DOI: 10.5829/idosi.aejaes.2016.16.2.12687.

[91] Liu YL, Li YH, Zhou GA, Uzokwe N, Chang RZ, Chen SY, et al. Development of soybean EST-SSR markers and their use to assess genetic diversity in the Subgenus soja. *Agric Sci china* 2010; 9(10): 1423–1429. https://doi.org/10.1016/S1671-2927(09)60233-9.

[92] Gupta, S. K., & Manjaya JG. Genetic diversity and population structure of Indian soybean [*Glycine max* (L.) Merr.] revealed by simple sequence repeat markers. *J Crop Sci Biotech* 2017; 20(3): 221–231.

[93] Nawaz MA, Lin X, Chan TF, Lam HM, Baloch FS, Ali MA, et al. Genetic architecture of wild soybean (*Glycine soja Sieb*. and *Zucc*.) populations originating from different East Asian regions. *Genet Resour Crop Evol* 2021; 68: 1577-1588.

[94] Chiemeke FK, Olasanmi B, Agre PA, Mushoriwa H, Chigeza G, Abebe AT. Genetic Diversity and Population Structure Analysis of Soybean [*Glycine max* (L.) Merrill] Genotypes Using Agro-Morphological Traits and SNP Markers. *Genes (Basel)* 2024; 15: 1373.

[95] Yohane EN, Shimelis H, Laing M, Shayanowako A. Genetic diversity and grouping of pigeon pea [*Cajanus cajan* Millspaugh] Germplasm using SNP markers and agronomic traits. *PLoS One* 2022; 17(11). https://doi.org/10.1371/journal.pone.0275060

[96] Naflath TV, Rajendra PS, Ravikumar RS. Population structure and genetic diversity characterization of soybean for seed longevity. *PLoS One* 2022; 17: e0278631.https://doi.org/10.1371/journal.pone.0278631

[97] Chander S, Garcia-Oliveira AL, Gedil M, Shah T, Otusanya GO, Asiedu R et al. Genetic diversity and population structure of soybean lines adapted to Sub-Saharan Africa using single nucleotide polymorphism (SNP) markers. *Agronomy* 2021; 11(3): 604. **https://doi.org/10.3390/agronomy11030604**

[98] Kumar D, Chhokar V, Sheoran S, Singh R, Sharma P, Jaiswal S, et al. Characterization of genetic diversity and population structure in wheat using array-based SNP markers. *Mol Biol Rep* 2020; 47: 293–306.

[99] Brhane H, Hammenhag C. Genetic diversity and population structure analysis of a diverse

panel of peas (*Pisum sativum*). *Front Genet* 2024; 15: 1396888. https://doi.org/10.3389/fgene.2024.1396888.

[100] Nkhata W, Shimelis H, Melis R, Chirwa R, Mzengeza T, Mathew I, et al. Population structure and genetic diversity analyses of common bean germplasm collections of East and Southern Africa using morphological traits and high-density SNP markers. *PLoS One* 2020; 15(2): e0243238. https://doi.org/10.1371/journal.pone.0243238.

[101] Fatokun C, Girma G, Abberton M, Gedil M, Unachukwu N, Oyatomi O, et al. Genetic diversity and population structure of a mini-core subset from the world cowpea (*Vigna unguiculata* (L.) Walp.) germplasm collection. *Sci Rep* 2018; 8(1): 16035.

[102] Rani R, Raza G, Tung MH, Rizwan M, Ashfaq H, Shimelis H, et al. Genetic diversity and population structure analysis in cultivated soybean [*Glycine max* (L.) Merr.] using SSR and EST-SSR markers. PLoS One 2023; 18(5): e0286099.

[103] Yirgu M, Kebede M, Feyissa T, Lakew B, Woldeyohannes AB, Fikere M. Single nucleotide polymorphism (SNP) markers for genetic diversity and population structure study in Ethiopian barley (*Hordeum vulgare* L.) germplasm. *BMC Genomic Data* 2023; 24(1): 7.

[104] Sodedji FA, Agbahoungba S, Agoyi EE, Kafoutchoni MK, Choi J, Nguetta SP, et al. Diversity, population structure, and linkage disequilibrium among cowpea accessions. *The Plant Genome* 2021; 14(3): e20113.

[105] Carpentieri-Pípolo V, Pípolo AE, Silva FA, Petek MR. Soybean parent selection based on genetic diversity. *Brazilian Arch Biol Technol* 2000; 43: 295–300.

[106] Miller MJ, Song Q, Fallen B, Li Z. Genomic prediction of optimal cross combinations to accelerate genetic improvement of soybean (*Glycine max*). *Front Plant Sci* 2023; 14: 1171135.

[107] Lawson DJ, Falush D. Population identification using genetic data. *Annu Rev Genomics Hum Genet* 2012; 13(1): 337–61. **https://doi.org/10.1146/annurev-genom-082410-101510**

[108] Pham T. Analyses of genetic diversity and desirable traits in sesame *(Sesamum indicum*

L., Pedaliaceae) implication for breeding and conservation, Master's theses, Swedish University of Agricultural Sciences. 2011.

[109] Dey SS, Singh AK, Chandel D, Behera TK. Genetic diversity of bitter gourd (*Momordica charantia* L.) genotypes revealed by RAPD markers and agronomic traits. *Sci Hortic (Amsterdam)* 2006; 109: 21–28.

[110] Uba CU, Oselebe HO, Tesfaye AA, Abtew WG. Genetic diversity and population structure analysis of bambara groundnut (*Vigna subterrenea* L) landraces using DArT SNP markers. *PLoS One* 2021; 16(7): e0253600.

[111] Fu YB, Cober ER, Morrison MJ, Marsolais F, Peterson GW, Horbach C. Patterns of genetic variation in a soybean germplasm collection as characterized with genotyping-by-sequencing. *Plants* 2021; 10(8): 1611.

Supporting information

S1 Table. Eigenvalues, proportion of variance, and cumulative proportion of the studied soybean genotypes for eight agronomic and yield traits. D50F = days to 50% flowering, SHS = shattering score, RtNd = root nodule score, Lodg = lodging score, PH = plant height (cm), D95M = days to 95% maturity, HSW = hundred seed weight (gm), GY = grain yield (kg/ha).

S2 Table. List of genotypes and their grouping based on 10,630 SNP markers using the 70% cut-off criterion of the membership probability threshold.

S3 Table. Mean performances of the phenotypic traits of the study genotypes

S4 Table. Genetic distance matrix of the 45 soybean genotypes based on SNP markers.

S1 Fig. Summary statistics of 10,630 single nucleotide polymorphism (SNP) markers used for genotyping 45 soybean genotypes.

 Click here to access/download;Figure;Fig 1.tif.tif

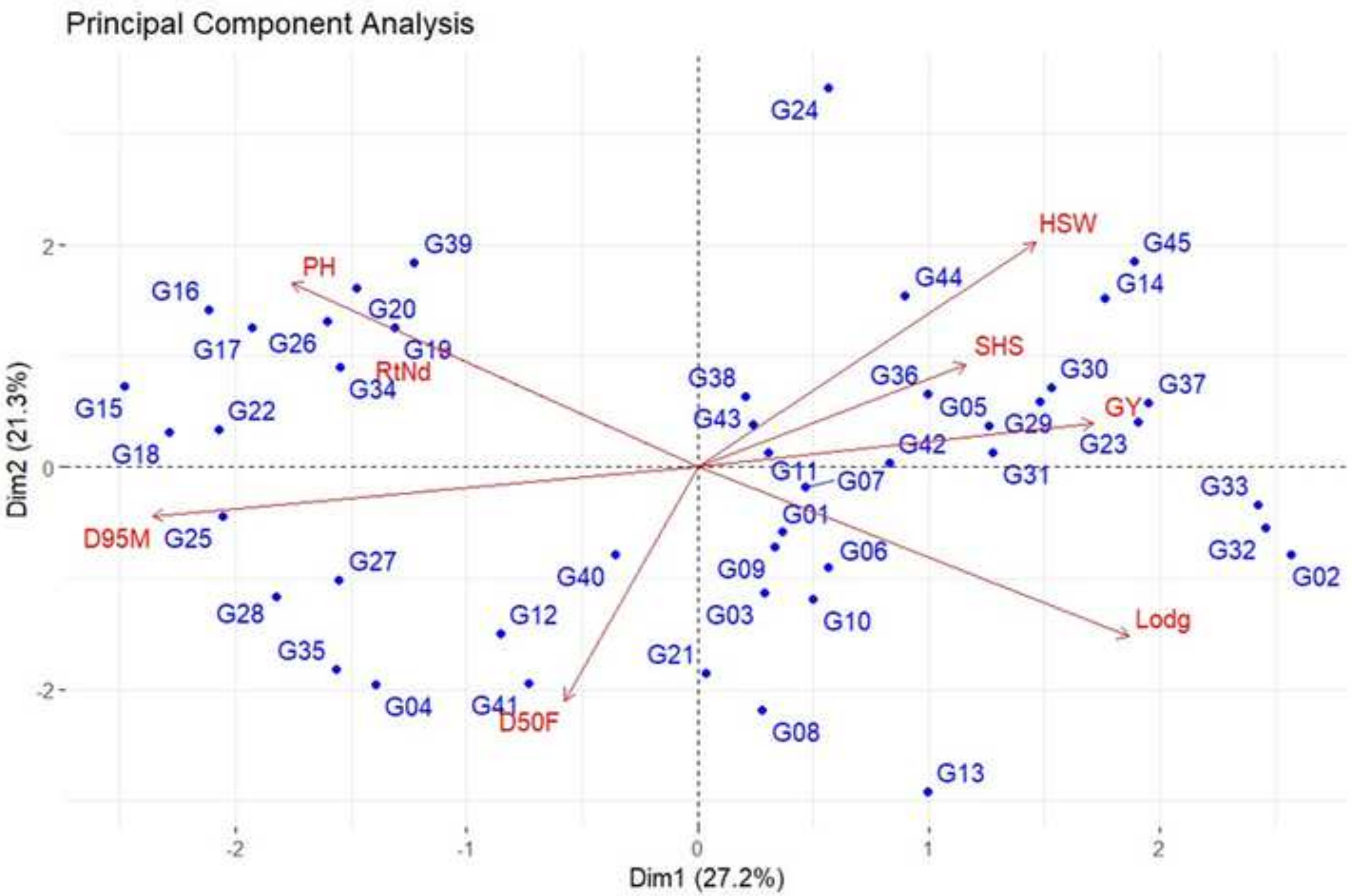

Figure

Click here to access/download;Figure;Fig 2.tif.tif

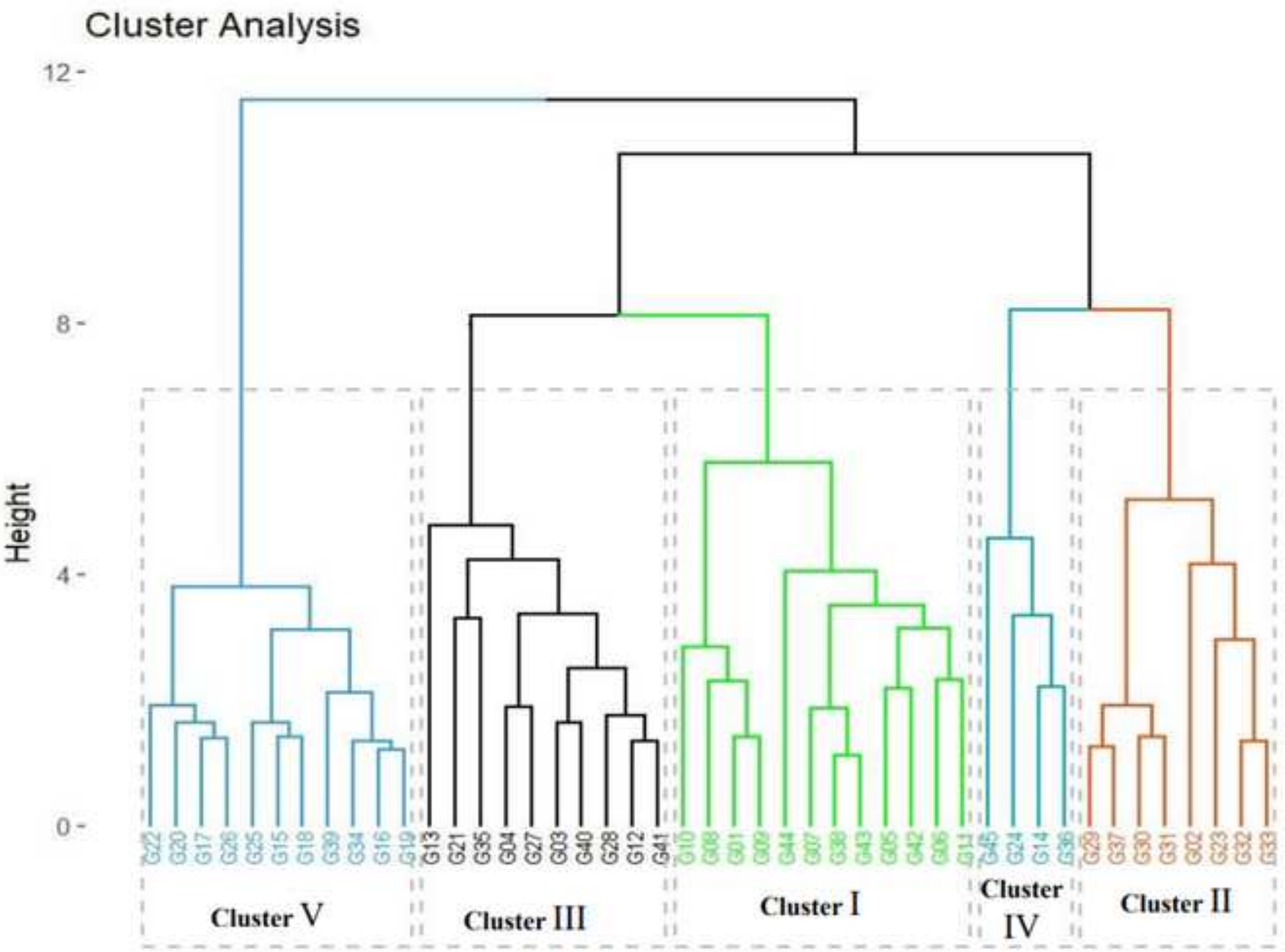

Figure Click here to access/download;Figure;Fig 3.tif.tif

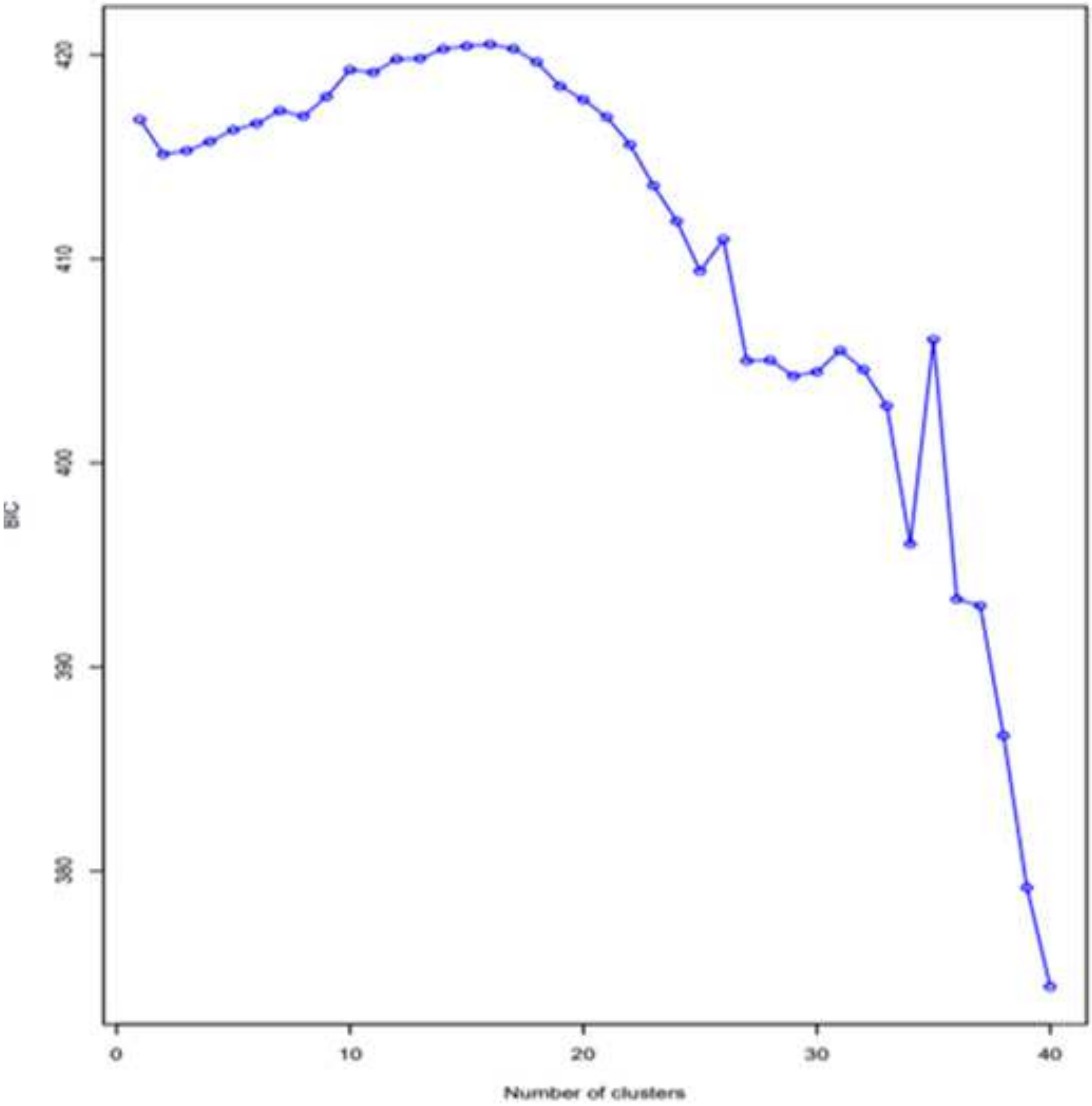

Figure

Click here to access/download;Figure;Fig 4. tif.tif

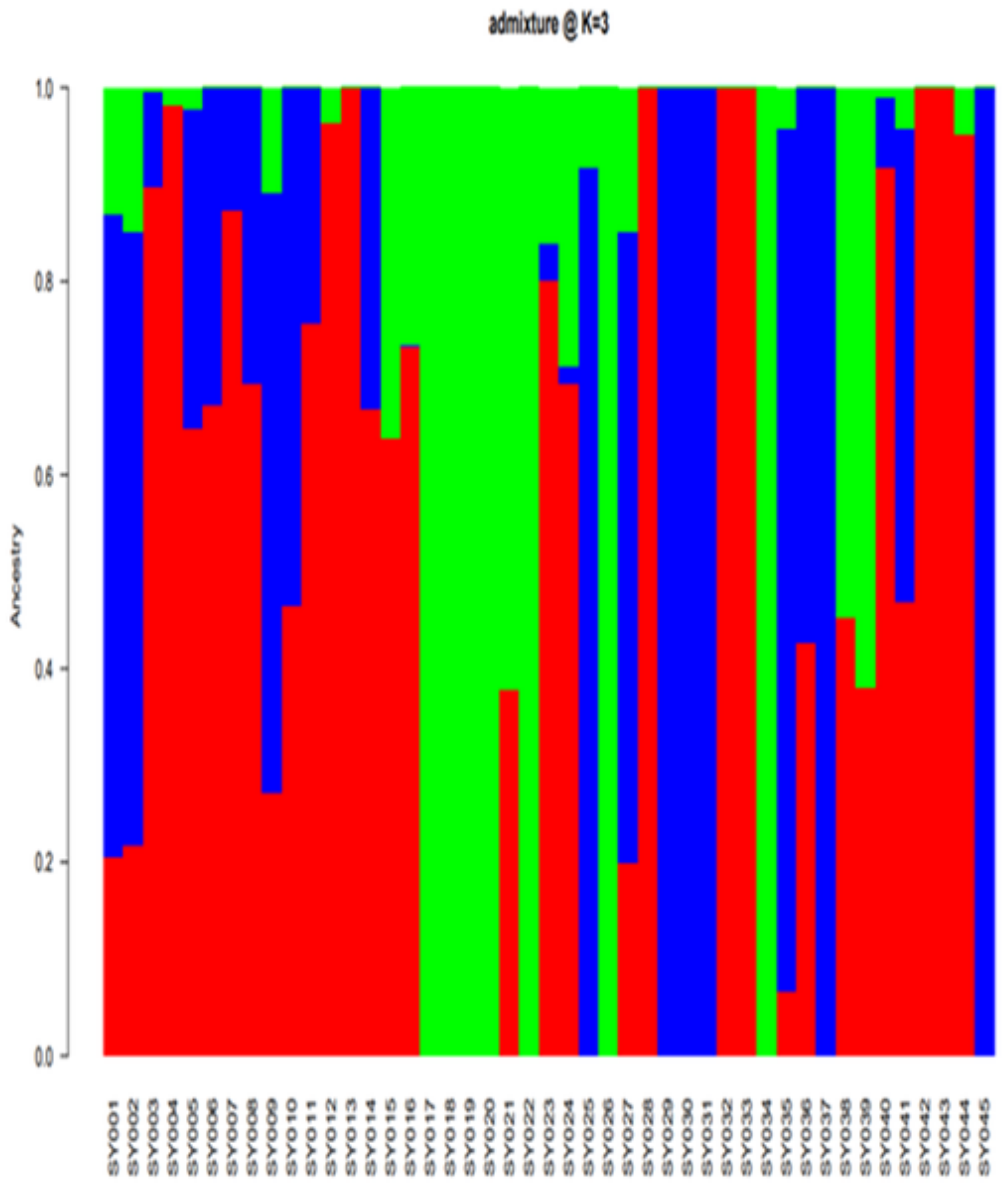

 Click here to access/download;Figure;Fig 5. tif.tif

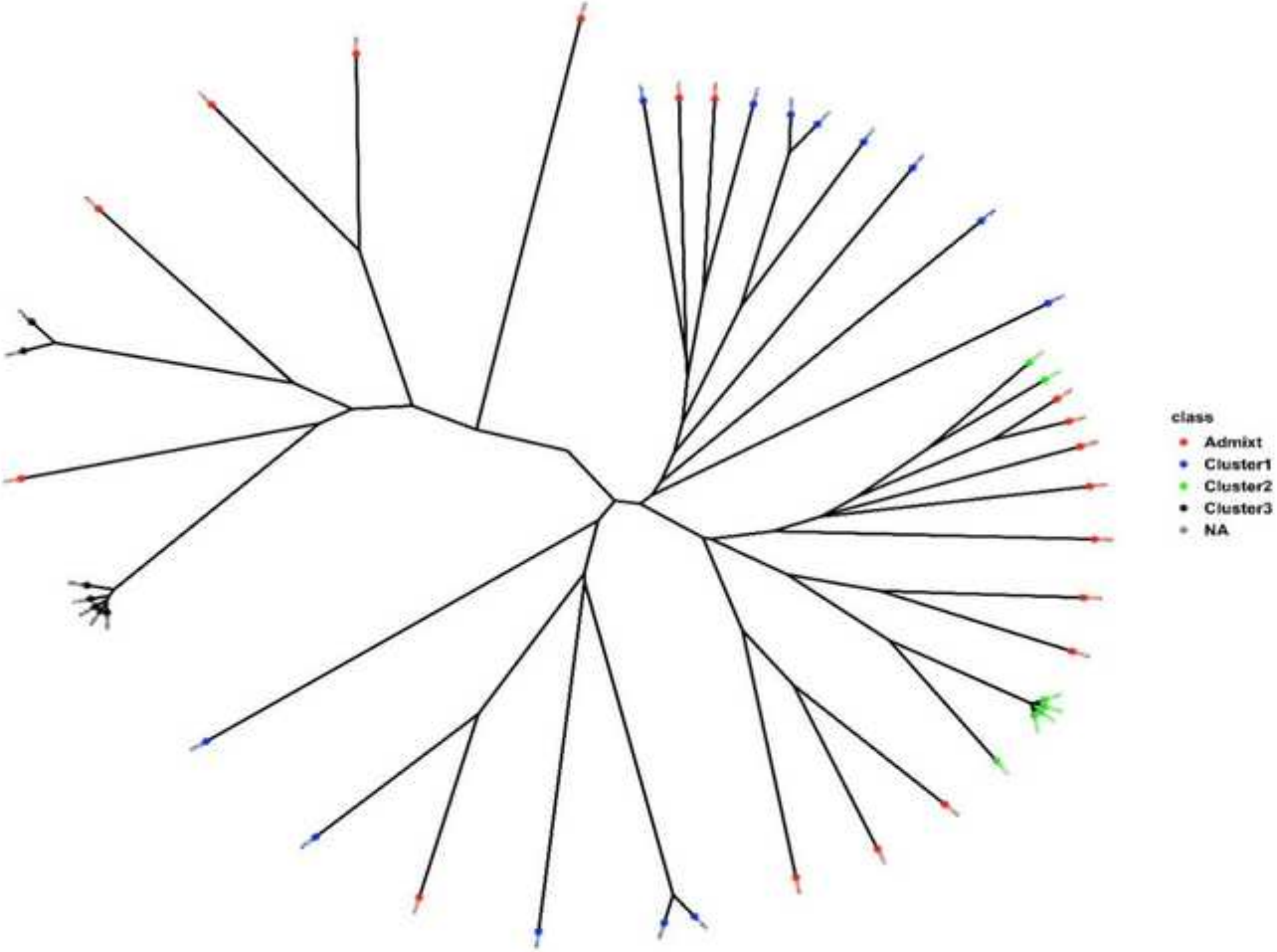

Figure

Click here to access/download;Figure;Fig 6. tif.tif

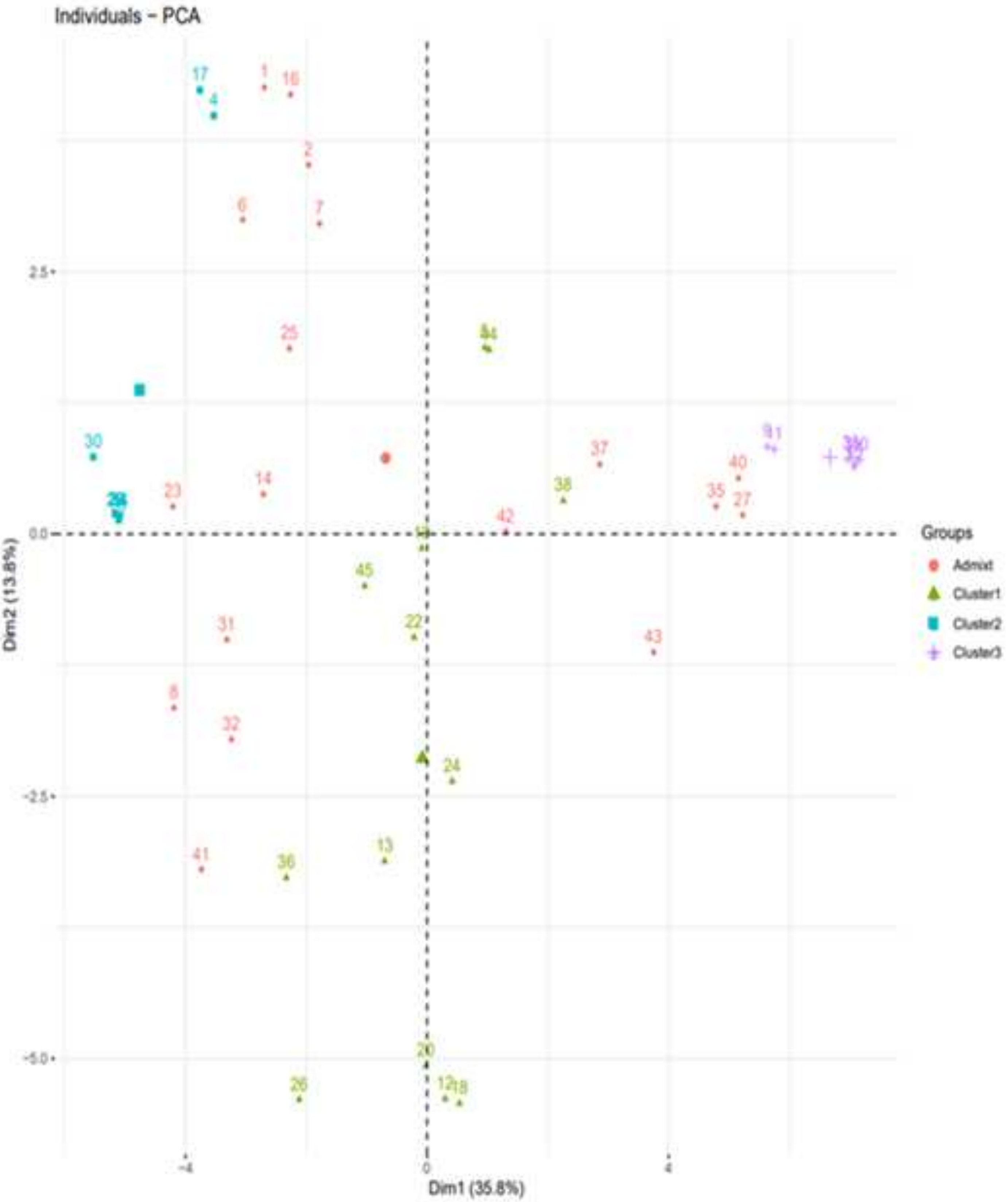

Click here to access/download
Supporting Information
S3. Table.docx

Click here to access/download
Supporting Information
S4. Table.csv

Click here to access/download
Supporting Information - Compressed/ZIP File Archive
Supplementary File.zip

Genetic diversity and population structure analysis of soybean [*Glycine max* (L.) Merrill] genotypes based on agro-morphological traits and SNP markers

Abebawork Tilahun Assfaw[1,2*], Bunmi Olasanmi[1,3], PaterneAgre[4], Godfree Chigeza[4], Hapson Mushoriwa[4], Fowobaje Kayode[4], Abush Tesfaye Abebe[4]

[1]Pan African University Life and Earth Science Institute (including Health and Agriculture), Ibadan, Nigeria

[2]Hawassa University College of Agriculture, Hawassa, Ethiopia

[3]Department of Crop and Horticultural Sciences, University of Ibadan, Ibadan, Nigeria

[4]International Institute of Tropical Agriculture, Ibadan, Nigeria

Corresponding author

E-mail: abebaworktilahun@gmail.com (ATA)

Abstract

Soybean (*Glycine max*) is one of the world's most important oilseed crops and has adapted to various environmental conditions. Yields of soybeans in Nigeria are notably low due to different production constraints, including the limited availability of improved varieties and the slow replacement rate of old varieties with new and high-yielding ones. Ensuring high genetic diversity in the working germplasm is among the primary factors for the success of breeding programs in identifying high-yielding and well-adapted improved varieties. This study aimed to assess the genetic diversity and population structure of 45 soybean breeding lines of the International Institute of Tropical Agriculture soybean breeding program at the advanced evaluation stage based on

phenotypic traits and SNP markers to support breeding strategies. Field trials were conducted in 2022 across three International Institute of Tropical Agriculture stations in Nigeria using a 5 × 9 alpha-lattice design with three replications. The collected yield and yield component data were subjected to analysis of variance, mean comparison, principal component analyses, and cluster analyses using R software. The genotypes were further assessed for SNPs-based genotypic diversity analyses. The combined analysis of variance revealed a significant genotype x location interaction for grain yield and a highly significant difference in days to 50% flowering and days to 95% maturity. The genotypes G02, G10, G11, G01, and G24 were significantly superior in grain yield. Principal component analysis showed that the first three components explained 64.8% of total variation, while hierarchical clustering grouped genotypes into five clusters based on phenotype and three clusters using SNP data. The SNP markers showed average observed heterozygosity, expected heterozygosity, minor allele frequency, and polymorphic information content of 0.08, 0.27, 0.20, and 0.22, respectively, which showed the existence of considerable genetic variation among the studied genotypes. The current study suggested that the genotypes under investigation exhibited a high genetic variation in the studied genotypes.

Keywords: Principal component analysis, Cluster analysis, SNP marker, Minor allele frequency, Admixture

Introduction

Soybean [*Glycine max* (L.) Merrill] is one of the leading oilseed crops globally, accounting for approximately 57% of global vegetable oil production ~~, is the leading oil seed crop, accounting for 57% of the global oil production~~ [1, 2]. It is cultivated in numerous countries and serves as a major source of vegetable oil and protein, essential in food, feed, and various industrial applications ~~It is~~

~~cultivated in many countries and is a major source of vegetable oil and protein used in food, animal feed, and for industrial purposes~~ [3]. With a protein level of 40–42% and an oil content of 18–22%, soybeans have twice as much protein as meat or chicken and all eight essential amino acids required for a child's healthy development [4]. Soybeans were domesticated around the 11th century BC in Northeast China and subsequently spread across Asia, the USA, Brazil, and Argentina ~~It was domesticated in the eleventh (11th) century BC around Northeast China and was then transported throughout Asia, the USA, Brazil, and Argentina~~ [5].

Nigeria ranks as the second-largest producer of soybeans in Africa, following South Africa~~Nigeria is the second-largest producer of soybeans in Africa, after South Africa~~ [6]. Soybeans were first introduced to Nigeria in 1908, with successful commercial cultivation beginning in 1937, using the Malayan variety in Benue State~~The first soybeans were brought into Nigeria in 1908, but the first successful cultivation was in 1937 with the Malayan variety, which was reported as commercial production in Benue State~~ [7]. The crop is adapted to diverse environmental conditions and is predominantly grown under rain-fed conditions~~The crop is adapted to various environmental conditions and is primarily cultivated under rain-fed conditions~~ [8]. Despite Nigeria being the second highest producer of soybeans in Africa, the national average yield is <1 ton/ha, which is far below the potential yield of the crop (over 3 tons/ha) [6]. However, the limited availability of high-yielding and disease-resistant varieties and the slow rate of replacement of old varieties with high-yielding and climate and stress-resilient varieties are among the major factors contributing to the low yields of soybeans. Hence, enhancing the genetic improvement of the crop to make high-yielding climate-resilient varieties available for production can have paramount importance in increasing soybean production, which will greatly benefit humanity, primarily by reducing malnutrition. Genetic improvement of soybeans plays a pivotal role in addressing malnutrition in

Nigeria through enhancing the crop’s nutritional composition [9]. Currently, efforts are underway by the IITA soybean breeding program to improve protein content, fatty acid profiles, and reduce anti-nutritional factors such as phytic acid and protease inhibitors, thereby increasing the crop's nutritional value and its role in combating malnutrition. Recent studies have shown that local soybean varieties differ significantly in their proximate composition, including protein and fat levels, highlighting the potential for selecting superior nutritional lines [10, 11]. Additionally, integrating biofortification strategies into soybean breeding can help enhance the supply of essential micronutrients like iron and zinc, which are commonly deficient in low-income populations [12, 13]. Thus, soybean improvement strengthens food security and serves as a tool for mitigating micronutrient deficiencies and protein-energy malnutrition [14, 15].

A fundamental step for success in any breeding program is evaluating and understanding the extent of genetic variability in the crop of interest [16]~~[9]~~. The genetic diversity of a crop species can be assessed using phenotypic traits and molecular markers [17]~~[10]~~. A phenotypic diversity study is the standard method of evaluating the extent of genetic diversity and determining the agronomic value and grouping of crop germplasm [18].~~[11]~~. Understanding the phenotypic variation and trait relationships helps crop breeders to develop more adaptable and productive varieties [17].~~[10]~~. Key traits, like number of seeds per plant/pod, number of pods per plant, 100 seeds weight, leaflet shape, flower color, stem architecture, number of days till flowering or maturity, plant height, pubescence type, and density, grain yield and other related factors are among the phenotypic qualities that are typically evaluated for genetic diversity of soybean [19-25]~~[12-18]~~. Several studies on phenotypic traits have found a high genetic diversity in soybean germplasms. Liu et al. [26]~~[19]~~ reported high phenotypic variation in characterizing 138 soybean accessions based on yield and yield-related agro-morphological traits. Similarly, Bairagi et al. [27] ~~[20]~~ reported high

genetic variation among 32 soybean genotypes based on ten morphological traits, while Kuswantoro et al. [28] [21] studied the phenotypic diversity of 100 soybean genotypes and reported significant variations for all the agronomic traits. Based on nine agro-morphological traits, Marconato et al. [29][22] also reported the existence of high genetic diversity among the 93 soybean accessions maintained by the Brazilian Agricultural Research Corporation (EMBRAPA) gene bank. All the aforementioned findings indicate that the agro-morphological traits were helpful in assessing genetic diversity that facilitates the utilization of the genetic resources for the genetic improvement of the crop. In crop species and their relatives, selection based on phenotypic features is still frequently used and will likely continue to be an important approach in determining the extent of diversity [25].[18].

Molecular markers are the preferred approach for assessing genetic diversity due to their excellent repeatability, superior genome coverage, automation potential, great variability, neutrality, and lack of sensitivity to environmental variations [30][23]. There are reports on using different types of molecular markers for diversity and population structure studies in soybeans. However, SNP markers are the most commonly used molecular markers in genetic diversity in most of the recent studies, given that they are extensively spread across the plant's genome [31][24]. This is due to its affordability, target accuracy, and codominant character [32, 33].[25, 26]. Genetic diversity studies based on SNP markers were conducted on various crops including soybeans [34-37][27-30] cassava [33][26]; maize [38-40][31-33]; and yam [41-43][34-36]. Despite, the great significance of assessing the phenotypic and genotypic diversity, the study materials that were developed by the IITA soybean breeding program and were at an advanced stage of evaluation have not been assessed for their genetic diversity based on both the phonotypic traits and molecular markers. Therefore, the objectives of this study were to evaluate the genetic diversity and

population structure of the advanced soybean breeding lines of the IITA soybean breeding program for yield and yield-related traits using agro-morphological traits and SNP markers to recommend the best-performing varieties for direct production or use as parental lines for future genetic improvement of the crop.

Materials and Methods

Description of the study area

The field experiments were conducted across three stations i.e., Ibadan, Zaria, and Ikenne of the International Institute of Tropical Agriculture (IITA), Nigeria in the 2022 cropping season. The study locations represent Nigeria's different soybean production environments and are characterized by different agroclimatic conditions presented in Table 1.

Table 1. Trial locations and their respective agro-climatic descriptions.

Location	Ecology	State	Long.	Lat.	Elevation (masl)	Rainfall (mm)	Temp.(°C)	
							Min	Max
IITA, Ibadan	Derived Savannah	Oyo	7°30′N	3°54′E	243	1300-1500	22	32
Ikenne	Lowland humid forest	Ogun	6°52′N	3°43′E	235.2	1200	24	30
Zaria	Northern Guinea. Savannah	Kaduna	11°11′N	7°38′ E	600	1045	17.1	35.4

masl: meter above sea level, mm: millimeter, °C: degree Celsius Source: [44, 45]~~[37, 38]~~.

Experimental design and management

The field trial was laid out in a 5 x 9 alpha lattice design with three replications. Each entry was planted in a plot of 4 rows of 4 m length. The spacings between rows and plants were 50 cm and

5 cm, respectively. The two middle rows were harvested to measure plot yield and other related traits, and two border rows were left to exclude the border effect. A mixture of NPK and TSP fertilizers was applied at 25g/row at a 1:2 ratio at planting. The seeds were inoculated with *Bradrhizobium japonicum* inoculant called Nodumax, manufactured by the IITA Business Incubation Platform (BIP). All the rest of the management practices were applied as per the recommendation for the crop [46][39].

Experimental materials

A total of 45 soybean breeding lines, which are part of the working germplasm of the IITA soybean breeding program, along with the IITA check (TGX-1951-3F a variety developed by IITA and released in Nigeria) and a commercial check (SC-Signa, a variety released by a private company called SeedCo in Nigeria) were used in the study. Among the entries, 38 genotypes were developed by and sourced from the soybean breeding program of IITA that were at an advanced stage of yield trials in Nigeria; four from the USDA soybean genetic resource center, one from Ghana, and one from Uganda. The study genotypes with their corresponding sources are presented in Table 2.

Table 2. The pedigrees, code, and source of 45 soybean genotypes were used in the study.

No	Pedigree	Genotype Code	SNP Designation	Source
1	TGx1951-4F	G01	SY001	IITA
2	TGx1993-18FN	G02	SY002	IITA
3	TGx2015-2E	G03	SY003	IITA
4	TGx1989-11FxTGx1987-10F-1-1-3-1-3-I	G04	SY004	IITA

5	TGx1961-1FxH10-2-3-7-2-1	G05	SY005	IITA
6	TGx1961-1FxTGx1835-10E-1-1-4-2-1-E	G06	SY006	IITA
7	TGx1987-9FxTGx1835-10E-1-2-2-1-1	G07	SY007	IITA
8	TGx1987-11FxH7-3-1-1-1-2-3-I	G08	SY008	IITA
9	TGx1987-11FxH7-3-1-1-1-2-6-I	G09	SY009	IITA
10	TGx1989-19FxTGx1987-10F-5-3-1-2-2-I	G10	SY010	IITA
11	TGx1989-45FxTGx1835-10E-3-2-1-3-3-E	G11	SY011	IITA
12	TGx2022-3E	G12	SY012	IITA
13	TGx1987-62FxH7-1-1-4-3-2-E	G13	SY013	IITA
14	TGx1990-38FxTGx1835-10E-1-4-3-1-1-E	G14	SY014	IITA
15	TGx2029-21F	G15	SY015	IITA
16	Panaroma-3	G16	SY016	USDA
17	TGx2029-7F	G17	SY017	IITA
18	Panaroma-1	G18	SY018	USDA
19	TGx2029-31F	G19	SY019	IITA
20	TGx2029-49F	G20	SY020	IITA
21	Panorama-27D	G21	SY021	USDA
22	TGx2029-53F	G22	SY022	IITA
23	TGx2014-16FM	G23	SY023	IITA
24	Sc-Signa	G24	SY024	Seedco
25	Songda	G25	SY025	SARI, Ghana
26	TGx2029-39F	G26	SY026	USDA
27	TGx1951-3F	G27	SY027	IITA-Check
28	(TGx1987-9F/TGx1740-2F)-#F5-1025-1	G28	SY028	IITA
29	(TGx1740-2F/MW1)-#F6-2002-10-15	G29	SY029	IITA
30	(TGx1740-2F/MW1)-#F6-2002-10-19	G30	SY030	IITA
31	(TGx1740-2F/MW1)-#F6-2002-10-28	G31	SY031	IITA
32	(TGx1830-20E/UG5)-#F6-2014-27-5	G32	SY032	IITA
33	(TGx1830-20E/UG5)-#F6-2014-27-6	G33	SY033	IITA
34	TGx2029-22F	G34	SY034	IITA
35	TGx1448-2E	G35	SY035	IITA check
36	TGx1989-19F	G36	SY036	IITA check
37	(TGx1740-2F/MW1)-#F6-2002-10-11	G37	SY037	IITA
38	TGx2029-20F	G38	SY038	IITA
39	TGx2029-27F	G39	SY039	IITA
40	(TGx1987-10F/TGx1740-2F)-#F5-1011-6	G40	SY040	IITA
41	(TGx1987-10F/TGx1740-2F)-#F5-1011-8	G41	SY041	IITA
42	(TGx1987-62F/MW1)-#F5-1006-15	G42	SY042	IITA
43	(TGx1987-62F/MW1)-#F5-1006-22	G43	SY043	IITA
44	Maksoy-4N	G44	SY044	Uganda
45	(TGx1740-2F/MW1)-#F6-2002-10-21	G45	SY045	IITA

Phenotypic data collection

The agro-morphological traits of plant height and root nodule score were determined from the average values of five randomly selected plants of each genotype, whereas days to 50% flowering, days to 95% maturity, lodging score, shattering score, hundred seed weight and grain yield were collected on a plot basis~~days to 50% flowering, days to 95% maturity, lodging, root nodule, shattering, hundred seed weight, and grain yield were recorded~~ from the trials following the soybean descriptor of IBPGR [47] ~~[40]~~ as shown in Table 3.

Table 3. List of traits and their measurements.

SN	Traits	Code	Measurement
1	Plant height (cm)	PH	measured in cm using a centimeter ruler as the height of the plant from the base to the tip of the plant at maturity
2	Days to 50% flowering (days)	D50F	The number of days taken from sowing to 50% of the plant bears at least one flower
3	Days to 95% maturity (days)	D95M	Recorded when at least 95% of the pods attained a mature brown color.
4	Lodging (score)	Lodg	All plants in the middle two rows were scored using a scale of 1 to 5; where 1=all plants erect, 2=slight lodging, 3=plants lodged at a 45-degree angle, 4= severe lodging, and 5=all plants flat.
5	Root Nodule (score)	RtNd	Five randomly selected plants from the border rows were uprooted and visually scored using a scale of 1 to 5; where 1=no nodules, 2= with few nodules, 3 = half of the roots have nodules, 4 = more than half of the roots have nodules, and 5=all roots have nodules
6	Shattering (score)	SHS	was scored from the border rows, one week after harvesting the middle rows, using a scale of 1 to 5; where 1=very tolerant 2=tolerant, 3=moderately tolerant, 4=highly susceptible, and 5=very highly susceptible
7	Hundred seed weight (g)	HSW	The weight of a hundred seeds selected at random from each of the genotypes was recorded and expressed in grams

8	Grain yield (kg)	GY	Seeds from plants harvested from the middle two rows and contributing to the yield sample in the net plot were subjected to uniform drying conditions, weighed in grams, and converted to kilograms per hectare.

Phenotypic data analysis

Analysis of variance and Mean comparisons

The quantitative data were subjected to a combined analysis of variance to test for significant differences among genotypes using the linear mixed model (LMER) procedure of the R software package (version 4.3.1, 2023). The mean comparisons were done using the least significant difference (LSD) at a 5% level of significance.

Formatted: Font: 12 pt, Not Bold

Cluster analysis

Cluster analyses were used to group the genotypes into homogeneous forms based on quantitative characters. Hierarchical clustering was conducted using the Euclidean distance and Ward's D^2 method, based on similarity coefficients among the 45 soybean genotypes. The analysis was performed in R software (version 4.3.1, R Core Team, 2023) utilizing the 'dendextend' package.

Principal components analysis (PCA)

Principal component analysis (PCA) was computed to determine the traits that accounted for much of the total variation and to assess the extent of genetic diversity in the studied genotypes. The analysis was performed using ‘FactoMiner’ and ‘factoextra’ libraries of R software [48]~~[41]~~. In this analysis, only principal components (PCs) with eigenvalues greater than one were considered important for the total variations.

Genotyping

Leaf sampling, DNA extraction, and genotyping data processing

Seeds of the 45 genotypes were sown in the screen house at IITA, Ibadan, for sampling. For the analysis, immature, healthy trifoliate leaves from three-week-old seedlings were collected from four to five plants of each of the 45 genotypes (Table 2) using a leaf puncher and kept in a zip-lock bag on ice and later stored at −80 ◦C in a deep freezer dryer. Before genomic deoxyribonucleic acid (DNA) extraction, each sample leaf was bulked and lyophilized for 72 hours in a Labconco Freezone 2.5 L System lyophilizer (Marshall Scientific, LABCONCO, Kansas, MO, USA) and reduced to a fine powder in the SpexTM Sample Prep 2010 Geno/Grinder (Thomas Scientific, Metuchen, NJ, USA). The deoxyribonucleic acid (DNA) was extracted using a technique developed by Intertek-AgriTech (http://www.intertech.com/agriculture/agritech/), accessed 16 January 2024, and based on the LGC oKtopure™ automated high-throughput 'sbeadex™' DNA extraction and purification system (https://www.biosearchtech.com/), accessed 16 January 2024.

Magnetic separation was used in the 'sbeadex™' technique to prepare nucleic acids. The first stage in this process was to homogenize leaf tissue samples in 96 deep-well plates using steel bead grinding. The ground tissue was treated with a DNA extraction buffer using LGC's 'sbeadex™' kit for plant DNA preparation (https://www.biosearchtech.com/, accessed 16 January 2024). Finally, super-paramagnetic particles coated with 'sbeadexTM' surface chemistry that catches nucleic acids from a sample were used to purify extracted DNA. Purified DNA was eluted and used in downstream procedures. Medium-throughput genotyping was conducted in a 96 plex DArTseq protocol, and SNPs were called using the DArT's proprietary software, DArTSoft, as described by

Kilian et al. [49][42]. Each sequencing result's reads and tags were mapped to the G. max reference genome, which was used to convert the raw HapMap file to a Variant Call Format (VCF).

Genotypic data analysis

Using the software PLINK 1.9 and VCF tools, DArTseq SNP-derived markers were filtered to remove low-quality SNP markers. A total of 59,126 SNP markers were identified from the raw data before quality assessment. The SNP markers with minor allele frequencies <0.01, markers with >20% missing data, unmapped markers to any chromosome, and duplicated markers were removed. In the end, 10,630 informative SNP markers were retained and used for the subsequent analysis. Diversity indices statistics, such as observed heterozygosity (Ho), expected heterozygosity (He), minor allele frequency (MAF),) and the polymorphic information content (PIC) was estimated using VCFtools [50][43] and PLINK 1.9 [51][44]. Ho was calculated with the method suggested by Chesnokov and Artemyeva [52]

$$\text{Ho} = \frac{\text{Total number of heterozygote individuals}}{\text{Sample size}} \qquad \text{Equation 1}$$

He was determined following the equations given by Liu [53]

$$\text{He} = 1 - \sum \text{pi}^2 \qquad \text{Equation 2}$$

Where the summing is over all possible alleles, and **pi** is the frequency of the ith allele

The MAF values were calculated using Xue et al. [54] equation as follows

$$\text{MAF} = \frac{x_i}{x} \qquad \text{Equation 3}$$

where Xi is the number of minor alleles detected at a point, and X is the total number of genotypes detected at a point

In the same way, the PIC values were calculated by the following formula: Amiryousefi et al. [55]

$$PIC = 1 - \sum pi^2 - \sum \sum pi^2 pj^2 \qquad \text{Equation 4}$$

Where the first summation reflects the entire number of alleles (i^{th} and j^{th} alleles have population frequencies of pi and pj, respectively), whereas the two subsequent summations represent all i and j, where $i \neq j$.

Bayesian information criterion (BIC) was used to estimate the optimum sub-populations (K) using discriminant analysis of principal components (DAPC) and which was implemented in R using the ‘adegenet’ package [56][45]. Using the ancestry probability, the level of admixture was estimated, and individuals were assigned to a specific population when their membership coefficient in that group was ≥0.70. Genotypes with membership coefficients less than 0.70 at each assigned K were considered admixed. Coefficients of similarity showing genetic distances among the soybean lines were calculated in R software following Gower’s Distance model [57].

Results

Combined analysis of variance for Agronomic Traits over locations

The results from the combined analysis of variance (ANOVA) across three locations are presented in Table 4. The combined ANOVA revealed a significant ($p \leq 0.05$) genotype x location interaction for grain yield and a highly significant ($P<0.001$) difference for days to 95% maturity and days to 50% flowering. However, a non-significant genotype x location interaction was observed for shattering, root nodules, lodging, plant height, and hundred seed weight. The genotype effect was highly significant ($p \leq 0.001$) for all the traits except for hundred seed weight. The location effect

also showed a significant effect (P <0.05) for most traits, except for lodging, days to 50% flowering, and hundred seed weight.

Table 4. Mean squares for eight agronomic and yield traits of 45 soybean genotypes evaluated in three agroecologies of Nigeria

SOV	Genotype (G)	Location(L)	GxL
DF	44	2	88
SHS	1.13***	4.25*	0.39ns
RtNd	1.14***	71.3***	0.68ns
Lodg	1.2***	0.22ns	0.41ns
PH	839***	1010***	138ns
D50F	42.7***	27.7ns	34.9***
D95M	37.3***	376***	88***
HSW	93ns	126ns	76.2ns
GY	846000***	7900000***	501000*

*, *** Significant difference at $p<0.05$ and $p<0.001$, ns = non-significant, SOV = source of variation, DF = degree of freedom, GxL = genotype by location interaction, SHS = shattering score, RtNd = root nodule score, Lodg = lodging score, PH = plant height in centimetre, D50F=days to 50% flowering, D95M = days to 95% maturity, HSW = hundred seed weight in gram, GY = grain yield in kilogram per hectare.

Mean performance of the soybean genotypes for yield and yield-related traits

The mean comparisons of agronomic and yield-related traits that were determined during the study are presented in **S3** Table. High mean yields of 3310, 3210, 3060, 3050, and 2990 kg/ha were recorded for genotypes G02, G10, G11, G01, and G24, respectively. The lowest-yielding genotypes were G35 (1700 kg/ha), G15 (2020 kg/ha), G18 (2040 kg/ha) and G28 (2140 kg/ha). Days to 95% maturity varied from 112 to 121 days with G32 and G33 maturing earlier (112 days)

than the rest, while G25 was relatively late maturing (121 days). Mean days to maturity value of 117 days were recorded for genotypes G02, G26, G36, G39, G40 and G45. Days to 50% flowering varied from 44 to 55 days with the earliest genotype being G23 (44 days) followed by G33 with 45 days and G24 and G39 with 46 days. The genotypes that took longer to flower were G04, G05, G08, and G13 all with 55 days. Shattering ranged from 0.96 to 3.5 with an average of 1.3. Genotype G45 had a higher shattering score of 3.5. Lower shattering scores were recorded by G43 (0.96), G27, G40, and G07 (0.97). The maximum lodging score was recorded by G44 (2.43), G01 (2.4), and G08 (2.3), while the minimum was observed in G22 (1), G20 (1.02), and G17 (1.06). The root nodule score ranged from 2.17 to 3.61 with an average of 3.13. Among the 45 soybean genotypes, 27 genotypes nodulated more efficiently (above average) and the best were genotypes G02, G16 (3.61), G24, G29, G45 (3.56), and G01, G07, G15 (3.50). Genotypes G17 (102 cm), G20 (98.6 cm), G16 (96.4 cm), G19 (95.2 cm) and G26 (94.3cm) were found to be the tallest. Genotypes G02 (63.6 cm), G03 (64.8 cm), G31 (67.4 cm), and G12 (68.3 cm) were the shortest genotypes in this study.

Principal component analysis (PCA)

The first three principal components with eigenvalues greater than one contributed to 64.8% of the total variation among the genotypes (Table 5~~4~~, **S1** Table). The first principal component (PC1) accounted for 27.2% of the total variation. The lodging score showed a high and positive association, while days to 95% maturity had a negative association with PC1~~Lodging, grain yield, hundred seed weight, and shattering showed a high and positive association with PC1~~. The considerable variation observed in the second principal component (21.3%) was positively and highly correlated with hundred seed weight and~~,~~ plant height, ~~and nodulation,~~ and negatively correlated with days to 50% flowering ~~and lodging~~. The third principal component accounted for

16.3% of the total variation and had a high positive correlation with nodulation and days to 50% flowering~~, shattering, and grain yield~~. The biplot based on PC1 and PC2 that captured 27.2 and 21.3% of the total variation, respectively, with a cumulative contribution of 48.7% (Fig 1) displayed that genotypes G15, G24, G45, G13, and G02 were far from the origin, whereas G23, G43, G11, G07, G01, and G09 were located close to the origin.

Fig 1. Distribution of genotypes and traits in PCA-Biplot of the soybean genotypes evaluated for eight agronomic and yield-related traits. Where SHS = shattering score, RtNd = root nodule score, Lodg = lodging score, PH = plant height (cm), D50F = days to 50% flowering, D95M = days to 95% maturity, HSW = hundred seed weight (gm), GY = grain yield (kg/ha).

Table 54. Eigenvalues, percent of the variance, and cumulative variance of the first three PCs of the studied soybean genotypes for eight agronomic and yield traits.

Traits	PCs		
	I	II	III
	Eigenvectors		
D50F	-0.13	-0.53	0.51
RtNd	-0.25	0.27	0.65
Lodg	0.42	-0.38	0.21
SHS	0.26	0.23	0.38
PH	-0.39	0.42	0.04
D95M	-0.53	-0.11	0.17
HSW	0.33	0.51	0.12
GY	0.38	0.10	0.29
Eigenvalues	2.17	1.71	1.30
~~Total~~ Partial variation (%)	27.2	21.3	16.3

Cumul. variation (%)	27.2	48.5	64.8

PCs = Principal components, SHS = shattering score, RtNd = root nodule score, Lodg = lodging score, PH = plant height (cm), D50F = days to 50% flowering, D95M = days to 95% maturity, HSW = hundred seed weight (gm), GY = grain yield (kg/ha).

Cluster analysis

The cluster analysis resulting from the hierarchical ascending classification (HAC) grouped the soybean genotypes into five cluster groups, as shown in Table 6~~5~~ and Fig 2.

Fig 2. Dendrogram of the studied soybean genotypes evaluated across three locations in Nigeria.

Cluster I had the highest number of genotypes (12); while cluster IV had the lowest number of four genotypes. Genotypes in cluster I were high-yielding, relatively the highest lodged, had the highest number of nodules, and the latest flowering. The genotypes in cluster II were also high yielding, earliest flowered and matured, short height, and lowest nodulation. Cluster III consisted of late flowering and maturing, low yielding, low hundred seed weight, and shattering tolerant genotypes. In contrast, genotypes in cluster IV had the highest yield, heaviest hundred seed weight, and the highest number of nodulations, and were moderately tolerant to shattering. Cluster V is composed of tall and late-maturing genotypes with the lowest values for lodging.

Table 6~~5~~. Cluster mean values of the studied agro-morphological traits of the soybean genotypes and their clustering.

	Clusters				
Traits	I (12)	II (8)	III (10)	IV (4)	V (11)
D50F	52	47	53	48	47
D95M	112	109	115	112	116

GY (kg/ha)	2695.49	2688.74	2319.34	2786.85	2357.05
HSW (g)	15.07	15.31	12.99	16.18	14.00
Lodg	2.05	1.89	1.76	1.49	1.23
PH (cm)	81.35	70.92	73.09	76.73	92.10
RtNd	3.33	2.67	3.06	3.33	3.24
SHS	1.25	1.22	1.16	2.36	1.17

NB: Numbers in parenthesis are the number of genotypes in that cluster, D50F = days to 50% flowering, D95M = days to 95% maturity, GY = grain yield in kilogram per hectare, HSW = hundred seed weight in gram, Lodg = lodging, PH = plant height in centimeter, SHS = shattering, RtNd = nodulation.

Genetic diversity of soybean displayed by SNP markers

The results of the SNP markers' statistics are summarized in Table 7 ~~6~~ and **S1** Fig. The observed heterozygosity ranged from 0.00 to 0.16, with a mean of 0.08, while the expected heterozygosity (He) ranged between 0.00 and 0.50, with a mean of 0.27. The minor allele frequency (MAF) ranged between 0.01 and 0.50 with a mean value of 0.20 and the polymorphic information content (PIC) ranged from 0.00 to 0.38 with an average value of 0.22.

Table 7 ~~6~~. Diversity indices statistics of the 45 soybean genotypes based on 10,630 SNP markers.

	Ho	He	MAF	PIC
Minimum	0.00	0.00	0.01	0.00
Maximum	0.16	0.50	0.50	0.38
Mean	0.08	0.27	0.20	0.22

Ho = observed heterozygosity, He = expected heterozygosity, MAF=minor allele frequency, PIC=polymorphic information content

The population structure analysis in this study identified three sub-populations among the 45 soybean genotypes based on the optimal K=3 determined based on the BIC method (Fig 3)**.**

Fig 3. Graph showing the number of clusters vs the Bayesian Information Criterion (BIC).

Using the 70% cut-off criterion of the membership probability threshold, 27 genotypes were successfully assigned to the three different sub-populations. The remaining 18 genotypes with a probability of associations of less than 70% were considered as an admixed population (Fig 4, **S2** Table).

Fig 4. Population structure of the studied soybean genotypes based on the SNP markers. Red, blue, and green color denotes sub-populations I, II, and III, respectively.

Sub-population I (red) was comprised of 13 genotypes that were sourced from IITA and Uganda, while the seven genotypes were allocated in sub-population II (blue) that were sourced from Ghana and IITA. Sub-population III (green) consisted of seven genotypes obtained from USDA and IITA. The phylogenetic tree also showed three sub-populations with higher degrees of admixture similar to the BIC (Fig 5).

Fig 5. The phylogenetic trees of the studied soybean genotypes based on the SNP markers. Dots indicating individual genotypes.

Similarly, the DAPC assigned the genotypes to three cluster groups and clearly showed a higher degree of admixture among the genotypes. The first and second PCs accounted for 35.8 and 13.8% of the total variation, respectively. The members of Cluster III were the most compact in distribution, while those of Cluster I were the most widely distributed along with the axes of the first two PCs (Fig 6).

Fig 6. Scatter plot based on the first two PCs obtained from DAPC based on the SNP markers displaying the distribution of the studied genotypes. Note Each color corresponds to population structuring and grouping.

Genetic distance between genotypes based on 10,630 SNP markers

The genetic distances across the genotypes are summarized in **S4** Table. In cluster one, the highest genetic distance (0.335) was observed between genotypes SY007 and SY024, while the lowest genetic distance (0.14) was observed between genotypes SY013 and SY012. In cluster two, the maximum genetic distance (0.335) was observed between genotypes SY026 and SY030, while the minimum genetic distance (0.012) was observed between genotypes SY037 and SY029. In cluster three, the widest genetic distance (0.335) was observed between genotypes SY030 and SY026, while the shortest genetic distance (0.014) was observed between genotypes SY026 and SY020.

Discussion

Genetic diversity analysis in crops is required for the success of any plant breeding effort [58]~~[46]~~. Hence, evaluating crops' population structure and genetic diversity is vital for adopting effective genetic resource management and conservation measures [59]~~[47]~~. The genotype x location interactions across the three locations were significant for grain yield, and highly significant for days to 95% maturity, and days to 50% flowering, implying genotypes exhibited varying relative performances across locations for these traits. The results are in line with the findings of Tesfaye et al. [60], Krisnawati and Adie [61], and Pedro [62], who reported a highly significant genotype x environment interaction for days to 50% flowering, days to 95% maturity, and grain yield. Njoroge et al. [63], Nachilima [64], and Abebe et al. [65] also reported significant genotype x environment interaction for grain yield. Five genotypes i.e., G02, G10, G11, G01, and G24 showed significantly superior performance for grain yield (**S3** Table). These genotypes can be released as varieties for direct production by farmers after further evaluation or can be used as parents in the

soybean hybridization programs. Getnet [66], Beyene et al. [67], and Thio et al. [68] also obtained different mean yield performances among soybean genotypes in their studies.

Days to 95% maturity varied from 112 to 121 days, with G32 and G33 maturing earlier than the rest, while G25 maturing relatively late. Beyene et al. [67], Thio et al. [68], Sileshi [69], Goonde and Ayana [70] and Yirga et al. [71] reported comparable trends in variability in days to maturity for different soybean genotypes. Days to 50% flowering varied from 44 to 55 days with the earliest genotype being G23, followed by G33. The genotypes that took longer to flower were G04, G05, G08, and G13 all with 55 days. The results for days to 50% flowering agree with the findings of Goonde and Ayana [70] and Jandong et al. [72], and Akter [73]. Shattering ranged from 0.96 to 3.5 with the highest tolerant genotypes being G43, G07, and G27, whereas G45 was highly susceptible to pod-shattering. This result aligns with Fatima et al. [45] and Aondover et al. [74], who reported different mean performances in pod shattering. G44 recorded the maximum lodging score, while the minimum was observed in G22. This aligns with the work of Antwi-Boasiako [75], who found significant variation in tolerance to lodging and shattering across the 34 soybean genotypes evaluated. The root nodule score ranged from 2.17 to 3.61 with an average of 3.13. Among the 45 soybean genotypes, 27 nodulated more efficiently, and the best genotypes were G02, G16, G24, G29, G45, G01, G07, and G15. These results agree with those of Thio et al. [68] and Bello et al. [76], who reported different mean performances in root nodule scores. The plant height among the genotypes varied from 63.6 to 102 cm. The tallest plant height was recorded for G17, whereas the shortest was recorded on G02, which was also the top-performing genotype for grain yield. The results of this study is in line with the findings of Thio et al. [68] and Njoroge and Njeru [77], who reported plant height ranged from 51.1 cm to 102.8 cm and 60 cm to 109cm,

respectively. In contrast, Hizli et al. [78] reported up to 151.8cm plant height, which is taller than the heights found in this study.

Principal component analysis (PCA) aids in simplifying multidimensional data by breaking down its complexity into simpler principal components (PCs) by retaining important information [79, 80][48, 49]. The first three PCs with eigenvalues greater than one and accounted for about 64.8% of the total variation were considered important (Table 45). In a similar study on soybeans, Aondover et al. [74][50] reported that the first three principal components accounted for 76.4% of the total variation. Similarly, Verma et al. [81][51] reported that the first three PCs contributed 91.7% of the total variation. In their study, Akter [73][52] and Vijayakumar et al. [80][49] also reported that the first three PCs accounted for 77.7% and 73.7% of the total variation, respectively. Each PC is independently associated with the different yield and yield-related variables examined. The first PC that showed the highest contributions (27.2%) to the total variation was highly and positively associated with traits lodging, grain yield, and hundred seed weight, and highly and negatively associated with days to 95% maturity, and plant height, indicating the importance of these traits in the genetic improvement of the studied germplasm. The average contributions of RtNd, HSW, PH, D50F, and Lodg HSW, GY, RtNd, PH, and D50F were high in the principal axes. This finding implies that these traits account for the majority of the variations in soybeans and further influence the yield of soybeans. This finding aligns with the results of Aondover et al. [74][50], Adetiloye et al. [82][53], Sivabharathi et al. [83][54], Vianna et al. [84][55] and Leite et al. [85]Khan et al. [56], who reported the mean contributions of RtNd, HSW, PH, D50F, and Lodg HSW, GY, PH, and D50F were high in the principal axes. Thus, these traits must be considered when selecting grain yield. Soybean genotypes were spread over the biplot based on the first two PCs, also reflecting high variability in the studied germplasm. Genotypes G15, G24, G45, G13,

and G02 were far from the origin and identified as highly interactive and diverse genotypes; whereas G23, G43, G11, G07, G01, and G09 were located close to the origin and considered stable genotypes or less diverse genotypes (Fig 1). Comparable results were reported by Sivabharathi et al. [83][54], who performed principal component analysis on 135 soybean genotypes and reported genotypes MACS 1460, EC 18736, and PK 1038 as highly divergent genotypes, while JS 89-24, NRC 25, NRC 2007-G-1-13, NRC 43 and PK 7247 genotypes were less diverse and stable.

The studied genotypes were grouped into five distinct clusters, which also indicates the divergent nature of these germplasm. This result was comparable to the findings of Getnet [66][57] and Ghiday and Sentayehu [86][58], who reported the clustering of 49 soybean genotypes into five and three distinct clusters, respectively. Similarly, Yirga et al.[71][59] performed cluster analysis of 100 soybean genotypes based on morphological traits that were grouped into five different clusters, which implied the presence of genetic variability among the tested genotypes. Adetiloye et al. [82][53] also reported the clustering of 43 accessions into nine distinct clusters. These findings indicate the studied soybean genotypes exhibited wide genetic diversity. Utilizing genetically diverse parents in hybridization programs is critical in crop improvement activities [73][52] in ensuring high genetic recombinations in the progeny populations. Considering the cluster mean (Table 56), the genotypes in cluster IV showed the highest performances for grain yield and 100 seed weight, while the genotypes included in cluster II might be the preferred cluster to improve the crop for early flowering and maturity, and cluster I for high nodulation. The present outcome supports prior findings [87-90][56, 60-62].

In this study, the observed heterozygosity (Ho) of 0.08 was lower than the expected heterozygosity of the genotypes, possibly due to soybeans' high self-pollinating nature [91][63]. This implies high possibilities of inbreeding and fixation at most loci [92, 93][64, 65]. The mean observed

heterozygosity of 0.08 obtained in this study is higher compared to the 0.02 and 0.06 reported by Tsindi et al. [35][28] and Chiemeke et al. [94][66], respectively, in soybeans using SNP markers. However, it is lower compared to the 0.22 stated by Yohane et al. [95][67] when assessing 81 pigeon pea genotypes based on 4122 SNP markers, but similar to the results of Gumede et al. [59][47], when assessing 90 cowpea accessions using 5864 SNP markers. The average expected heterozygosity (He = 0.27) found in the current study was lower than the He of 0.31 reported by Abebe et al. [34][27] and Tsindi et al. [35][28] evaluating 65 and 210 soybean genotypes using 1223 and 403 SNP markers, respectively. The minor allele frequency (MAF) values measure the ability of markers to discriminate among genotypes [35][28]. Minor allele frequency (MAF) varied from 0.01 to 0.50 with an average of 0.20. The average MAF value found in this study was greater than the 0.24 reported by Tsindi et al. [35][28] based on the 403 SNPs in 210 soybean genotypes, 0.25 reported by Naflath et al. [96][68] based on the 29,955 SNPs in 96 soybean genotypes and 0.32 reported by Chander et al. [97][69] based on 186 SNPs in 155 soybean lines. Dube et al. [40][33] found similar results in maize with the MAF ranging from 0.01 to 0.5. A mean PIC value of 0.22 indicates that the markers were informative. A mean PIC value of 0.22 in the current study was lower than the PIC values reported in various crops such as 0.25 in soybean [34][27], 0.31 in maize [40][33], 0.36 in wing yam [41][34], 0.29 in wheat [98][70], 0.27 in cowpea [59][47], 0.26 in pea [99][71]. However, it is comparable to the results in other legumes i.e., 0.22 in common bean [100] [72] and 0.23 in cowpea [101][73].

The observed (Ho = 0.08) and expected (He = 0.27) heterozygosity values in this study reflect soybean's self-pollinating nature, which naturally results in lower heterozygosity [91, 102]. However, the level of expected heterozygosity still indicates the presence of useful genetic variation across the population [103]. This moderate diversity is essential for breeders because it

enables effective selection of genotypes with favorable traits [59, 104]. The disparity between Ho and He also suggests some degree of inbreeding and allele fixation, highlighting the need for incorporating genetically diverse parents in the crossing program to maintain and further increase the genetic base of IITA soybean [92, 97, 104]. Besides, by comparing Ho and He to other soybean germplasm such as Abebe et al. [34], Tsindi et al. [35], and Chiemeke et al. [94], breeders can pinpoint the most diverse parents for hybridization and thus maximize recombination of useful alleles [105, 106]. Additionally, moderate average values of MAF (0.20) and PIC (0.22) confirm that the SNP markers used are informative enough to distinguish genotypes and support population structure analysis [35, 54, 97]. These diversity indices together provide valuable guidance for parental selection, germplasm conservation, and marker deployment in soybean improvement programs [54, 105].

Population structure analysis is the process of determining a breeding line's ancestry using genotypic data [38, 107]~~[31, 74]~~. The SNP analysis identified three sub-populations (K = 3) among the 45 soybean genotypes based on the optimal K=3 determined according to the BIC method, which was not consistent with the results of phenotypic clustering. The inconsistency might be that most of the phenotypic traits are controlled by polygenes, and environmental conditions highly influence these traits [108, 109]~~[75, 76]~~. In the same way, the phylogenetic tree and DAPC also showed three sub-populations with higher degrees of admixture. The high degree of admixture found suggests that there was either gene flow or that these subpopulations had a common ancestor [91]~~[63]~~. Chander et al. [97]~~[69]~~ found comparable amounts of admixture in their study of 155 soybean genotypes, predominantly IITA-bred soybean varieties. The overall results of the population structure analysis are in agreement with the results of Abebe et al. [34]~~[27]~~ and Fatokun

et al. [101][73], who identified three sub-populations among the 65 soybean genotypes and 298 cowpea accessions, respectively based on BIC and DAPC methods.

The analysis of genetic distances within clusters revealed considerable variation, reflecting the underlying genetic diversity among the genotypes. In cluster I, the greatest genetic distance was recorded between genotypes SY007 and SY024, suggesting substantial genetic differentiation between these two individuals. This could imply limited recent shared ancestry or contrasting selection pressures. On the other hand, the lowest genetic distance was observed between SY013 and SY012, indicating a closer genetic relationship, possibly due to recent common ancestry or shared breeding lineage [107, 110]. In cluster II, the widest genetic distance was found between genotypes SY026 and SY030, mirroring the divergence seen in Cluster 1. Such high differentiation might suggest that these genotypes belong to distinct subpopulations or have undergone different evolutionary pressures. Conversely, the minimal genetic distance in this cluster was surprisingly low between SY037 and SY029, indicating a near-identical genetic makeup. This could reflect either clonal propagation, recent hybridization, or strong genetic conservation between the two genotypes. Cluster III also exhibited a maximum genetic distance, again between SY030 and SY026, reinforcing the notion of pronounced genetic divergence between these two genotypes across clusters. The lowest genetic distance in this cluster was between SY026 and SY020, suggesting a close relationship and potential kinship or shared origin. This pattern of high and low pairwise distances within clusters indicates that while clustering helps group genetically similar individuals, considerable diversity still exists within each group, which is critical for maintaining adaptive potential and guiding breeding decisions. Various genetic distances within soybean germplasm have been observed in a similar study by [35, 37, 102, 111]

Conclusion

The combined analysis of variance revealed that there was a significant difference among genotypes, locations, and genotype ×location interaction for days to 95% maturity and grain yield, indicating genotypes exhibited different relative performance across locations for these traits. The genotypes G02, G10, G11, G01, and G24 were found to be the most high-yielding (3050-2990 kg/ha). Principal component analysis (PCA) showed that about 64.8% of the variation was explained by the first three principal components with eigenvalues greater than one. The average contributions of HSW, Lodg~~GY~~, RtNd, PH, and D50F were high in the principal axes, implying that these characters are the major contributors to the total variation and influence the yielding ability of the studied soybean genotypes. The cluster analysis resulting from the hierarchical ascending classification grouped the 45 soybean genotypes into five clusters. This suggests that there was moderate variation among the studied soybean genotypes. Based on cluster mean values, genotypes selected from distant clusters such as clusters II and IV were desirable for use as parents in future hybridization programs to develop high-yielding soybean varieties.

Concerning the markers used in the study, a total of 10,630 SNP markers were deployed to study the population structure of 45 soybean genotypes. The SNP markers were moderately informative, with a mean PIC value of 0.22 across all the genotypes. The observed and expected heterozygosity values were 0.08 and 0.27, respectively while the minor allele frequency ranged from 0.01 to 0.50 with a mean of 0.20. The Discriminant Analysis of Principal Component (DAPC) and Bayesian Information Criterion (BIC) method grouped the 45 soybean genotypes into three subpopulations. The current study suggested that the genotypes under investigation exhibited considerable genetic diversity.

Acknowledgments

The first author is grateful for the scholarship granted by the African Union Commission for her MSc studies at the Pan African University Life and Earth Sciences Institute (including Health and Agriculture), University of Ibadan, Nigeria. We would like to acknowledge the staff and technicians of the soybean breeding and biometric unit, at the International Institute of Tropical Agriculture for all their invaluable guidance.

References

[1] Berk Z. Technology of production of edible flours and protein products from soybean. *FAO Agric Serv Bull* 1992; 1–10.

[2] Yadava DK, Vasudev S, Singh N, Mohapatra T, Prabhu KV. Breeding major oil crops: Present status and future research needs. *Technol Innov major world oil Crop* 2012; Volume 1: 17–51.

[3] Wilson RF. Soybean: Market Driven Research Needs. In Genetics and Genomics of Soybean 2008; 3–15. New York, NY: Springer New York. https://doi.org/10.1007/978-0-387-72299-3_1.

[4] Malik MFA, Qureshi AS, Ashraf M, Ghafoor A. Genetic variability of the main yield related characters in soybean. *Int J Agric Biol* 2006; 8: 815-9.

[5] Hymowitz T, Shurtleff WR. Debunking soybean myths and legends in the historical and popular literature. *Crop Sci* 2005; 45: 473–6.

[6] FAOSTAT. A list of countries by soybean production from 2016 to 2020. *In Wikipedia. https://en.wikipedia.org/wiki/List_of_countries_by_soybean_production*.

[7] Akande SR, Oyekan PO and ADESOYE AI. Soybean in Nigeria: Introduction, production and utilization. *LEGUME* 2013; 16: 38.

[8] Chandrawat KS, Baige KS, Hashmi S, Sarang DH, Kumar A, Dumani PK. Study on

Genetic Variability, Heritability and Genetic Advance in Soybean. *Int J Pure Appl Biosci* 2017; 5(1): 57–63.

[9] Singer WM, Lee YC, Shea Z, Vieira CC, Lee D, Li X, et al. Soybean genetics, genomics, and breeding for improving nutritional value and reducing antinutritional traits in food and feed. Plant Genome 2023; 16(4): e20415.

[10] Zewudie KG, Gemede HF. Assessment of nutritional, antinutritional, antioxidant and functional properties of different soybean varieties: implications for soy milk development. Cogent Food Agric 2024; 10(1): 2380496.

[11] Joseph OO, Uzoma AC, Juliana AN, Precious S. Proximate and Anti-Nutritional Analyses of Three Soybeans (*Glycine max* (L.) Merrill). IMCC J Sci 2024; 4(1): 10–16.

[12] Kumar A, Ramesh KV, Singh C, Sripathy KV, Agarwal DK, Pal G, et al. Bioprospecting nutraceuticals from soybean (*Glycine max*) seed coats and cotyledons. Indian J Agric Sci 2019; 89(12): 2064–2068.

[13] Tamangwa MW, Djikeng FT, Feumba RD, Sylvia VT, Loungaing VD, Womeni HM. Nutritional composition, phytochemical, and functional properties of six soybean varieties cultivated in Cameroon. Legum Sci 2023; 5(4): e210.

[14] Nwosu DJ, Olubiyi MR, Aladele SE, Apuyor B, Okere AU, Lawal AI, et al. Proximate and mineral composition of selected soybean genotypes in Nigeria. J Plant Dev 2019; 26: 67–76.

[15] Fasusi SA, Kim JM, Kang S. Current status of soybean production in Nigeria: constraints and prospects. J Korean Soc Int Agric 2022; 34: 149–156.

[~~9~~16] Meena BL, Das SP, Meena SK, Kumari R, Devi AG, Devi HL. Assessment of GCV, PCV, Heritability and Genetic Advance for Yield and its Components in Field Pea (*Pisum sativum* L.). *Int J Curr Microbiol Appl Sci* 2017; 6(5): 1025–33.

[~~10~~17] Nkhoma N, Shimelis H, Laing MD, Shayanowako A, Mathew I. Assessing the genetic diversity of cowpea [*Vigna unguiculata* (L .) Walp.] germplasm collections using phenotypic traits and SNP markers. BMC genetics. 2020; 21: 1–16.

[~~11~~18] Singh S, Trivedi PKA, Koshal AK. Seed Protein Profiling through Electrophoresis in Mungbean [*Vigna Radiata* (L.) Wilczek]. *J Plant Dev Sci* 2015; 7(4): 337–40.

[~~12~~19] Chen Y, Nelson RL. Genetic Variation and Relationships among Cultivated Wild, and Semiwild Soybean. *Crop Sci* 2004; 44(1): 316–25.

[~~13~~20] Dayaman V, Senthil N, Raveendran M, Nadarajan N, Shanmugasundaram P, Balasubramanian P. Diversity analysis in selected Indian soybean [*Glycine max* (L.) Merrill] using morphological and SSR markers. *Int J Integr Biol* 2009; 5: 125–129.

[~~14~~21] Liu M, Zhang M, Jiang W, Sun G, Zhao H, Hu S. Genetic diversity of Shaanxi soybean landraces based on agronomic traits and SSR markers. *African J Biotechnol* 2011; 10(24): 4823–4837.

[~~15~~22] Malik MFA, Ashraf M, Qureshi AS, Khan MR. Investigation and comparison of some morphological traits of the soybean populations using cluster analysis. *Pakistan J Bot* 2011; 43(2): 1249–1255.

[~~16~~23] Matsuo É, Sediyama T, Cruz CD, Oliveira RDL, Oliveira RCT, Nogueira APO. Genetic diversity in soybean genotypes with resistance to Heterodera glycines. *Crop Breed Appl Biotechnol* 2011; 11: 304–12.

[~~17~~24] Salimi S, Lahiji HS, Abadi GM, Salimi S, Moradi S. Genetic diversity in Soybean genotypes under drought stress condition using factor analysis and cluster analysis. *World Appl Sci J* 2012; 16(4): 474–478.

[~~18~~25] Mushoriwa H. Breeding Gains, Diversity Analysis and Inheritance Studies on Soybean [*Glycine max* (L .) Merrill] Germplasm in Zimbabwe. 2013; 210.

[~~19~~26] Liu Z, Li H, Fan X, Huang W, Yang J, Wen Z, et al. Phenotypic characterization and genetic dissection of nine agronomic traits in Tokachi nagara and its derived cultivars in soybean (*Glycine max* (L.) Merr.). *Plant Sci* 2017; 256: 72–86.

[~~20~~27] Bairagi V, Mishra S, Sen R, Dixit S, Tyagi DB. Assessment of Genetic Variability in Soybean (*Glycine max* L., Merrill). *Biol Forum–An Int J* 2023; 15: 258–63.

[~~21~~28] Kuswantoro H, Adie MM, Putri PH. Genetic Variability, Heritability, and Genotypic

Correlation of Soybean Agronomic Characters. *Bul Palawija* 2021; 19(2): 117–25.

[~~22~~29] Marconato MB, Pereira EM, Silva FM, Bizari EH, Pinheiro JB, Mauro AO, et al. Genetic divergence in a soybean (*Glycine max*) diversity panel based on agro-morphological traits. *Genet Mol Res*.2016; 15:1-10. DOI: 10.4238/gmr15048980.

[~~23~~30] Bhandari HR, Bhanu AN, Srivastava K, Singh MN, Shreya HA. Assessment of Genetic Diversity in Crop Plants - An Overview. *Adv Plants Agric Res* 2017; 7(3): 279–86.

[~~24~~31] Ganal MW, Altmann T, Röder MS. SNP identification in crop plants. *Curr Opin Plant Biol* 2009; 12(2): 211–17.

[~~25~~32] Ren J, Sun D, Chen L, You FM, Wang J, Peng Y, et al. Genetic diversity revealed by single nucleotide polymorphism markers in a worldwide germplasm collection of durum wheat. *Int J Mol Sci* 2013; 14: 7061–88.

[~~26~~33] Prempeh RNA, Manu-aduening JA, Quain MD, Asante IK, Offei SK, Danquah EY. Assessment of genetic diversity among cassava landraces using single nucleotide polymorphic markers. *African J Biotechnol* 2020; 19(6): 383–91.

[~~27~~34] Abebe AT, Kolawole AO, Unachukwu N, Chigeza G, Tefera H, Gedil M. Assessment of diversity in tropical soybean (*Glycine max* (L.) Merr.) varieties and elite breeding lines using single nucleotide polymorphism markers. *Plant Genet Resour* 2021; 19(1): 20–28.

[~~28~~35] Tsindi A, Eleblu JSY, Gasura E, Mushoriwa H, Tongoona P, Danquah EY, et al. Analysis of population structure and genetic diversity in a Southern African soybean collection based on single nucleotide polymorphism markers. *CABI Agric Biosci* 2023; 4(1): 15.

[~~29~~36] Potapova NA, Zlobin AS, Perfil RN, Vasiliev GV, Salina EA, Tsepilov YA. Population Structure and Genetic Diversity of the 175 SoybeanBreeding Lines and Varieties Cultivated in West Siberia and Other Regions of Russia. *Plants* 2023;12(19):3490.

[~~30~~37] Andrijani Z, Nazzicari N, Šarcevic H, Sudarić A, Annicchiarico P, Pejić I. Genetic Diversity and Population Structure of European Soybean Germplasm Revealed by Single Nucleotide Polymorphism. *Plants* 2023; 12(9): 1837.

[3138] Adu GB, Badu-Apraku B, Akromah R, Garcia-Oliveira AL, Awuku FJ, Gedil M. Genetic diversity and population structure of early-maturing tropical maize inbred lines using SNP markers. *PLoS One* 2019; 14(4): https://doi.org/10.1371/journal.pone.0214810.

[3239] Badu-Apraku B, Garcia-Oliveira AL, Petroli CD, Hearne S, Adewale SA, Gedil M. Genetic diversity and population structure of early and extra-early maturing maize germplasm adapted to sub-Saharan Africa. *BMC Plant Biol* 2021; 21: 1–15.

[3340] Dube SP, Sibiya J, Kutu F. Genetic diversity and population structure of maize inbred lines using phenotypic traits and single nucleotide polymorphism (SNP) markers. *Sci Rep* 2023; 13(1): 17851.

[3441] Agre P, Asibe F, Darkwa K, Edemodu A, Bauchet G, Asiedu R, et al. Phenotypic and molecular assessment of genetic structure and diversity in a panel of winged yam (*Dioscorea alata*) clones and cultivars. *Sci Rep* 2019; 9(1): 18221.

[3542] Bhattacharjee R, Agre P, Bauchet G, De Koeyer D, Lopez-Montes A, Kumar PL, et al. Genotyping-by-sequencing to unlock genetic diversity and population structure in white yam (*Dioscorea rotundata* Poir.). *Agronomy* 2020; 10(9):1437. doi: 10.3390/agronomy10091437.

[3643] Agre PA, Dassou AG, Loko LEY, Idossou R, Dadonougbo E, Gbaguidi A, et al. Diversity of white Guinea yam (*Dioscorea rotundata* Poir.) cultivars from Benin as revealed by agro-morphological traits and SNP markers. *Plant Genet Resour* 2021; 19(5): 437–46.

[3744] Adewale BD, Ojo DK, Abberton M. GGE Biplot application for adaptability of African yam bean grain yield to four agroecologies in Nigeria. African Crop Sci J 2017; 25(3): 333–47.

[3845] Fatima UA, Mohammed MS, Oyekunle M, Abdulmalik MM, Usman A. Screening soybean (*Glycine Max* (L.) Merrill) genotypes for resistance to pod shattering in Zaria, Nigeria. *FUDMA J Sci* 2020; 4(1): 727–31.

[3946] Omoigui LO, Kamara AY, Kamai N, Dugje IY, Ekeleme F, Kumar P, et al. Guide to Soybean Production in Northern Nigeria. *International Institute of Tropical Agriculture,*

Nigeria. 2020.http://www.ajol.info/index.php/naj%0Awww.iita.org%0A

[4047] International Board for Plant Genetic Resources (IBPGR). Descriptors for Soybean. 1984.

[4148] R Development Core Team. R: A Language and Environment for Statistical Computing. R Foundation for Statistical Computing, Vienna, Austria, 2007. ISBN 3-900051-07-0, URL http: //www.R-project.org/.

[4249] Kilian A, Sanewski G, Ko L. The application of DArTseq technology to pineapple. InXXIX *International Horticultural Congress on Horticulture: Sustaining Lives, Livelihoods and Landscapes (IHC2014): IV 1111*. 2014, pp. 181-88.

[4350] Danecek P, Auton A, Abecasis G, Albers CA, Banks E, DePristo MA, et al. The variant call format and VCFtools. *Bioinformatics* 2011; 27(15): 2156–58.

[4451] Purcell S, Neale B, Todd-Brown K, Thomas L, Ferreira MA, Bender D, et al. PLINK: a tool set for whole-genome association and population-based linkage analyses. *Am J Hum Genet* 2007; 81(3): 559–75.

[52] Chesnokov YV, Artemyeva AM. Evaluation of the measure of polymorphism information of genetic diversity. Agric Biol 2015; 50(5): 571–578.

[53] Liu BH. Statistical genomics: linkage, mapping and QTL analysis. CRC Press Boca Raton, Florida, USA; 1998.

[54] Xue H, Yu X, Fu P, Liu B, Zhang S, Li J, et al. Construction of the Core Collection of *Catalpa fargesii f. duclouxii* (Huangxinzimu) Based on Molecular Markers and Phenotypic Traits. Forests 2021; 12(11): 1518.

[55] Amiryousefi A, Hyvönen J, Poczai P. iMEC: Online Marker Efficiency Calculator. Appl Plant Sci 2018; 6(6): e1159.

[4556] Jombart T. adegenet: a R package for the multivariate analysis of genetic markers. *Bioinformatics* 2008; 24(11): 1403–1405.

[57] Gower JC. A general coefficient of similarity and some of its properties. Biometrics 1971; 27(4): 857–871.

[~~46~~58] Ebert AW EJ. Plant biodiversity and genetic resources matter! *Plants* 2020; 9: 1706.

[~~47~~59] Gumede MT, Gerrano AS, Amelework AB, Modi AT. Analysis of Genetic Diversity and Population Structure of Cowpea (*Vigna unguiculata* (L.) Walp) Genotypes Using Single Nucleotide Polymorphism Markers. *Plants* 2022; 11(24): 3480. **https://doi.org/10.3390/plants11243480**.

[60] Tesfaye A, Githiri M, Derera J, Debele T. Genetic variability in soybean (*Glycine max* L.) for low soil phosphorus tolerance. Ethiop J Agric Sci 2017; 27(2): 1–15.

[61] Krisnawati A, Adie MM. Yield stability of soybean promising lines across environments. IOP Conf Ser Earth Environ Sci 2018; 102. DOI: 10.1088/1755-1315/102/1/012044.

[62] Pedro JA. Evaluation of soybean [*Glycine max* (L.) Merrill] Genotypes for grain yield and associated agronomic traits under low and high phosphorus environments. (Master thesis, University of KwaZulu-Natal Pietermaritzburg, South Africa. 2018.

[63] Njoroge JN, Owouche JO, Oyoo ME. Evaluation of soybean [*Glycine max* (L.) Merr.] genotypes for agronomic and quality traits in Kenya. African J Agric Res 2015; 10(12): 1474–1479.

[64] Nachilima C. Genotype plus genotype-by-environment interaction analysis of soybean [*Glycine max* (L. Merrill] across production environments in Southern Africa. (Masters thesis, The University of Zambia). 2021.

[65] Abebe AT, Adewumi AS, Adebayo MA, Shaahu A, Mushoriwa H, Alabi T, et al. Genotype x environment interaction and yield stability of soybean (*Glycine max* L.) genotypes in multi-environment trials (METs) in Nigeria. Heliyon 2024; 10: e38097.

[66] Getnet BE. Genetic Variability, Heritability and Expected Genetic Advance as Indices for Selection in Soybean [*Glycine max* (L.) Merrill] Varieties. Am J Life Sci 2018; 6(4): 52–56.

[67] Beyene D, Jalata Z, Geleta N. Exploring the Genetic Variability, Heritability and Genetic Gain in Soybean [*Glycine max* (L.) Merrill] Genotypes. Int J Agric Res 2022; 17(2): 43–49

[68] Thio IG, Ouedraogo N, Drabo I, Essem F, Neya FB, Sombie PAED, et al. Evaluation of

medium maturity group of soybean [*Glycine max* (L.) Merr] for agronomic performance and adaptation in Sudanian zone of Burkina Faso. African J Agric Res 2022; 18(4): 264–275.

[69] Sileshi Y. Estimation of Variability, Correlation and Path Analysis in Soybean [*Glycine max* (L.) Merr.] Genotypes at Jimma, South Western Ethiopia. J Nat Sci Res 2019; 9(7): 22–29.

[70] Goonde DB, Ayana NG. Genetic Diversity and Character Association for Yield and Yield Related Traits in Soybean (*Glycine max* L.) Genotypes. J Agric Sci Food Res 2021; 2(1): 1-5.

[71] Yirga M, Sileshi Y, Tesfaye A, Hailemariam M. Genetic Variability and Association of Traits in Soybean (*Glycine max* (L.) Genotypes in Ethiopia. Ethiop J Crop Sci 2022; 9(2): 49-74.

[72] Jandong EA, Uguru MI, Okechukwu EC. Estimates of genetic variability, heritability and genetic advance for agronomic and yield traits in soybean (*Glycine max* L.). African J Biotechnol 2020; 19(4): 201–206.

[73] Akter S. Character association and diversity analysis of different genotypes of soybean [*Glycine max* (L.) Merr.]. (Master thesis, Sher-e-Bangla Agricultural University, Dhaka-1207, Bangladesh). 2021.

[74] Aondover S, Lekan BL, Terkimbi V. Correlation, path coefficient and principal component analysis of seed yield in soybean genotypes. Int J Adv Res 2013; 1(7): 1–5.

[75] Antwi-boasiako A. Screening of soybean [*Glycine max* (L.) Merrill] genotypes for resistance to lodging and pod shattering. Int J Agron Agric Res 2017; 10(5): 1–8.

[76] Bello LL, Shaahu A, Vange T. Studies on relationship between seed yield and yield components in Soybean [*Glycine max* (L.) Merrill]. *Electron J Plant Breed* 2012; 3(4): 1012–1017.

[77] Njoroge JN, Njeru PNM. Performance of soybean genotypes evaluated for yield and protein content in Nakuru County, Kenya. Joint Proceedings of the 27th Soil Science Society of East Africa and the 6th African Soil Science Society Conference. 2013: 255–

261.

[78] Hizli H, Cubukcu P, Sahar AK. Path Analysis, Genetic Variability and Correlation Studies of Related Characters for Forage Soybean [*Glycine max* (L.) Merrill]. O*smaniye Korkut Ata Univ Inst J Sci Technol* 2023; 6(2): 1513–1528.

[~~48~~79] Jollife IT, Cadima J. Principal component analysis: A review and recent developments. *Philos Trans R Soc A Math Phys Eng Sci* 2016; 374(2065). DOI: https://doi.org/10.1098/rsta.2015.0202.

[~~49~~80] Vijayakumar E, Sudhagar R, Vanniarajan C, Ramalingam J, Allan V, Senthil N. Estimating the Breeding Potency of a Soybean Core Set. *Int J Agric Biol* 2022; 27(3): 184–96.

~~[50] Aondover S, Lekan BL, Terkimbi V. Correlation, path coefficient and principal component analysis of seed yield in soybean genotypes. *Int J Adv Res* 2013; 1(7): 1–5.~~

[~~51~~81] Verma V, Shrivastava MK, Mehra S, Amrate PK, Yadav RB. Estimation of Genetic Parameters for Yield Associated Traits and Principal Component in Advance Breeding Lines of Soybean (*Glycine max*. (L.) Merrill). *Int J Curr Microbiol Appl Sci* 2021; 10(1): 2704–2710.

~~[52] Akter S. Character association and diversity analysis of different genotypes of soybean (*Glycine max* L Merr.). Doctoral dissertation, Sher-E-Bangla Agricultural University. 2021.~~

[~~53~~82] Adetiloye IS, Ariyo OJ, Alamu O, Osewa SO. Agronomic Potential and Genetic Diversity of 43 Accession of Tropical Soybean (*Glycine max* (L) Merrill). *Int J Plant Res* 2020; 10: 33–39. doi**: 10.5923/j.plant.20201002.02** .

[~~54~~83] Sivabharathi RC, Muthuswamy A, Anandhi K, Karthiba L. Genetic Diversity Studies of Soybean [*Glycine max* (L.) Merrill] Germplasm Accessions using Cluster and Principal Component Analysis. *Legum Res - an Int J* 2023; 1: 1–6.

[~~55~~84] Vianna VF, Unêda-Trevisoli SH, Desidério JA, Santiago SD, Charnai K, Ferreira Júnior JA, et al. The multivariate approach and influence of characters in selecting superior soybean genotypes. *African J Agric Res* 2013; 8(30): 4162–69. doi:

10.5897/AJAR2013.7064.

~~[56] Khan MSA, Karim MA, Haque MM, Karim AJM, Mian MAK. Variations in agronomic traits of Soybean genotypes. *SAARC J Agric* 2014; 12(2): 90–100.~~

[85] Leite WS, Unêda-Trevisoli SH, Silva FM, Silva AJ, Di Mauro AO. Identification of superior genotypes and soybean traits by multivariate analysis and selection index. Rev Cienc Agron 2018; 49(3): 491–500.

~~[57] Getnet BE. Genetic Variability, Heritability, and Expected Genetic Advance as Indices for Selection in Soybean [*Glycine max* (L.) Merrill] Varieties. *Am J Life Sci* 2018; 6(4): 52–56. doi: 10.11648/j.ajls.20180604.11.~~

[~~58]~~86 Ghiday T, Sentayehu A. Genetic Divergence Analysis on Some Soybean [*Glycine max* L . Merrill] Genotypes Grown in Pawe, Ethiopia. *Am J Agric Environ Sci* 2015; 15(10): 1927–33, DOI: 10.5829/idosi.aejaes.2015.15.10.12777.

[87] Khan MSA, Karim MA, Haque MM, Karim AJM, Mian MAK. Variations in agronomic traits of Soybean genotypes. *SAARC J Agric* 2014; 12(2): 90–100.

~~[59] Yirga M, Sileshi Y, Tesfaye A, Hailemariam M. Genetic Variability and Association of Traits in Soybean (*Glycine max* (L.) Genotypes in Ethiopia. *Ethiop J Crop Sci* 2022; 9(2): 49–74.~~

[~~60~~88] Alina MM, Kingstone M. Genetic Differentiation of ARC Soybean [*Glycine Max* (L.) Merrill] Accessions Based on Agronomic and Nutritional Quality Traits. *Agric Food Sci Res* 2018; 5: 6–22.

[~~61]~~89 El-Hashash EF. Genetic diversity of soybean yield based on cluster and principal component analyses. *J Adv Biol & Biotechnol* 2016; 10(3): 1–9.

[~~62~~90] Mahbub MM, Rahman MM, Hossain MS, Nahar L, Shirazy BJ. Morphophysiological variation in soybean (*Glycine max* (L.) Merrill). *Am J Agric Environ Sci* 2016; 16(2): 234–38, DOI: 10.5829/idosi.aejaes.2016.16.2.12687.

[~~63~~91] Liu YL, Li YH, Zhou GA, Uzokwe N, Chang RZ, Chen SY, et al. Development of soybean EST-SSR markers and their use to assess genetic diversity in the Subgenus soja.

Agric Sci china 2010; 9(10): 1423–1429. https://doi.org/10.1016/S1671-2927(09)60233-9.

[6492] Gupta, S. K., & Manjaya JG. Genetic diversity and population structure of Indian soybean [*Glycine max* (L.) Merr.] revealed by simple sequence repeat markers. *J Crop Sci Biotech* 2017; 20(3): 221–231.

[6593] Nawaz MA, Lin X, Chan TF, Lam HM, Baloch FS, Ali MA, et al. Genetic architecture of wild soybean (*Glycine soja Sieb*. and *Zucc*.) populations originating from different East Asian regions. *Genet Resour Crop Evol* 2021; 68: 1577-88.

[6694] Chiemeke FK, Olasanmi B, Agre PA, Mushoriwa H, Chigeza G, Abebe AT. Genetic Diversity and Population Structure Analysis of Soybean [*Glycine max* (L.) Merrill] Genotypes Using Agro-Morphological Traits and SNP Markers. *Genes (Basel)* 2024; 15: 1373.

[6795] Yohane EN, Shimelis H, Laing M, Shayanowako A. Genetic diversity and grouping of pigeon pea [*Cajanus cajan* Millspaugh] Germplasm using SNP markers and agronomic traits. *PLoS One* 2022; 17(11). https://doi.org/10.1371/journal.pone.0275060

[6896] Naflath TV, Rajendra PS RR. Population structure and genetic diversity characterization of soybean for seed longevity. *PLoS One* 2022; 17: e0278631.https://doi.org/10.1371/journal.pone.0278631

[6997] Chander S, Garcia-Oliveira AL, Gedil M, Shah T, Otusanya GO, Asiedu R et al. Genetic diversity and population structure of soybean lines adapted to Sub-Saharan Africa using single nucleotide polymorphism (SNP) markers. *Agronomy* 2021; 11(3): 604. **https://doi.org/10.3390/agronomy11030604**

[7098] Kumar D, Chhokar V, Sheoran S, Singh R, Sharma P, Jaiswal S, et al. Characterization of genetic diversity and population structure in wheat using array-based SNP markers. *Mol Biol Rep* 2020; 47: 293–306.

[7199] Brhane H, Hammenhag C. Genetic diversity and population structure analysis of a diverse panel of peas (*Pisum sativum*). *Front Genet* 2024; 15: 1396888. https://doi.org/10.3389/fgene.2024.1396888.

[72100] Nkhata W, Shimelis H, Melis R, Chirwa R, Mzengeza T, Mathew I, et al.

Population structure and genetic diversity analyses of common bean germplasm collections of East and Southern Africa using morphological traits and high-density SNP markers. *PLoS One* 2020; 15(2): e0243238. https://doi.org/10.1371/journal.pone.0243238.

[~~73~~101] Fatokun C, Girma G, Abberton M, Gedil M, Unachukwu N, Oyatomi O, et al. Genetic diversity and population structure of a mini-core subset from the world cowpea (*Vigna unguiculata* (L.) Walp.) germplasm collection. *Sci Rep* 2018; 8(1): 16035.

[102] Rani R, Raza G, Tung MH, Rizwan M, Ashfaq H, Shimelis H, et al. Genetic diversity and population structure analysis in cultivated soybean [*Glycine max* (L.) Merr.] using SSR and EST-SSR markers. PLoS One 2023; 18(5): e0286099.

[103] Yirgu M, Kebede M, Feyissa T, Lakew B, Woldeyohannes AB, Fikere M. Single nucleotide polymorphism (SNP) markers for genetic diversity and population structure study in Ethiopian barley (*Hordeum vulgare* L.) germplasm. *BMC Genomic Data* 2023; 24(1): 7.

[104] Sodedji FA, Agbahoungba S, Agoyi EE, Kafoutchoni MK, Choi J, Nguetta SP, et al. Diversity, population structure, and linkage disequilibrium among cowpea accessions. *The Plant Genome* 2021; 14(3): e20113.

[105] Carpentieri-Pípolo V, Pípolo AE, Silva FA, Petek MR. Soybean parent selection based on genetic diversity. *Brazilian Arch Biol Technol* 2000; 43: 295–300.

[106] Miller MJ, Song Q, Fallen B, Li Z. Genomic prediction of optimal cross combinations to accelerate genetic improvement of soybean (*Glycine max*). *Front Plant Sci* 2023; 14: 1171135.

[~~74~~107] Lawson DJ, Falush D. Population identification using genetic data. *Annu Rev Genomics Hum Genet* 2012; 13(1): 337–61. **https://doi.org/10.1146/annurev-genom-082410-101510**

[~~75~~108] Pham T. Analyses of genetic diversity and desirable traits in sesame *(Sesamum indicum* L., Pedaliaceae) implication for breeding and conservation, Master's theses, Swedish University of Agricultural Sciences. 2011.

[~~76~~109] Dey SS, Singh AK, Chandel D BT. Genetic diversity of bitter gourd (*Momordica charantia* L.) genotypes revealed by RAPD markers and agronomic traits. *Sci Hortic (Amsterdam)* 2006; 109: 21–28.

[110] Uba CU, Oselebe HO, Tesfaye AA, Abtew WG. Genetic diversity and population structure analysis of bambara groundnut (*Vigna subterrenea* L) landraces using DArT SNP markers. *PLoS One* 2021; 16(7): e0253600.

[111] Fu YB, Cober ER, Morrison MJ, Marsolais F, Peterson GW, Horbach C. Patterns of genetic variation in a soybean germplasm collection as characterized with genotyping-by-sequencing. *Plants* 2021; 10(8): 1611.

Supporting information

S1 Table. Eigenvalues, proportion of variance, and cumulative proportion of the studied soybean genotypes for eight agronomic and yield traits. D50F = days to 50% flowering, SHS = shattering score, RtNd = root nodule score, Lodg = lodging score, PH = plant height (cm), D95M = days to 95% maturity, HSW = hundred seed weight (gm), GY = grain yield (kg/ha).

S2 Table. List of genotypes and their grouping based on 10,630 SNP markers using the 70% cut-off criterion of the membership probability threshold.

S3 Table. Mean performances of the phenotypic traits of the study genotypes

S4 Table. Genetic distance matrix of the 45 soybean genotypes based on SNP markers.

S1 Fig. Summary statistics of 10,630 single nucleotide polymorphism (SNP) markers used for genotyping 45 soybean genotypes.

Rebuttal letter

Dear Editor and reviewers,

We would like to express our deepest gratitude to you for taking the time to review our manuscript and providing us with very constructive comments. We truly appreciate it! Below, we provide a detailed point-by-point response to the comments raised by the editor and the reviewers. All suggested revisions have been incorporated into the manuscript.

Editor comments:

As both reviewers recommended to add the ANOVA table and mean comparison for traits and also testing the data for homogeneity and normality, please follow such important concerns and modify the text based on the twos reviewers. Also, add a point by point response to reviewer letter when revising the manuscript

Response: Thank you for the valuable comment. Accordingly, we have added the ANOVA table and mean comparison results for the phenotypic traits (see new Table 4 and **S3** Table in the Results section). Although we did not conduct testing on the data for homogeneity and normality before the ANOVA analysis because the fitted linear mixed-effect model (using the lmerTest package in R) is robust to violations of both homogeneity of variance and normality. This model can explicitly handle heteroscedasticity through the inclusion of random effects and effectively handles deviations from normality, thus ensuring the validity and reliability of our results without requiring separate assumption testing.

Reviewer #1 Comments:

Line 39: Replace this "Soybean [Glycine max (L.) Merrill], is the leading oil seed crop, accounting for 57% of the global oil production." With this "Soybean [Glycine max (L.) Merrill] is one of the leading oilseed crops globally, accounting for approximately 57% of global vegetable oil production."

Response: The sentence has been revised accordingly in the Introduction.

Line 41-43: Replace this "It is cultivated in many countries and is a major source of vegetable oil and protein used in food, animal feed, and for industrial purposes." With this "It is cultivated in numerous countries and serves as a major source of vegetable oil and protein, essential in food, feed, and various industrial applications."

Response: The sentence has been updated as suggested.

Line 44-45: Replace this "It was domesticated in the eleventh (11th) century BC around Northeast China and was then transported throughout Asia, the USA, Brazil, and Argentina." With this "Soybeans were domesticated around the 11th century BC in Northeast China and subsequently spread across Asia, the USA, Brazil, and Argentina."

Response: The sentence has been updated as recommended.

Line 46-47: Replace this "Nigeria is the second-largest producer of soybeans in Africa, after South Africa. The first soybeans were brought into Nigeria in 1908, but the first successful cultivation was in 1937 with the Malayan variety, which was reported as commercial production in Benue State." With this "Nigeria ranks as the second-largest producer of soybeans in Africa, following South Africa. Soybeans were first introduced to Nigeria in 1908, with successful commercial cultivation beginning in 1937, using the Malayan variety in Benue State."

Response: The updated version has been incorporated into the Introduction.

Line 49-50: Replace this "The crop is adapted to various environmental conditions and is primarily cultivated under rain-fed conditions." With this "The crop is adapted to diverse environmental conditions and is predominantly grown under rain-fed conditions."

Response: The sentence has been revised as suggested.

Lines 55–57: How can the genetic improvement of soybeans be specifically tailored to address malnutrition in Nigeria or similar regions? Are there particular traits being targeted that would make soybeans more beneficial nutritionally?

Response: Thank you for this insightful question. We have now added a paragraph elaborating on how soybean breeding can address malnutrition through improving protein content and fatty acid profiles and reducing anti-nutritional factors (see revised Introduction, lines 65–75). Recent literature has been cited to support these claims.

Line 184: Have you considered including an Analysis of Variance (ANOVA) along with mean comparison for the phenotypic traits assessed before performing the multivariate statistical analyses such as PCA and clustering? This could help to highlight significant phenotypic variation among the genotypes and provide a clearer basis for the subsequent analyses.

Response: Yes, we have now included the ANOVA table and mean comparisons (see **Table 4** and **S3** Table). This provides a statistical basis for phenotypic variability and supports our multivariate analyses.

Line 259: The discussion section could benefit from incorporating the results from the ANOVA and mean comparison analyses, especially when interpreting the genetic variability observed in the study.

Response: We have now incorporated relevant ANOVA and mean comparison insights into the Discussion to better explain the observed genetic variability (see revised Discussion section, line 355–388).

Reviewer #2 Comments:

1. The results of ANOVA are needed.

Response: ANOVA results are now presented in Table 4 in the Results section and referenced in both the Results and Discussion.

2. Add the number of plants used for phenotypical measurements.

Response: The number of plants used for phenotypic evaluation has been added to the Materials and Methods section (see lines 153–156).

3. Add the method of cluster analysis.

Response: The method used for cluster analysis (the Euclidean distance and Ward's D^2 method) has been added to the Materials and Methods section (line 168).

4. Write the formulas used for parameter estimation.

Response: Formulas for observed heterozygosity (Ho), expected heterozygosity (He), minor allele frequency (MAF), and polymorphic information content (PIC) have been added to the Materials and Methods section, with relevant citations (see lines 208–222).

5. Consider eigenvectors equal to or greater than 0.4 as high in the PCA analysis and reinterpret.

Response: PCA results have been reinterpreted based on a 0.4 eigenvector threshold, and this is reflected in the revised Results and Discussion sections (see text revisions on lines 271–278 and 399-407).

6. Calculation of genetic distances between cluster groups and genotypes is required.

Response: We have calculated the genetic distances among genotypes and between clusters. These values are now presented in a supplementary table (Supplementary Table 4) and summarized in the Results section (lines 344–351).

7. I have mentioned some suggestions in the text of the article that should be corrected.

Response: All in-text suggestions and tracked comments provided by the reviewer have been addressed. The revised manuscript includes these corrections (see tracked changes file).

We thank you once again for your consideration and remain at your disposal for any further clarifications.

Sincerely,
Abebawork Tilahun Assfaw on behalf of all co-authors
