# Supplementary material for: Genetic diversity and population structure analysis of soybean [Glycine max (L.) Merrill] genotypes based on agro-morphological traits and SNP markers
Source: PLoS One. 2025 Oct 10;20(10):e0332895. doi: 10.1371/journal.pone.0332895 (PMC12513657; doi:10.1371/journal.pone.0332895)
Supplement: Attachment — Submitted filename: Response to Reviewers.docx [file pone.0332895.s008.docx]

**Rebuttal letter**

Dear Editor and reviewers,

We would like to express our deepest gratitude to you for taking the time to review our manuscript and providing us with very constructive comments. We truly appreciate it! Below, we provide a detailed point-by-point response to the comments raised by the editor and the reviewers. All suggested revisions have been incorporated into the manuscript.

**Editor comments:**

As both reviewers recommended to add the ANOVA table and mean comparison for traits and also testing the data for homogeneity and normality, please follow such important concerns and modify the text based on the twos reviewers. Also, add a point by point response to reviewer letter when revising the manuscript

**Response**: Thank you for the valuable comment. Accordingly, we have added the ANOVA table and mean comparison results for the phenotypic traits (see new Table 4 and **S3** Table in the Results section). Although we did not conduct testing on the data for homogeneity and normality before the ANOVA analysis because the fitted linear mixed-effect model (using the lmerTest package in R) is robust to violations of both homogeneity of variance and normality. This model can explicitly handle heteroscedasticity through the inclusion of random effects and effectively handles deviations from normality, thus ensuring the validity and reliability of our results without requiring separate assumption testing.

**Reviewer #1 Comments:**

Line 39: Replace this "Soybean [Glycine max (L.) Merrill], is the leading oil seed crop, accounting for 57% of the global oil production." With this "Soybean [Glycine max (L.) Merrill] is one of the leading oilseed crops globally, accounting for approximately 57% of global vegetable oil production."

**Response:** The sentence has been revised accordingly in the Introduction.

Line 41-43: Replace this "It is cultivated in many countries and is a major source of vegetable oil and protein used in food, animal feed, and for industrial purposes." With this "It is cultivated in numerous countries and serves as a major source of vegetable oil and protein, essential in food, feed, and various industrial applications."

**Response:** The sentence has been updated as suggested.

Line 44-45: Replace this "It was domesticated in the eleventh (11th) century BC around Northeast China and was then transported throughout Asia, the USA, Brazil, and Argentina." With this "Soybeans were domesticated around the 11th century BC in Northeast China and subsequently spread across Asia, the USA, Brazil, and Argentina."

**Response:** The sentence has been updated as recommended.

Line 46-47: Replace this "Nigeria is the second-largest producer of soybeans in Africa, after South Africa. The first soybeans were brought into Nigeria in 1908, but the first successful cultivation was in 1937 with the Malayan variety, which was reported as commercial production in Benue State." With this "Nigeria ranks as the second-largest producer of soybeans in Africa, following South Africa. Soybeans were first introduced to Nigeria in 1908, with successful commercial cultivation beginning in 1937, using the Malayan variety in Benue State."

**Response:** The updated version has been incorporated into the Introduction.

Line 49-50: Replace this "The crop is adapted to various environmental conditions and is primarily cultivated under rain-fed conditions." With this "The crop is adapted to diverse environmental conditions and is predominantly grown under rain-fed conditions."

**Response:** The sentence has been revised as suggested.

Lines 55–57: How can the genetic improvement of soybeans be specifically tailored to address malnutrition in Nigeria or similar regions? Are there particular traits being targeted that would make soybeans more beneficial nutritionally?

**Response:** Thank you for this insightful question. We have now added a paragraph elaborating on how soybean breeding can address malnutrition through improving protein content and fatty acid profiles and reducing anti-nutritional factors (see revised Introduction, lines 65–75). Recent literature has been cited to support these claims.

Line 184: Have you considered including an Analysis of Variance (ANOVA) along with mean comparison for the phenotypic traits assessed before performing the multivariate statistical analyses such as PCA and clustering? This could help to highlight significant phenotypic variation among the genotypes and provide a clearer basis for the subsequent analyses.

**Response:** Yes, we have now included the ANOVA table and mean comparisons (see **Table 4** and **S3** Table). This provides a statistical basis for phenotypic variability and supports our multivariate analyses.

Line 259: The discussion section could benefit from incorporating the results from the ANOVA and mean comparison analyses, especially when interpreting the genetic variability observed in the study.

**Response:** We have now incorporated relevant ANOVA and mean comparison insights into the Discussion to better explain the observed genetic variability (see revised Discussion section, line 355–388).

**Reviewer #2 Comments:**

1. The results of ANOVA are needed.

**Response**: ANOVA results are now presented in Table 4 in the Results section and referenced in both the Results and Discussion.

2. Add the number of plants used for phenotypical measurements.

**Response**: The number of plants used for phenotypic evaluation has been added to the Materials and Methods section (see lines 153–156).

3. Add the method of cluster analysis.

**Response**: The method used for cluster analysis (the Euclidean distance and Ward's D² method) has been added to the Materials and Methods section (line 168).

4. Write the formulas used for parameter estimation.

**Response**: Formulas for observed heterozygosity (Ho), expected heterozygosity (He), minor allele frequency (MAF), and polymorphic information content (PIC) have been added to the Materials and Methods section, with relevant citations (see lines 208–222).

5. Consider eigenvectors equal to or greater than 0.4 as high in the PCA analysis and reinterpret.

**Response**: PCA results have been reinterpreted based on a 0.4 eigenvector threshold, and this is reflected in the revised Results and Discussion sections (see text revisions on lines 271–278 and 399-407).

6. Calculation of genetic distances between cluster groups and genotypes is required.

**Response:** We have calculated the genetic distances among genotypes and between clusters. These values are now presented in a supplementary table (Supplementary Table 4) and summarized in the Results section (lines 344–351).

7. I have mentioned some suggestions in the text of the article that should be corrected.

**Response**: All in-text suggestions and tracked comments provided by the reviewer have been addressed. The revised manuscript includes these corrections (see tracked changes file).

We thank you once again for your consideration and remain at your disposal for any further clarifications.

Sincerely,
Abebawork Tilahun Assfaw on behalf of all co-authors
