# Supplementary material for: Genetic diversity and population structure analysis of soybean [Glycine max (L.) Merrill] genotypes based on agro-morphological traits and SNP markers
Source: PLoS One. 2025 Oct 10;20(10):e0332895. doi: 10.1371/journal.pone.0332895 (PMC12513657; doi:10.1371/journal.pone.0332895)
Supplement: Attachment — Submitted filename: PLOSOne Reviewer.docx [file pone.0332895.s010.docx]

Reviewer’s Comments

The authors reported on “Genetic diversity and population structure analysis of soybean [Glycine max (L.) Merrill] genotypes based on agro-morphological traits and SNP markers”, an aspect that is key to the success of every breeding program as a perquisite to successful varietal development. The authors have done well in the presentation of the manuscript sections though with minor corrections to improve the manuscript quality. My few comments for each section are below.

**Abstract**

L24&235: “…genotypes were 25 further assessed for SNPs-based genotypic diversity analyses”. Please include the type of marker and the quantity.

L28: take out the word “corresponding” the markers are not corresponding to the traits used for pheno-based phylogeny

L29 and 30: it is better to list a few of the desirable traits in the genotypes rather than just using a general perception.

L33: you said “considerable genetic variation” but concluded with “high genetic variation” which one should the reader accept?

**Introduction**

L53: I think you meant “…..high-yielding, climate, and stress-resilient…..”

**Materials and Methods**

L115 – 122: Experimental materials should come before experimental design and management.

L130: should be “…agro-morphological traits including plant height…”

L134 – 145: I assume you did ANOVA but chose not to report it. If no please it should be part of your results or at least in your supplementary, file but reported in your main text. ANOVA is the basis for establishing variability that is your diversity per se. you have data from three locations, readers will like to see environment, genotype, and their interaction effects prior to be interested in other results. Please include the process in handling these aspects of the analysis in the phenotypic data analysis focusing on choice of model used, justification, and how you generated the data for cluster analysis. You did cluster analysis but you kept information on the data used (BLUES or BLUP) and why?

L135: what do you mean by similarity coefficient? Did you use similarity matrix or dissimilarity matrix for clustering? In my opinion, your focus is diversity so dissimilarity matrix like, Gower and Euclidean are fine.

L143: You did PCA with two packages. I am familiar with both packages and can tell that both of them do not do PCA. Only one does the PCA while the other is for visualization. So please be specific which one was used for PCA and which was for visualization.

L149: what do you mean immature? I assume you meant fully expanded but young leaf. If so, please correct and if not please clarify.

L170 – 183: your MAF threshold seems very low (0.01), the standard is usually 0.05. Do you have a reason for bringing it down to 0.01? how did you handle markers with high heterozygosity? Did you check for call rate prior to QC and what was your threshold?

L177 and 178: You used both VCFtool and PLINK for filtering and marker diversity indices assessment. Please be clear which one is for which activity? BIC is not to estimate but to define optimum subpopulation.

**Results**

NB: Please include the proposed analysis in the materials and methods in the results section.

L185 – 196: You cited table 4 before fig 1. Please arrange the results in same manner.

L210: fig 2 should be placed after the text just before table 5.

L212 – 215: there is grammar issue here, please attend to it.

L220: Please subject the cluster performance to f-statistics to see whether the cluster groups are significantly different from one another for each of the traits. That way, you can identify which clusters actually possess what traits with statistical confidence.

L237: Your BIC vs cluster number graph had a sharp drop from one to two and at this point was also the lowest indicating that two was the optimal subpopulation but you said three. Could you justify? I suggest you verify the subpopulation with alternative analysis like PCA scree plot. If it happens that you consider two subpopulations then DAPC may have to change.

L249: Fig 5 seems to have been misplaced. The figure was titled phylogenetic three but was placed under population structure. If I am wrong then please provide the results for the phylogenetic analysis. This can be compared to the phylogenetic analysis from the phenotypic data to see how the genotypes grouping aligns.

**Discussion**

L313: The authors said “The minor allele frequency (MAF) values measure the ability of markers to discriminate among genotypes” this is new and contrary to what is known about MAF. If MAF does this, then what is function of PIC?

**Conclusion**

The conclusion is too long and needs to be reduced and more concise.

**Other information**

The figures not easily readable.
