# Supplementary material for: Genetic diversity and population structure analysis of soybean [Glycine max (L.) Merrill] genotypes based on agro-morphological traits and SNP markers
Source: PLoS One. 2025 Oct 10;20(10):e0332895. doi: 10.1371/journal.pone.0332895 (PMC12513657; doi:10.1371/journal.pone.0332895)
Supplement: Attachment — Submitted filename: Response_to_Reviewers_auresp_2.docx [file pone.0332895.s011.docx]

Rebuttal letter

Dear Editor and reviewers,

We would like to express our deepest gratitude to you for taking the time to review our manuscript and providing us with very constructive comments. We truly appreciate it! Below, we provide a detailed point-by-point response to the comments raised by the reviewers. All suggested revisions have been incorporated into the manuscript.

**Reviewer #1**

L24&235: “…genotypes were 25 further assessed for SNPs-based genotypic diversity analyses”. Please include the type of marker and the quantity.

**Response**: Revised to read: “…the genotypes were further assessed using 10,630 SNP markers obtained from DArTseq genotyping.

L28: take out the word “corresponding” the markers are not corresponding to the traits used for pheno-based phylogeny

**Response**: The word “corresponding” has been removed for clarity.

L29 and 30: it is better to list a few of the desirable traits in the genotypes rather than just using a general perception.

**Response**: Thank you for the suggestion. We have revised the sentence in the abstract to include specific desirable traits. The updated sentence now highlights traits such as grain yield, days to 50% flowering and days to 95% maturity, hundred seed weight, lodging, plant height, and nodulation, which were key in differentiating the genotypes.

L33: you said “considerable genetic variation” but concluded with “high genetic variation” which one should the reader accept?

**Response**: Thank you for the insightful comment. The term “considerable” was retained for consistency and accuracy in describing the level of genetic diversity observed. The concluding sentence was removed to avoid redundancy and potential confusion.

L53: I think you meant “…..high-yielding, climate, and stress-resilient…..”

**Response:** The sentence has been updated as suggested.

L115 – 122: Experimental materials should come before experimental design and management.

**Response:** The Experimental Materials section has been moved before Experimental Design and Management.

L130: should be “…agro-morphological traits including plant height…”

**Response:** The sentence has been updated as recommended.

L134 – 145: I assume you did ANOVA but chose not to report it. If no please it should be part of your results or at least in your supplementary, file but reported in your main text. ANOVA is the basis for establishing variability that is your diversity per se. you have data from three locations, readers will like to see environment, genotype, and their interaction effects prior to be interested in other results. Please include the process in handling these aspects of the analysis in the phenotypic data analysis focusing on choice of model used, justification, and how you generated the data for cluster analysis. You did cluster analysis but you kept information on the data used (BLUES or BLUP) and why?

**Response:** Thank you for your valuable comment. We would like to clarify that the combined ANOVA results were already included in the previous version of the manuscript, specifically under the Results section (Lines 224–231) and also presented in Table 4. The ANOVA table includes the main effects of genotype and environment, as well as the genotype × environment interaction, which form the basis for assessing phenotypic variability and diversity. We updated the Materials and Methods section to clearly explain the statistical model used for the combined ANOVA and the **justification** for model components (Lines 169–178). Best Linear Unbiased Estimates (BLUEs) were computed and used for cluster analysis, because genotypes were treated as fixed effects. This choice is supported by Smith et al. (2005), who recommended the use of BLUEs when genotypes are fixed, particularly in variety testing and diversity studies where specific entries (rather than a random sample from a broader population) are being evaluated.

L135: What do you mean by similarity coefficient? Did you use similarity matrix or dissimilarity matrix for clustering? In my opinion, your focus is diversity so dissimilarity matrix like, Gower and Euclidean are fine.

**Response:** Thank you for this important observation. We agree with your point that a dissimilarity matrix is more appropriate when assessing diversity. Accordingly, we have revised the manuscript to clarify that a dissimilarity matrix was used to construct the hierarchical clustering, specifically employing Euclidean distance combined with Ward’s D² method.

L143: You did PCA with two packages. I am familiar with both packages and can tell that both of them do not do PCA. Only one does the PCA while the other is for visualization. So please be specific which one was used for PCA and which was for visualization.

**Response:** Thank you for pointing this out. We acknowledge the distinction between the functions of the two packages. In the revised manuscript, we have clarified that the FactoMineR package was used to perform the principal component analysis (PCA), while the factoextra package was employed for visualization of the PCA results. This correction has been made in the Methods section to improve clarity and accuracy.

L149: what do you mean immature? I assume you meant fully expanded but young leaf. If so, please correct and if not please clarify.

**Response:** The sentence has been revised as suggested.

L170 – 183: your MAF threshold seems very low (0.01), the standard is usually 0.05. Do you have a reason for bringing it down to 0.01? how did you handle markers with high heterozygosity? Did you check for call rate prior to QC and what was your threshold?

**Response:** Thank you for this insightful comment. We agree that a MAF threshold of 0.05 is commonly used; however, in our study, we intentionally used a more inclusive threshold of MAF ≥ 0.01 to retain rare alleles that may still be informative in capturing genetic variation among soybean genotypes. This approach has also been applied in similar studies in soybean, where SNPs with MAF <1% were removed (Czembor et al., 2021). Regarding heterozygosity, we filtered out SNPs with unusually high heterozygosity using Hardy-Weinberg Equilibrium (HWE)-based filtering in PLINK, considering the self-pollinating nature of soybean and expected inbreeding levels. Additionally, a SNP call rate threshold of 80% was applied (i.e., SNPs with >20% missing data were removed) to ensure the quality of the retained markers.

L177 and 178: You used both VCFtool and PLINK for filtering and marker diversity indices assessment. Please be clear which one is for which activity? BIC is not to estimate but to define optimum subpopulation.

**Response:** We thank the reviewer for this comment. In our revised manuscript, we now clearly indicate that VCFtools was used for SNP-level filtering and PLINK was used to compute diversity indices. Regarding the use of Bayesian Information Criterion (BIC), we agree with your comment and have corrected the statement.

L185 – 196: You cited table 4 before fig 1. Please arrange the results in same manner.

**Response:** We appreciate the reviewer’s observation. In the original submitted manuscript, Table 4 contained the PCA eigenvalues, which preceded Figure 1 (PCA biplot) in the results narrative. In the revised version, Table 4 now presents the combined ANOVA results, and Figure 1 remains the PCA biplot. Despite the change in content, we maintained the original numbering for consistency.

L210: fig 2 should be placed after the text just before table 5.

**Response:** Thank you for bringing this to our attention. We understand the importance of maintaining consistency in the order of figure and table citations. However, we followed the PLOS ONE manuscript submission guidelines, which state that *“Figure captions must be inserted in the text of the manuscript, immediately following the paragraph in which the figure is first cited.”* Accordingly, all figures and tables were positioned based on their first citation in the text, in line with the journal’s requirements. We respectfully retained the current format to comply with PLOS ONE’s instructions, but we remain open to making changes if advised by the editorial office.

L212 – 215: there is grammar issue here, please attend to it.

Response: We appreciate the reviewer’s careful attention to detail. The sentence in question has been revised for grammatical accuracy and clarity in the revised manuscript (Lines 245–248).

L220: Please subject the cluster performance to f-statistics to see whether the cluster groups are significantly different from one another for each of the traits. That way, you can identify which clusters actually possess what traits with statistical confidence.

Response: We thank the reviewer for this insightful suggestion. Accordingly, we subjected the cluster means for each trait to F-tests and the results are presented in the table below (now included as Table 6). The significance column ("Sign") indicates whether cluster differences were statistically significant for each trait, while the least significant difference (LSD) values allow pairwise comparison of means.

L237: Your BIC vs cluster number graph had a sharp drop from one to two and at this point was also the lowest indicating that two was the optimal subpopulation but you said three. Could you justify? I suggest you verify the subpopulation with alternative analysis like PCA scree plot. If it happens that you consider two subpopulations then DAPC may have to change.

**Response:** Thank you for your thoughtful observation. While the BIC plot showed a sharp drop from K = 1 to K = 2, the lowest BIC value did not clearly point to a single best K, as it plateaued between K = 2 and K = 3. Therefore, we considered additional information when determining the optimal number of subpopulations. Using a 70% membership probability cut-off, 27 genotypes were confidently assigned to three distinct subpopulations at K = 3, while 18 genotypes with lower membership probabilities were considered admixed. This level of grouping provided a more informative representation of the population structure.

L249: Fig 5 seems to have been misplaced. The figure was titled phylogenetic three but was placed under population structure. If I am wrong then please provide the results for the phylogenetic analysis. This can be compared to the phylogenetic analysis from the phenotypic data to see how the genotypes grouping aligns.

**Response:** Thank you for the helpful observation. We confirm that Fig 5 is indeed the phylogenetic tree based on SNP marker data, and its placement under the population structure section was intentional, as it visually complements the population structure results. The tree revealed three main sub-populations with high degrees of admixture, which is consistent with the BIC output, thereby reinforcing the robustness of the inferred groupings.

Discussion

L313: The authors said “The minor allele frequency (MAF) values measure the ability of markers to discriminate among genotypes” this is new and contrary to what is known about MAF. If MAF does this, then what is function of PIC?

Response: Thank you for highlighting this important distinction. We agree that our initial wording was unclear. In the revised manuscript, we have corrected the sentence to reflect the role of MAF accurately. The updated sentence now reads: Minor allele frequency (MAF) indicates the prevalence of the less common allele at a locus, helping to distinguish common from rare variants (Kanaka et al., 2023). In contrast, polymorphic information content (PIC) is used to evaluate the informativeness of markers in discriminating among genotypes. The necessary correction has been made in the revised manuscript (Line 492).

Conclusion

The conclusion is too long and needs to be reduced and more concise.

Response: Thank you for the helpful suggestion. In response, the conclusion has been carefully revised to be more concise while retaining the key findings of the study. Please see the revised conclusion in the updated manuscript.

**Reviewer #2**

**1-**Add error1 and error2 as SOV in the ANOVA table

**Response**: We appreciate the reviewer’s valuable suggestion regarding the inclusion of Error 1 and Error 2 as sources of variation in the ANOVA table. Accordingly, we have revised the combined ANOVA table to include Error 1 (variation among replications within locations) and Error 2 (variation among blocks within replications in each location).

**2**-For yield, D50F and D95M traits, you should introduce the superior genotype at each location because the interaction has become significant.

**Response**: We thank the reviewer for this valuable suggestion. We have introduced the superior genotypes for each location based on grain yield (GY), days to 50% flowering (D50F), and days to 95% maturity (D95M), as the genotype x environment interaction was found to be significant for these traits. These values are now presented in a supplementary table (**S5 Table**) and summarized in the Results section (lines 273–281).

**Thank you once again for your valuable comments!**

Sincerely,
Abebawork Tilahun Assfaw on behalf of all co-authors

Reference

Smith AB, Cullis BR, Thompson R. The analysis of crop cultivar breeding and evaluation trials: an overview of current mixed model approaches. The Journal of Agricultural Science. 2005;143(6):449-62.

Czembor E, Czembor JH, Suchecki R, Watson-Haigh NS. DArT-based evaluation of soybean germplasm from Polish Gene Bank. BMC Research Notes. 2021;14(1):343.

Kanaka KK, Sukhija N, Goli RC, Singh S, Ganguly I, Dixit SP, Dash A, Malik AA. On the concepts and measures of diversity in the genomics era. Current Plant Biology. 2023; 33:100278.
