# Supplementary material for: Genetic diversity and population structure analysis of soybean [Glycine max (L.) Merrill] genotypes based on agro-morphological traits and SNP markers
Source: PLoS One. 2025 Oct 10;20(10):e0332895. doi: 10.1371/journal.pone.0332895 (PMC12513657; doi:10.1371/journal.pone.0332895)
Supplement: Attachment — Submitted filename: Response_to_Reviewers_auresp_3.docx [file pone.0332895.s012.docx]

Rebuttal letter

Dear Editor and reviewers,

We would like to express our deepest gratitude to you for taking the time to review our manuscript and providing us with very constructive comments. We truly appreciate it! Below, we provide a detailed point-by-point response to the comments raised by the reviewers. All suggested revisions have been incorporated into the manuscript.

**Reviewer #2**

1-DF=g(r-1)=45*(3-1)=90
I think the DF for Error2 is 90. Please response to this comment.

**Response**: We appreciate the reviewer's observation. The suggested calculation of DF = 90 would indeed be correct if the design were a randomized complete block design (RCBD). However, since our trial was conducted using a 5×9 α-lattice design with 45 genotypes, the block size was k = 5, giving b = g/k = 45/5 = 9 blocks per replication, 3 replications, and 3 locations. The correct calculation for Error 2 is: DF_Error2_ = l × r × (b−1) = 3 × 3 (9-1) = 72

Accordingly, we have corrected the value in the revised ANOVA table (Table 4).

2-For yield, D50F and D95M traits, you should introduce the superior genotype at each location because the interaction has become significant.

**Response**: We appreciate the reviewer’s valuable comment. We would like to respectfully note that this point was addressed in our previously submitted revised manuscript. Specifically, in the Results section (lines 252–260 and **S5 Table**), we have already presented the superior genotypes for grain yield, days to 50% flowering (D50F), and days to 95% maturity (D95M) at each location, in response to this earlier suggestion. We have double-checked the revised version to ensure this information is clearly included.

**Reviewer #3**

Table2: what does the SNP designation in the Table represent?

**Response:** We thank the reviewer for raising this point. In Table 2, two sets of codes are presented for the same soybean genotypes. The Genotype Code (G01–G45) was assigned for use in the phenotypic data analysis to simplify reference to the long pedigrees. The SNP Designation (SY001–SY045) refers to the corresponding codes used for the same genotypes in the SNP-based genetic diversity analysis. Both codes, therefore represent the same set of genotypes but were applied in different parts of the study.

Table 3: the caption should be well-placed on top of the Table and not in the first cell. In addition, add the status of each trait as separate column in the Table (qualitative or quantitative).

**Response:** We appreciate the reviewer's helpful suggestion. We have revised Table 3 accordingly. The caption has been placed at the top of the table, and an additional column has been included to indicate the status of each trait (qualitative or quantitative).

Line 146: linear mixed model is LMM or call it mixed linear model (MLM) not LMER (this is a function in lmerTest R-package).

**Response:** We appreciate the reviewer's comment. We have revised the manuscript to refer to the method as a linear mixed model (LMM) instead of “lmer,” which is a function in the *lmerTest* R package.
Line 157: I do not think LSD can do a good mean comparison for 45 genotypes if you look thoroughly on how it calculates the critical value for comparison. Why not explore HSD that is more robust.

**Response:** We appreciate the reviewer’s suggestion to consider Tukey’s HSD for mean comparisons among the 45 genotypes. While we acknowledge that LSD can be less robust for large numbers of comparisons, it has been widely used in similar crop studies for mean comparisons among many genotypes. For instance, Riyaz et al. (2025) employed LSD to compare 45 common bean genotypes for root and shoot traits under water deficit stress, acknowledging its utility and limitations. Given the context of our study and the straightforward interpretation of pairwise differences provided by LSD, we have retained its use in our analysis.

Line 160 – 165: Kindly include the number of traits used for the cluster analysis and how they were selected. The method is Ward.D2 and not Ward.D2 . cluster analysis is performed using based R function not dendextend package. The package is only used to plot the dendrogram.

**Response:** We appreciate the reviewer's valuable comments. We confirm that all traits recorded during the study were used in the cluster analysis. We have also corrected the description of the clustering method in the manuscript to Ward’s D² method (implemented in R as ward.D2) and clarified that the analysis was performed using the base R function, while the dendextend package was used only for plotting the dendrogram.

Line 168-173: Kindly provide the reason for the PCA analysis. Was it used to select the traits used for clustering? if so, please it should come before cluster analysis.

**Response:** Thank you so much for the constructive comment. The purpose of PCA is described in the Methods section (Lines 171–172). In the revised manuscript, we further clarified that PCA was not used to select traits for clustering (Lines 175–176).

Line 257: you cited S5 Table before S3 Table in line 261 and I think the order should be reversed since it is their first citation in the manuscript

**Response:** Thank you so much. According to the PLOS ONE guidelines, citations of supporting information ***do not need to be in numerical order****.* Therefore, we have retained the current citation order, with S5 Table cited before S3 Table.

Line 370: I think the discussion is too long and contain many redundant statements. Endeavour to reduce the discussion and be more precise.

**Response**: We acknowledge the reviewer’s comment and thank them for it. While the Discussion is somewhat lengthy, this was necessary to address previous reviewer suggestions and elaborate key findings. We believe that the detailed discussion provides valuable context for interpreting the results and supports the robustness of our conclusions.

**Thank you once again for your valuable comments!**

Sincerely,
Abebawork Tilahun Assfaw on behalf of all co-authors

Riyaz I, Zaffar A, Fatima S, Shafi S, Bhat R, Showkat S, et al. Identification of water deficit stress tolerant genotypes of common bean using adaptive root and shoot traits under different screening systems. Scientific Reports. 2025;15(1):19888.
